# Combinatorial interventions inhibit TGFβ-driven epithelial-to-mesenchymal transition and support hybrid cellular phenotypes

**DOI:** 10.1038/npjsba.2015.14

**Published:** 2015-11-26

**Authors:** Steven Nathaniel Steinway, Jorge Gomez Tejeda Zañudo, Paul J Michel, David J Feith, Thomas P Loughran, Reka Albert

**Affiliations:** 1 Penn State Hershey Cancer Institute, Penn State College of Medicine, Hershey, PA, USA; 2 Department of Physics, Pennsylvania State University, University Park, PA, USA; 3 University of Virginia Cancer Center, University of Virginia School of Medicine, Charlottesville, VA, USA

## Abstract

Epithelial-to-mesenchymal transition (EMT) is a developmental process hijacked by cancer cells to leave the primary tumor site, invade surrounding tissue and establish distant metastases. A hallmark of EMT is the loss of E-cadherin expression, and one major signal for the induction of EMT is transforming growth factor beta (TGFβ), which is dysregulated in up to 40% of hepatocellular carcinoma (HCC). We aim to identify network perturbations that suppress TGFβ-driven EMT, with the goal of suppressing invasive properties of cancer cells. We use a systems-level Boolean dynamic model of EMT to systematically screen individual and combination perturbations (inhibition or constitutive activation of up to four nodes). We use a recently developed network control approach to understand the mechanism through which the combinatorial interventions suppress EMT. We test the results of our *in silico* analysis using siRNA. Our model predicts that targeting key elements of feedback loops in combination with the SMAD complex is more effective than suppressing the SMAD complex alone. We demonstrate experimentally that expression of a majority of these elements is enriched in mesenchymal relative to epithelial phenotype HCC cell lines. An siRNA screen of the predicted combinations confirms that many targeting strategies suppress TGFβ-driven EMT measured by E-cadherin expression and cell migration. Our analysis reveals that some perturbations give rise to hybrid states intermediate to the epithelial and mesenchymal states. Our results indicate that EMT is driven by an interconnected signaling network and many apparently successful single interventions may lead to steady states that are in-between epithelial and mesenchymal states. As these putative hybrid or partial EMT states may retain invasive properties, our results suggest that combinatorial therapies are necessary to fully suppress invasive properties of tumor cells.

## Introduction

Epithelial-to-mesenchymal transition (EMT) has important roles in normal physiological processes, such as development and wound healing, and also in pathological processes, such as fibrosis and cancer metastasis.^1,2^ During EMT, cells gain the ability to invade and migrate through a loss of epithelial characteristics, such as tight cellular junctions, and acquisition of mesenchymal attributes, such as increased motility.^1,3^ Loss of E-cadherin, a cell adhesion protein, is considered the hallmark of EMT.

In addition to the epithelial and mesenchymal cellular states, there exists evidence of an intermediate EMT phenotype, known as the hybrid or partial EMT state.^4–6^ The hybrid EMT state is characterized by a mixture of epithelial and mesenchymal features.^6,7^ Like the mesenchymal state, hybrid EMT states may be relevant to pathological processes, such as tumor invasion and metastasis.^8,9^ These hybrid phenotype cells express both epithelial and mesenchymal markers, although there is no clear consensus on the exact characteristics of the hybrid EMT state.^7,10–16^ Evidence suggests that hybrid phenotype cells have combined epithelial (adhesive) and mesenchymal (motility) traits that enable them to migrate collectively, potentially leading to more metastatic potential than the individually migrating cells.^9,17–19^ There is some evidence that suggests that the transition from the epithelial to hybrid state is reversible, whereas the transition from hybrid to mesenchymal state is not.^10,11,20^ The existence of the epithelial, partial EMT and mesenchymal states leads to the hypothesis that complete EMT may be a multistep program.^10,21^ Then the question is how these three states are generated from the regulatory network, and how the system might transition between them. In addition, the EMT network running in individual cancer cells may not be the same even within a single tumor, due to the high levels of genomic instability and heterogeneity characteristic of cancer. In an actual tumor epithelial–mesenchymal ‘tunability’ may represent a mix of genetically or epigenetically non-identical cells. In light of this, a general understanding of the key drivers and blockers of EMT is even more critical.

Because tumor invasion and metastasis are dependent upon EMT, targeting invasion through the EMT process could reduce solid tumor mortality substantially.^8,22,23^ In hepatocellular carcinoma (HCC), for example, invasion is dependent upon EMT,^22^ and the sole available cure for HCC is surgical resection, which is only an option in early stage (non-invasive) disease. Death from HCC is primarily due to late stage disease, which is characterized by invasion, intra- and extrahepatic metastasis, and post-surgical recurrence.^24^ Thus, suppressing invasion and the EMT process could greatly reduce HCC mortality.

Transforming growth factor-β (TGFβ1, hereafter referred to as TGFβ) is one of numerous signals that can induce EMT,^25^ and is frequently dysregulated in HCC.^26–28^ TGFβ signals through a complex network of proteins during the induction of EMT. After binding to the TGFβR complex, TGFβ activates canonical (SMAD) and non-canonical (MAPK and AKT) signaling components. The TGFβ signal converges on a core transcriptional regulatory network (SNAI1, SNAI2, ZEB1, ZEB2, HEY1, FOXC2 and TWIST), which directly represses E-cadherin transcription. The TGFβ pathway signals through many other intracellular proteins, and these interactions form numerous feedback loops and regulatory motifs that drive EMT. Thus, the complexity of the EMT signaling network makes it difficult to fully elucidate using classical experimental dissection.

Mathematical models of various sizes were proposed to study the properties of TGFβ signaling and EMT. Two models focusing on ZEB, SNAI1, miR200, and miR34 have helped elucidate the transcriptional mechanisms driving E-cadherin expression and support the existence of a hybrid EMT state.^11,29^ However, these models consider only a small number of the regulatory components known to be involved in EMT, and thus, they provide only a partial picture. Because of the large number of elements in the TGFβ/EMT network, systems level studies are essential to better understand the dynamics of this complex network.

We previously developed a systems level model of EMT, consisting of 69 nodes and 134 edges, which incorporates a comprehensive map of the growth factors, receptors, signal transductions proteins and transcription factors that regulate E-cadherin expression. Our network model recapitulated the dynamics of TGFβ and EMT signaling. Furthermore, our model suggests that crosstalk with other signaling pathways is critical to the induction of EMT.^21^ Interestingly, our previous investigation suggested that suppression of canonical TGFβ signaling through inhibition of the SMAD complex was insufficient to completely inhibit the TGFβ-driven EMT. We hypothesized that inhibiting the SMAD complex in combination with some other elements can sufficiently suppress EMT. Thus, we used this as motivation to systematically explore knockout perturbations to individual and combinations of nodes in the EMT network to suppress TGFβ-driven EMT, with the ultimate goal of identifying therapeutic interventions that suppress tumor invasion.

We identify through this perturbation analysis 13 single and combined knockout interventions that completely suppress TGFβ-driven EMT in the network model. Interestingly, our results predict that the inhibitory combinations require suppressing the SMAD complex in combination with other nodes. We use this smaller subset of predictions to perform a multi-faceted *in vitro* experimental screen to identify their effect on EMT inhibition. Furthermore, a quantitative analysis of the steady-state landscape obtained by single-node perturbation analysis uncovered steady states that fall into the EMT hybrid steady-state category. We believe these steady states are an extension of the hybrid EMT states previously described both computationally and experimentally.^11,29^

## Results

### Systematic exploration of perturbations in the EMT network reveals that single and combinatorial inhibition of specific nodes can suppress TGFβ-driven EMT

**Figure 1:**
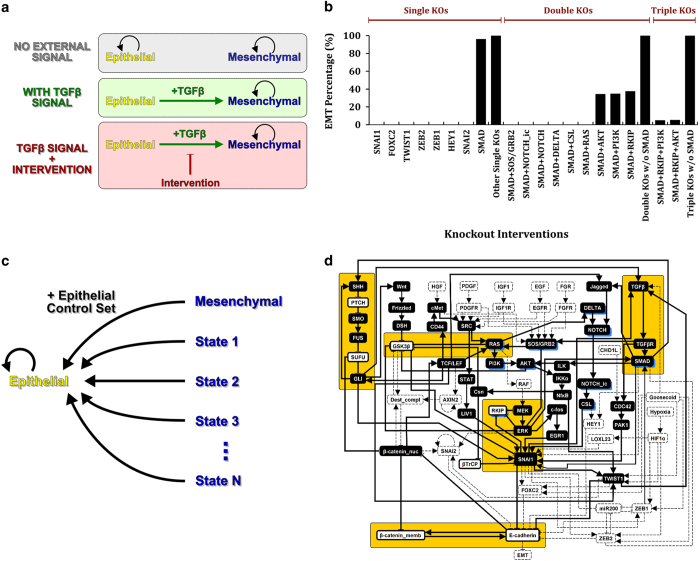
An *in silico* combinatorial knockout screen in the EMT network model reveals specific node combinations that can suppress the TGFβ-driven EMT. A previously constructed network model of EMT was used to identify nodes whose knockout (sustained OFF state) blocked TGFβ-driven EMT. (**a**) Schematic demonstrating that epithelial and mesenchymal cellular states are stable unless an external signal (e.g., TGFβ) or perturbation is applied. Our goal is to identify inhibitory perturbations that block the transition between the epithelial and mesenchymal states even in the presence of TGFβ. (**b**) The effect of knocking out nodes individually and in combinations and their effect on the percentage of EMT in 1,000 simulations per knockout combination. The EMT Percentage is given by the percentage of simulations at the end of which the EMT node is found in the ON state. (**c**) A network control approach was employed to identify epithelial control sets, i.e., set of nodes that when their states are controlled lead to the epithelial steady state. The schematic illustrates the effect of applying the epithelial control set on the network states. (**d**) The stable motif associated with the epithelial steady state. The nodes and edges that are part of the epithelial stable motif have thick lines, while the nodes and edges in the EMT network that are not part of the epithelial stable motif have dashed lines. The epithelial control sets contain one node from each connected group of nodes highlighted by a yellow background. All nodes of the predicted knockout combinations (blue shadow) are part of the epithelial stable motif, and are contained within an epithelial control set or form a path of nodes directly upstream of an epithelial control set. Black or white background on the node symbol indicates that the node is OFF or ON in the epithelial stable motif, respectively. EMT, Epithelial-to-mesenchymal transition, TGFβ, transforming growth factor beta.

Epithelial and mesenchymal cellular phenotypes are stable states that remain as such, unless a perturbation or signal is employed that drives the cells to another state. We previously constructed and validated a dynamic network model of the signal transduction pathways regulating this process and explored the mechanism by which TGFβ drives EMT. A key finding of this work was that TGFβ drives EMT through the activation of multiple other pathways.^21^ The model construction is described in the Results subsections of ref. 21 ‘Constructing the signaling network that drives EMT’ and ‘Translating the EMT signaling network into a dynamic model’. We use our Boolean dynamic model^21^ to systematically explore the effect of individual and combination node knockout perturbations on TGFβ-driven EMT (Figure 1a and Table 1) with the goal of using computationally identified targeting strategies from this screen for experimental validation. We use the computational approach because this kind of screen is intractable through experimentation.

Individual knockout of each node in the EMT network reveals that only targeting one of the seven direct E-cadherin-regulating transcription factors is able to robustly suppress TGFβ-driven EMT (Figure 1b; Supplementary Table 4). This is consistent with the known role of these proteins as transcriptional repressors of E-cadherin expression. Similarly to SMAD in our previous study, no individual node knockout perturbation other than the seven direct E-cadherin-regulating transcription factors could suppress the effect of the TGFβ signal in the EMT network, likely due to EMT-driving network motifs (generalized feedback loops) that remained intact or due to new network motifs arising as a consequence of the perturbation. We hypothesized that a subset of the node perturbations that could not suppress the effect of the TGFβ signal individually could have an effect when applied in combination. Thus, we systematically explored knocking out combinations of two nodes that could not individually fully suppress TGFβ-driven EMT (Table 1). Of the 1,653 possible combinations of two-node knockouts, we identified six two-node knockouts that could fully suppress TGFβ-induced EMT in the network model (Figure 1b; Supplementary Table 5). In addition, we identified three two-node combinations that significantly decreased TGFβ-induced EMT in the model but could not fully suppress it. Interestingly, all these nine combinations require the inhibition of the SMAD complex as one node in the combination (Figure 1b; Supplementary Table 5). For the nodes whose knockout could not fully suppress EMT individually or in combinations of two, we explored combinations of three (Table 1). Of the 22,100 possible combinations of three-node knockout perturbations, none completely inhibited EMT, although some led to a significant suppression of EMT when compared with two-node knockout combinations (Figure 1b and Supplementary Table 6). We next explored knockout combinations of four nodes (270,725 total combinations), and similarly to the three-node combinations, no combinations of four-node knockouts completely suppressed EMT (Supplementary Table 7). Thus, our model suggests that a specific subset of combined node knockouts may suppress TGFβ-driven EMT and these combinations appear exhausted after combinations of two.

Even though we are particularly interested in knockout perturbations (as opposed to constitutive activation perturbations) because of the feasibility of testing them experimentally and implementing them therapeutically, our *in silico* EMT network is not subject to any of these restrictions. For completeness, we also investigate whether any other single-node or double-node perturbations that include constitutive activations can block TGFβ-driven EMT. We find that only one single-node constitutive activation, namely miR200=ON, is able to fully suppress TGFβ-driven EMT (Supplementary Table 8). For the case of perturbations of two elements that could not individually fully suppress TGFβ-driven EMT, we find four new two-node combinations, all of which require the inhibition of the SMAD complex as one node in the combination (Supplementary Table 9). Thus, all two-node interventions of elements that could not individually fully suppress TGFβ-driven EMT require the inhibition of SMAD as one part of the intervention.

### Node combinations identified by systematic perturbation analysis are consistent with a network-motif-based control methodology applied to the dynamic network model

Stable motif analysis is based on the principle that a certain group of nodes forms a network motif (generalized positive-feedback loop) with identifiable properties that stabilizes into a single cellular state regardless of the state of the rest of the network (see Materials and Methods). Thus, a stable motif is a set of elements and their interactions together with the corresponding steady state of each element. As we are currently interested in the network structure that keeps cells epithelial, we applied stable motif analysis to the epithelial steady state. Whereas multiple smaller stable motifs were previously found for the mesenchymal steady state,^21^ we identify a single large stable motif (66% of the network) that stabilizes the epithelial steady state (Figure 1d). This epithelial stable motif involves many of the same elements as the smaller mesenchymal stable motifs, but with the opposite states, and is consistent with our previous finding that each of the mesenchymal stable motifs can drive EMT on their own.

Recently, stable motif analysis has been extended to identify control sets, the sets of nodes and their respective states (i.e., ON or OFF in the Boolean context) that unambiguously lead to a specific steady state regardless of the state of other nodes in the network^30^ (see Materials and Methods). In the context of the EMT model, a set of nodes and their states that force any initial state to evolve toward the epithelial steady state represent an epithelial control set (Figure 1c). The epithelial control sets contain one node from each of the five groups of nodes highlighted by a yellow colored background on Figure 1d; the exact node combinations that form an epithelial control set are given in Supplementary Table 3.

Comparing the epithelial control sets with the results of our systematic perturbation analysis (Figure 1b, nodes with a blue shadow in Figure 1d), we find that they are mutually consistent, that is, the nodes that inhibit TGFβ-driven EMT are part of the epithelial stable motif, and are a subset of an epithelial control set or form a path of nodes directly upstream of an epithelial control set. For example, the knockout combination SMAD+RAS inhibits TGFβ-driven EMT in the model, and these nodes are also part of the twelve epithelial control sets listed in the second row of Supplementary Table 3. Another example is the knockout combination SMAD+DELTA, wherein SMAD is part of the 12 epithelial control sets mentioned before and DELTA is part of a path of nodes (DELTA, NOTCH and NOTCH_ic) upstream of SNAIL1, an element of 45 epithelial control sets, including the 12, which also incorporate SMAD. The consistency between the results obtained by the two methods is not necessarily expected because they address different questions. The systematic perturbation analysis finds the combinations of node interventions that impede the TGFβ-driven transition from an epithelial state to the mesenchymal steady state (Figure 1a, bottom), whereas an epithelial control set finds the interventions in the signal-free EMT network that force any initial state to evolve toward the epithelial state (Figure 1c). Said differently, the first set of interventions can be thought of as a preventive measure, whereas the second set of interventions as a corrective measure. The consistency between the results obtained by the two methods allows us to identify the network mechanisms shared by the two measures and increases our confidence in their potential therapeutic success.

### Mesenchymal phenotype cells are enriched for intervention target nodes predicted by the EMT network model

**Figure 2:**
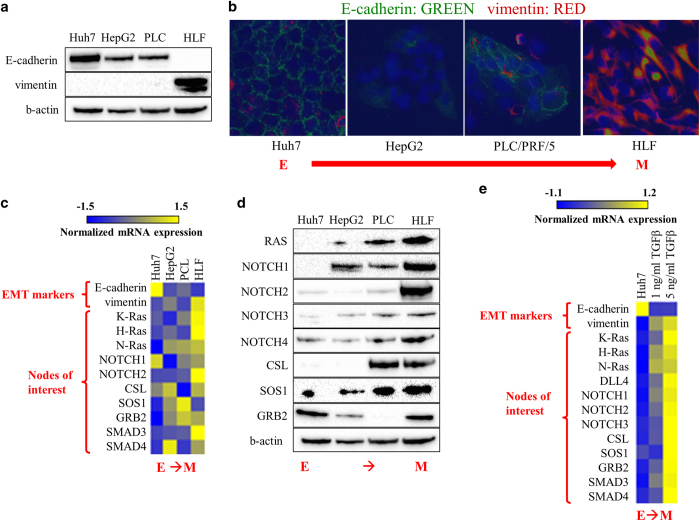
Mesenchymal phenotype cells are enriched for combinatorial intervention target nodes predicted by the EMT network model. (**a**) Expression of epithelial (E-cadherin) and mesenchymal (vimentin) markers in HCC cell lines. (**b**) Immunofluorescent staining of EMT markers E-cadherin (green), vimentin (red), and nuclear stain DAPI (blue) in HCC cell lines Huh7, PLC/PRF/5 (Alexander), HepG2, and HLF. Expression of mRNA (**c**) by qRT-PCR and protein expression by immunoblot (**d**) of nodes whose paired knockout with SMAD is predicted by the EMT network model to inhibit TGFβ-driven EMT. (**e**) Epithelial-like HCC cells Huh7 were treated with TGFβ in serum free media for 48 h (1 ng/ml and 5 ng/ml), then mRNA expression of nodes whose paired knockout is predicted by the EMT network model to inhibit TGFβ-driven EMT was measured by qRT-PCR. EMT, Epithelial-to-mesenchymal transition, HCC, hepatocellular carcinoma, TGFβ, transforming growth factor beta, qRT-PCR, quantitative real-time PCR.

We next sought to experimentally validate predicted inhibitory combinations produced by network model analysis in experimental HCC models. We focused on the set of two-node knockout perturbations, with the ultimate goal of determining whether they inhibit the TGFβ-driven EMT, as predicted by the EMT network model. Prior to testing these inhibitory perturbations experimentally, we sought to determine whether the elements in the node combinations were in fact expressed at appreciable levels in liver cancer cell lines Huh7, HepG2, PLC/PRF/5 and HLF. Previous analysis by immunoblot and quantitative real-time PCR (qRT-PCR) demonstrated that the human HCC cell lines Huh7 and PLC/PRF/5 were more epithelial-like and the HLF cell line had a mesenchymal phenotype.^21^ We confirmed by immunoblot and immunofluorescent staining that Huh7 cells have high E-cadherin and low vimentin expression, whereas HLF cells have low E-cadherin and high vimentin expression (Figure 2a,b). We additionally characterized two other cell lines, PLC/PRF/5 and HepG2, which have intermediate levels of E-cadherin and vimentin (Figure 2b). We tested the expression in these cell lines of the elements that were part of double knockouts predicted to inhibit TGFβ-driven EMT by the model. By qRT-PCR and immunoblot we observed that a majority of the predicted elements were expressed. In addition, by qRT-PCR and immunoblot, the targets of interest were enriched in HLF cell lines compared with Huh7, HepG2, and PLC/PRF/5 cell lines (Figure 2c,d). Furthermore, treating epithelial-like Huh7 cells at a low (1 ng/ml) or high (5 ng/ml) dose of TGFβ for 48 h induced expression of the predicted elements (Figure 2e). Interestingly, the Delta like proteins DLL1 and DLL3 (DELTA node in the EMT model) were not detectable by qRT-PCR in the Huh7 cell line, and DLL4 was only detectable at very high amplification cycles after TGFβ treatment by qRT-PCR (Figure 2e), suggesting that the Delta ligands are not appreciably expressed in the Huh7 cell line. We were also unable to detect Delta proteins in HepG2, HLF and PLC/PRF/5 cell lines. These results suggest that all intervention targets predicted by the EMT network model other than Delta ligands are expressed in experimental cell line models and are also enriched in mesenchymal relative to epithelial phenotype cell lines.

### A multi-faceted siRNA screen of predicted node knockout combinations *in vitro*

**Figure 3:**
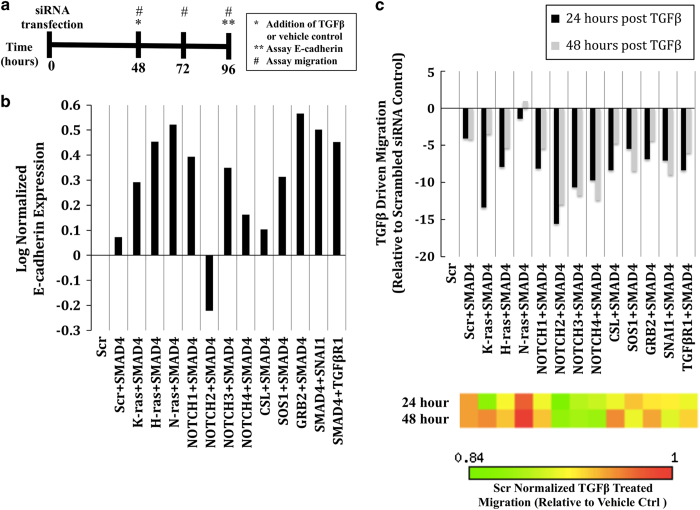
A multi-faceted siRNA screen to test predicted node knockdown combinations *in vitro.* (**a**) Schematic of the experimental design of the screen to test node knockdown combinations that are predicted to inhibit TGFβ-driven EMT. At 0 h, epithelial-like Huh7 cells were transfected with siRNA combinations or a scrambled siRNA control (2 nM per siRNA), and then plated for harvesting of protein, mRNA, and cell migration. At 45 h post transfection, cells were serum starved for 3 h. At 48 h, cells were treated with TGFβ (5 ng/ml) or a vehicle control for an additional 48 h. At 96 h, cells were harvested for mRNA and protein expression. An aliquot of siRNA-transfected cells were plated at 0 h in 96-well plates for assessment of migration with the Oris Cell Migration Assay (see Materials and Methods for details). These cells were treated with either TGFβ or a vehicle control at 48 h. Simultaneously, migration stoppers were removed at 48 h post transfection and analysis of cell migration was performed at 48, 72, and 96 h post transfection. (**b**) The effect of model-predicted node knockout combinations on E-cadherin expression in TGFβ-treated cells relative to vehicle control treated cells, as measured by quantitative immunoblotting. (**c**) The effect of model-predicted node knockout combinations on *in vitro* cell migration in TGFβ-treated cells relative to vehicle control treated cells. The percent change in TGFβ-driven migration (top) and heat-map of TGFβ-driven migration (bottom) relative to a scrambled siRNA control 24 and 48 h after TGFβ treatment. EMT, Epithelial-to-mesenchymal transition, mRNA, messenger RNA; siRNA, small interfering RNA, TGFβ, transforming growth factor beta.

Systematic analysis of knockout perturbations in the *in silico* EMT network suggests that targeting a specific subset of elements individually or in combination may suppress TGFβ-driven EMT (Figure 1b). In order to test these combinations experimentally, an small interfering RNA (siRNA) screen was employed using an experimental approach that mimicked the *in silico* analysis (Figure 3a, see Materials and methods for details). E-cadherin expression and cell migration were used as experimental readouts for EMT.

The effect of siRNA combinations on TGFβ treated Huh7 cells relative to vehicle-treated control cells normalized to a scrambled siRNA control was assessed via quantitative immunoblotting and qRT-PCR (Figure 3b, Supplementary Figure 2, and Supplementary Figure 3). Combinations of SMAD4 with K-RAS, H-RAS, N-RAS, NOTCH1, NOTCH3, and GRB2 were most similar to the positive controls (SNAI1 plus SMAD4, TGFβR1 plus SMAD4) that lead to high expression of E-cadherin (epithelial phenotype); thus these combinations agree with the model predictions. Combinations of SMAD4 with NOTCH2 and CSL were most similar to the negative controls (scrambled siRNA, SMAD4 alone) that lead to low expression of E-cadherin (mesenchymal phenotype). These combinations did not have the effect predicted by the model. We note that the prediction that combined SMAD and NOTCH inhibition leads to high expression of E-cadherin is overall verified because of the NOTCH1 and NOTCH3 results.

To assay for cell migration, automated image acquisition and analysis was used to quantify cell migration at 48, 72, and 96 h post transfection (Figure 3a). The percent change in TGFβ driven migration for siRNA combinations compared with scrambled control was calculated (Figure 3c). Combinations of SMAD4 with K-RAS, NOTCH2, NOTCH3, and NOTCH4 were most similar to the positive controls representing lower migratory capacity; thus, these are in agreement with the model predictions. Combination of SMAD4 with N-RAS did not inhibit EMT, which was not expected from the model.

The results of combined inhibition of SMAD with KRAS, HRAS, NOTCH1, NOTCH3, NOTCH4, SOS1, or GRB2 are consistent across the two measures and agree with the theoretical predictions. Two combination knockouts had opposing effects on measures of EMT: knockdown of SMAD4 with NOTCH2 showed low E-cadherin expression, while being effective in suppressing cell migration, whereas knockdown of SMAD4 with N-RAS showed high E-cadherin expression, but also retained higher migratory capacity.

### Analysis of stable motifs that arise due to SMAD inhibition in the EMT network reveals an attractor landscape that is distinct from the SMAD unperturbed network

**Figure 4:**
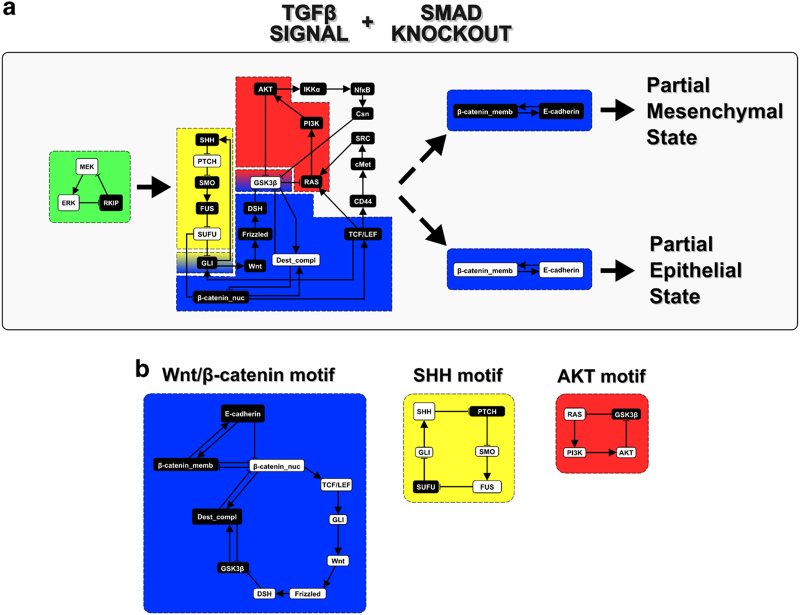
Analysis of network motifs that arise after SMAD inhibition in the EMT network reveal an attractor landscape that is distinct from the unperturbed EMT network. The TGFβ-driven SMAD-perturbed EMT network (the EMT network with fixed TGFβ=ON and SMAD=OFF) has an almost identical mesenchymal state as the unperturbed network, as well as epithelial and mesenchymal states that are relatively different from the epithelial state of the unperturbed EMT model. We identify these relatively different states as hybrid epithelial-mesenchymal states. (**a**) The stable motifs associated with the hybrid epithelial–mesenchymal steady states and the sequence in which they stabilize after the TGFβ signal and SMAD suppression. The color of the node background indicates the node state; black corresponds to OFF and white corresponds to ON. The stable motif in the middle contains subsets of the stable motifs associated to the mesenchymal steady state (shown in **b**) but with the opposite states. These motifs are the Wnt/β-catenin stable motif (blue background), the AKT stable motif (red background), and the SHH stable motif (yellow background). (**b**) The stable motifs associated with the mesenchymal steady state in the TGFβ-driven SMAD-perturbed (TGFβ=ON; SMAD=OFF) EMT network and in the unperturbed EMT network. The color of the box that contains the stable motifs is the same as the background color used in **a**. EMT, Epithelial-to-mesenchymal transition, TGFβ, transforming growth factor beta.

Results from the systematic network perturbation analysis and subsequent experimental work suggest a pivotal role for the SMAD complex in TGFβ-driven EMT. Exploration of the network attractors achievable under the TGFβ stimulus and in the absence of the SMAD complex reveals a mesenchymal steady state that is essentially the same as the one in the TGFβ-driven unperturbed network and as the original unperturbed network (see Supplementary Table 2 and Supplementary File 1). Surprisingly, we also find a new epithelial and a new mesenchymal steady state, which are similar to each other but relatively different from both the epithelial state and the mesenchymal state of the unperturbed model (Figure 4a; steady states 113 and 118 in Supplementary File 1). These new steady states contain some features of the epithelial steady state and some features of the mesenchymal steady state of the unperturbed model. The epithelial (putative hybrid) steady state in the TGFβ-driven SMAD-perturbed network contains a subset of the unperturbed epithelial stable motif, which keeps E-cadherin=ON and β-catenin_memb=ON while keeping the SHH, AKT, and Wnt motifs inactivated. This steady state also has some mesenchymal features including activated MEK, ERK and SNAI1 (Figure 4a,b). The new mesenchymal (putative hybrid) steady state also has the SHH, AKT, and Wnt motifs inactivated, as well as activated MEK, ERK, and SNAI1, but E-cadherin=OFF & β-catenin_memb=OFF instead of ON. Supplementary Figure 1 shows the stable motif succession diagram of the TGFβ-driven SMAD-perturbed network, indicating the trajectories through which the sequential stabilization of stable motifs leads to the mesenchymal, partial mesenchymal, or partial epithelial steady state.

### Quantitative analysis of steady states in perturbed EMT networks reveals a putative spectrum of EMT phenotypes

**Figure 5:**
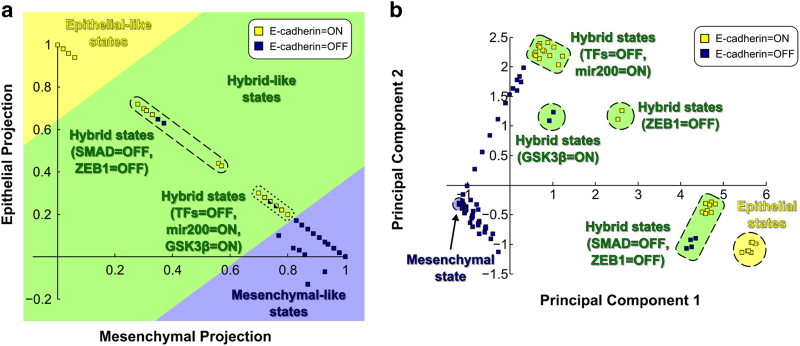
Quantitative analysis of the steady states associated to single-node perturbations in the EMT network model supports the existence of an EMT spectrum. Single-node knockouts and constitutive activations were performed in the TGFβ-driven EMT network model. The steady states from each of these perturbed networks were projected onto the epithelial and mesenchymal steady states from the unperturbed EMT network (**a**), and onto the first and second principal components obtained by principal component analysis (**b**). Each steady state is represented by a square whose color denotes the presence (yellow) or absence (blue) of E-cadherin. Steady states that have the same coordinates when projected in the epithelial/mesenchymal plane but differ in the node state of E-cadherin are represented by a square with both yellow and blue color. The background color of the marked groups denotes the type of steady state (epithelial-like, yellow; hybrid-like, green; or mesenchymal-like, blue). Groups of states are labeled by the node whose knockout or overexpression lead to this state; the abbreviation ‘TFs’ in the group labels represents any of the transcription factors FOXC2, HEY1, TWIST1, SNAI1, SNAI2, ZEB1, or ZEB2. (**a**) In the epithelial/mesenchymal plane, the epithelial and mesenchymal states corresponds to (1, 0) and (0, 1) in the *x*–*y* plane, respectively. Quantitative analysis of the steady states in single-node perturbed models reveals a spectrum of steady states, many of which are intermediate to the unperturbed epithelial and mesenchymal steady states. (**b**) In the principal component plane, the epithelial steady states from the unperturbed EMT network model cluster in the bottom right corner of the plot and the mesenchymal steady states, along with numerous other perturbed states, cluster in the bottom left corner of this plot. Numerous other steady states exist in distinct and intermediate clusters between the epithelial and mesenchymal clusters. EMT, Epithelial-to-mesenchymal transition, TGFβ, transforming growth factor beta.

We identified all of the attractors obtained by applying single-node perturbations to the TGFβ-driven EMT network. All of these attractors were found to be steady states, i.e., no complex attractors were found. To quantitatively classify these steady states relative to the epithelial and mesenchymal steady states of the unperturbed EMT network, they were projected onto the sub-state-space spanned by the unperturbed (or normal) epithelial and mesenchymal states. This projection is represented in a mesenchymal/epithelial plane, where the normal mesenchymal state has coordinates (mesenchymal, epithelial)=(1,0), and the normal epithelial state has coordinates (mesenchymal, epithelial)=(0,1) (Figure 5a). The single-node perturbed TGFβ-driven EMT network steady states are located mostly along the diagonal that joins the normal mesenchymal and the normal epithelial state, which indicates that these steady states are proper combinations of the epithelial and mesenchymal steady states. In other words, in these steady states each individual node’s state matches this node’s state in either the epithelial steady state, the mesenchymal steady state, or both (for nodes whose state is the same in both steady states). A closer look at the mesenchymal/epithelial diagonal reveals a spectrum of steady states that start at each of the opposite ends of the diagonal with a gap between them in which two small subsets of isolated steady states are located. These small subsets of isolated steady states are located near the middle of the diagonal, in-between the mesenchymal and epithelial states. Thus, we interpret them as partial EMT or hybrid epithelial/mesenchymal states. Consistent with this, the previously described attractors of the TGFβ-driven SMAD-perturbed network, which were different from both the epithelial and the mesenchymal states, are part of the small subset of isolated steady states.

We also tested whether this classification between epithelial, mesenchymal, and hybrid states depended on our *a priori* specification of mesenchymal/epithelial states. To do this, we performed principal component analysis using the steady states of all single-node perturbed TGFβ-driven EMT networks and the unperturbed epithelial and mesenchymal steady states (see Materials and Methods). A visual comparison between the epithelial/mesenchymal projection and the first principal component projection confirms that the steady states cluster into the same groups, a finding which we also verify using hierarchical clustering (Supplementary Figure 4, Supplementary Text). A projection onto the first and second principal components also shows a clear separation between the mesenchymal, epithelial, and hybrid states and additionally reveals a structure of subgroups (Figure 5b, Supplementary Figure 4). For example, the cluster marked ‘TFs=OFF, miR200=ON’ (TFs represent any of the transcription factors FOXC2, HEY1, TWIST1, SNAI1, SNAI2, ZEB1, or ZEB2) is connected to the mesenchymal cluster by a dense group of perturbed steady states composed of steady states associated to the single-node perturbations that blocked EMT (i.e., knockout of the E-cadherin-regulating transcriptional factors and constitutive activation of miR200). This group of steady states is characterized by having mostly mesenchymal features, with the exception of E-cadherin, its transcriptional regulators, and β-catenin activity. The clusters of hybrid states highly disconnected from the mesenchymal states, e.g., the one marked ‘SMAD=OFF, ZEB1=OFF’ are associated to the knockout of SMAD or ZEB1, and contain several mesenchymal (e.g., active MEK, ERK, and SNAI1) and epithelial features (e.g., inactive WNT, SHH, and Jagged).

## Discussion

EMT has a crucial role in cancer invasion and metastasis. TGFβ is a signal for induction of EMT and is a frequent dysregulation in HCC.^26,28^ Here we set out to explore perturbations to the EMT signaling network that are predicted to increase the efficacy of SMAD complex inhibition (i.e., canonical TGFβ signaling) in order to inhibit the induction of EMT. To accomplish this and answer the broader question of how to inhibit EMT, we systematically screen individual and combination node knockout perturbations in the EMT network to identify nodes and/or combinations of nodes that, when knocked out, suppress the TGFβ-driven EMT (Table 1 and Figure 1b). To better understand the mechanism through which the combinatorial interventions suppress EMT, we compare them with the intervention targets obtained from a network control approach^30^ that identifies the feedback regulatory motifs (called stable motifs) through which the epithelial phenotype is maintained (Figure 1c,d). We test these combinations experimentally, with the results suggesting that many of the interventions predicted to inhibit EMT *in silico* suppress the TGFβ-driven EMT *in vitro* (Figure 3). Finally, we analyze the SMAD-perturbed EMT network model and find that some steady states are significantly different from both the epithelial and mesenchymal steady states of the unperturbed EMT network (Figure 4). This finding led us to quantitatively explore the steady states of other perturbed networks. Our results reveal numerous steady states that are intermediate between epithelial and mesenchymal steady states, suggesting the existence of hybrid (i.e., partial EMT) states, with the SMAD-perturbed steady states being examples of such hybrid states. Further analysis revealed the stable motifs responsible for this hybrid epithelial–mesenchymal state (Figure 4) and the existence of an epithelial–mesenchymal spectrum (Figure 5; Supplementary Figure 4). The existence of the epithelial, hybrid EMT, and mesenchymal states demonstrated in our work and others’^10,21^ leads to the hypothesis that complete EMT may be a multistep program. The fact that tumors are made up of genetically or epigenetically non-identical cells makes identification of initial condition independent ways of controlling cellular state even more critical.

One advantage of using a computational model is that we were able to screen hundreds of thousands of knockout perturbations rapidly and with essentially no cost. The same screen experimentally would require extensive time, resources, and cost. This screen provides a much smaller subset of targets that can then be taken through experimental analysis. Knockout of seven nodes individually and six nodes in combinations of two suppressed EMT in our network model. The seven individual nodes (SNAI1, SNAI2, ZEB1, ZEB2, TWIST, FOXC2, and HEY1) represent the network of transcription factors that directly repress E-cadherin transcription. This is consistent with literature evidence for the role of these transcription factors in the induction of EMT.^10,11,31–33^ We find that SMAD complex inhibition in combination with inhibition of RAS, NOTCH, or SOS1/GRB2 was able to suppress the TGFβ-driven EMT both *in silico* and *in vitro*. Indeed, our experimental measurements of E-cadherin expression and also suppression of migration, a functional readout of EMT, support that these combinations are superior to SMAD inhibition alone. This suggests that suppression of non-canonical signaling in addition to canonical (SMAD) signaling is required for robust inhibition of EMT. TGFβ can signal non-canonically to activate NOTCH, and the cross-talk between the TGFβ and NOTCH signaling pathways appears to be critical to EMT signal transmission.

There exists previous evidence of TGFβ and NOTCH cross-talk during the induction of EMT. Initial studies demonstrated that TGFβ signaling can increase expression of the NOTCH signaling pathway target gene HEY1/HES, and that TGFβ induced EMT was blocked by suppressing HEY1,^32^ which is consistent with our model (Figure 1b). Additional evidence suggests that Notch signaling is necessary for epithelial growth arrest by TGFβ in multiple epithelial cell types^34^ and that Jagged1 and HEY1 were induced by TGFβ in kidney epithelial cells.^35^ Our experimental and computational results suggest that SMAD4 inhibition has some effect alone but that combined NOTCH and SMAD suppression has an increased effect on EMT inhibition, which has not been described previously.

Interestingly, DELTA ligands (DLL1, DLL3, and DLL4) were undetected in our cells, yet Notch signaling was activated and had a functional role in TGFβ-driven EMT. Because Delta-Notch signaling acts through a paracrine signaling mechanism, it is likely that in order to activate Notch in HCC^36^ Delta is expressed on neighboring cells in the tumor microenvironment that are not present in HCC cell lines (e.g., tumor vasculature)^37^ whereas the NOTCH ligand Jagged may be expressed in neighboring HCC cells. Studies in murine lung adenocarcinoma cells support that Jagged2 is important for TGFβ-driven EMT and metastasis.^38^ This suggests that the Jagged ligand could have a role in the TGFβ-driven EMT in liver cancer cells. Jagged is included in our model and is activated in the mesenchymal state, as seen in Figure 4a of our previous work,^21^ but its knockout is not predicted to inhibit TGFβ-driven EMT in our models. Preliminary studies suggest that Jagged2 is expressed in the Huh7 cell line and that Jagged2 expression is increased by TGFβ treatment (Supplementary Figure 5). The role of Jagged2 in the TGFβ-driven EMT in HCC is the subject of ongoing/future studies. If Jagged is found to have a critical role in TGFβ-driven EMT in liver cancer cells, then the model could be revised to account for this.

There exists some variation between combinations predicted to inhibit EMT computationally versus the effect of these combinations experimentally. Variability in siRNA efficiency (Supplementary Figure 2) could potentially lead to some false negatives in our study (i.e., combinations tested for EMT suppression that do not appear to suppress EMT). Alternative explanations for predicted versus actual effects of combinatorial inhibitions could be at play as well. For example, several of the node knockout combinations contain nodes that are represented by protein families: RAS; DELTA; and NOTCH. Knockout of the node in the model is equivalent to knocking down all the members of the family, which is not feasible experimentally. Thus, if a gene family existed in the model, then each family member was separately targeted experimentally, which could be a limitation of the experimental screen.

There exists limited evidence about the role of specific RAS family members in HCC EMT, although the evidence suggests that K-RAS and H-RAS activation have a role in HCC EMT and metastasis.^39,40^ Not much is known about the different roles of NOTCH family members in cancer, although there is speculation that they have different roles in brain tumor development.^41^ There exists evidence that each of the NOTCH family members contributes to hepatocarcinogenesis. NOTCH1,^42^ NOTCH3,^43^ and NOTCH4 (ref. 37) have all been associated with poor prognosis in HCC, including larger tumor size and metastasis, although one study suggests a dominant role for NOTCH1 over NOTCH3.^42^ Other evidence suggests that blocking NOTCH signaling in liver cancer cells using a pan-NOTCH γ-secretase inhibitor suppresses cell proliferation, and this appears to be independent of NOTCH1 dysregulation.^36^ Further evidence suggests that NOTCH2 on its own can drive HCC growth in human and mouse models of HCC and in patients.^43,44^

In our experiments knockdown of NOTCH1, NOTCH3, and NOTCH4 each increased E-cadherin expression and suppressed cell migration, whereas NOTCH2 had an opposing effect on E-cadherin protein expression in the Huh7 cell line. Interestingly, NOTCH2 knockdown did appear to increase E-cadherin messenger RNA (mRNA) expression (Supplementary Figure 3) and to suppress TGFβ-driven migration *in vitro*. One thing that is not considered in our model is the effect that knocking down these proteins has on other features of cancer cells such as cell proliferation or apoptosis, as these outcomes and their related signaling pathways and transcriptional regulators are not included in the EMT network. If NOTCH2 is knocked down in the EMT network and NOTCH2 knockdown also leads to apoptosis, then the E-cadherin loss could be because of general protein degradation during apoptosis.

Our perturbation analysis and the quantitative comparison of the perturbed steady states with the epithelial and mesenchymal states indicated that many perturbations lead to steady states that are intermediate to the epithelial and mesenchymal states. There exists evidence of hybrid EMT states in experimental and computational models;^4–6,11,29^ however, to our knowledge, a quantitative characterization of an epithelial–mesenchymal spectrum has not previously been done. Both of the two previous computational models predicted a single hybrid state, likely due to the fact that only a small subset of the known EMT regulators were included in these models.^11,29^ Our model suggests that looking at a larger subset of regulators could be useful in a quantitative description of the EMT spectrum. The ZEB1 knockout appears to induce hybrid steady states in the EMT network model (Figure 5), and this is consistent with previous findings.^10,11^ The miR200=ON steady-state represents another interesting hybrid EMT state. Interestingly, previous work suggests that miR200 overexpression had no effect on the metastatic features of a HCC cell line; however, combination with a DNA methyltransferase (DNMT) inhibitor led to suppression of metastasis *in vivo*.^45^ This suggests the DNMT inhibitor is targeting other nodes in the EMT network, leading to a more robust epithelial state.

The quantitative steady state analysis enriches the picture offered by our knockout studies. For example, knockout of one of the seven transcription factors is predicted to obstruct EMT (i.e., to maintain high E-cadherin expression, Figure 1b) but according to our steady-state analysis it leads to a partial epithelial state that also has several mesenchymal features (Figure 5). Convergence to the epithelial steady state of the unperturbed network requires the implementation of a complete epithelial control set (Figure 1d and Supplementary Table 3), for example, keeping TGFβR=RAS=SHH=MEK=OFF and β-catenin_memb=ON. A less comprehensive combinatorial intervention will likely lead to a hybrid epithelial state, wherein E-cadherin and other epithelial features are present but some other elements are in their mesenchymal states. At present it is not known to what extent such an intermediate state may be relevant to migration and metastasis, but intermediate states could explain differences in invasive/metastatic capacity across individual tumors or cell lines.

In summary, we used Boolean dynamic modeling and network analysis to investigate the role of SMAD dynamics in the TGFβ-driven EMT, with the goal of identifying ways to inhibit EMT in HCC. Our systems biology approach was able to use the available pathway information to identify putative targets that suppress HCC EMT. *In vitro* analysis indicated that many of the predicted interventions suppress the TGFβ-driven EMT, and thus, are potential therapeutic targets for suppressing features of invasive tumors. Future work will focus on in depth experimental analysis of these targets including exploring their effect on invasion *in vivo*.

## Materials and methods

### Cell culture

Huh7 cells were provided by Dr Harriet Isom (Penn State College of Medicine, Hershey, PA, USA).^46^ HepG2 and PLC/PRF/5 (Alexander) cells were acquired from the American Tissue Culture Collection. HLF cells were acquired from Dr Jorge Filmus (University of Toronto, Toronto, ON, Canada).^47^ PLC/PRF/5 cells were acquired from the ATCC (Manassas, VA, USA). All cells were cultured in Dulbecco's modified Eagle's medium medium (Life Technologies) +10% fetal bovine serum (Atlanta Biologicals, Norcross, GA, USA). TGFβ treatments were completed with recombinant human TGFβ (Peprotech, Rocky Hill, NJ, USA). Complete cell culture media was removed and cells were washed with phosphate-buffered saline. Cells were serum starved by incubating them in serum free growth media for 3 h, followed by addition of TGFβ to the serum-free media at specified doses. Cells were maintained in a humidified 5% CO2 37 **°**C incubator. TGFβ1 was reconstituted at 20 μg/ml in sterile 4 mM HCl containing 1 mg/ml or bovine serum albumin. As 5 μg/ml TGFβ was used (a 1:3 dilution in serum-free cell culture media), the vehicle control used in experiments was 4 mM HCl containing 1 mg/ml or bovine serum albumin diluted 1:3 in serum-free cell culture media. Huh7 and HLF cell lines were authenticated using short tandem repeat DNA profiling (Genetica DNA laboratories, Cincinnati, OH, USA) on 20 January 2014. HepG2 and PLC/PRF/5 cells were received directly from the American Tissue Culture Collection and cultured for <6 months so no authentication was deemed necessary.

### siRNA transfections

siRNA transfections were performed in Huh7 cells to screen the effect of *in silico* predicted node knockdown combinations in TGFβ induced EMT compared with TGFβ untreated controls. Invitrogen Silencer Select siRNAs (Life Technologies, Frederick, MD, USA) were used to specifically target genes or a scrambled siRNA-negative control (#4390846). 2 nM of each siRNA (4 nM total for combinations of two nodes)and 5 μl Lipofectamine RNAiMAX were added to 1 ml Opti-MEM I medium (Life Technologies, Frederick, MD, USA) without serum, then combined with 7.5×10^5^ cells in 5 ml complete media. Cells were then plated for analysis of mRNA, protein, and cell migration. Further details of the siRNA transfections are included in the Supplementary Text.

### Quantitative real-time PCR

The Ambion Cells-to-C_T_ Kit (Life Technologies, Frederick, MD, USA) was used to extract RNA and reverse transcribe RNA to complementary DNA for qRT-PCR without having to purify RNA prior to amplification as per manufacturer’s instructions. qRT-PCR experiments were conducted using a CFX384 Touch Real-Time PCR Detection System (Bio-rad, Hercules, CA, USA) and TaqMan Universal PCR Master Mix (Life Technologies, Frederick, MD, USA). The housekeeping genes GAPDH and β-actin were used for ΔΔCt calculations and relative expression was calculated using Taqman primer/probe sets (Life Technologies, Frederick, MD, USA).

### Immunoblot analysis

Immunoblot analysis was performed as previously described.^21^ Protein bands were analyzed and quantified using the Image Lab software suite (Bio-rad, Hercules, CA, USA). Details of antibodies used and Immunoblot quantification is described in the Supplementary Text.

### Detection of cell migration using automated cell imaging acquisition and analysis

The Oris Cell Migration Assay (Platypus Technologies, Madison, WI, USA) was used to assess the effect of *in silico* predicted combinatorial node knockouts on TGFβ-driven cell migration. Details of the Oris cell migration assay and image-based quantification of cell migration are described in detail in the Supplementary Text.

### Network modeling framework and dynamic model simulations

We use a Boolean framework in which each network node is described by one of two qualitative states: ON or OFF. The ON (logical 1) state means an above threshold activity or abundance, whereas the OFF (logical 0) state means below-threshold activity or abundance. The biological relationships among nodes in the EMT network are expressed as mathematical equations using Boolean operators.^21,48^ The Boolean equations governing the dynamics of the EMT network model are the same as in ref. 29 and are reproduced in Supplementary Table 1. The EMT network model has a group of steady states that match the epithelial or the mesenchymal phenotype, respectively, and that we identify as the epithelial or the mesenchymal state, both of which we reproduce in Supplementary Table 2. The specifics of the Boolean modeling simulation including the ranked stochastic asynchronous updating scheme are described in detail in the Supplementary Text.

### Perturbation analysis

To capture the effect of knocking out or externally supplying elements in the network model, modification of the states/rules to describe knockout or overexpressed states were performed. These modifications were implemented by setting the corresponding state of the nodes to either OFF (knockout) or ON (constitutive activation) and keeping the selected node state fixed (i.e., ignoring the corresponding updating rules for these nodes) during the simulations. The key outcome of the simulation of each perturbation is the percentage of simulations that reach the ON state of the node EMT at the end of the simulation, which we refer to as the percentage of EMT or EMT%. The outcome of a perturbation can vary from one simulation to another because of the stochastic nature of the updating scheme. By examining many such forced perturbations, we can identify potential therapeutic strategies, many of which may not be obvious or intuitive, particularly for large and complex networks.

### Stable motif analysis

Stable motif analysis is used to identify a set of function-dependent network components of a Boolean network known as stable motifs. Stable motifs were introduced in ref 49 and consist of a set of nodes and their node states, where the nodes form a strongly connected component (a composition of intersecting directed feedback loops) and the node states are partial steady states of the Boolean network.

The stable motifs of a Boolean network have been shown to be closely related to the network’s attractors (steady states or complex attractors, see Supplementary Text). Stable motifs act as ‘points of no return’ in the dynamics of a network, and thus, a network state that matches the node states specified by a given stable motif is guaranteed to be in the basin of attraction of the attractor(s) that also match the node states specified by the given stable motif. In fact, one can uniquely associate sequences of stable motifs in a Boolean network to one of its attractors through a stable motif succession diagram, a diagram composed of all the sequences of stable motifs and their associated attractors (e.g., Supplementary Figure 1; see ref. 30 for details). This diagram reflects the possible trajectories in which the system can converge to an attractor through the sequential stabilization of stable motifs, either autonomously or being driven externally. The left-most part of a stable motif succession diagram contains a group of starting stable motifs, each of which has one or more outgoing directed arrows that lead to either other stable motifs or to an attractor, the latter of which are located in the right-most part of the figure. Starting from any of the motifs in the left-most part of the figure and following any of the directed arrows until one of the attractors is reached, one obtains an ordered sequence of stable motifs and the unique attractor associated to this ordered sequence.

Here we use this methodology to identify the stable motifs associated to the epithelial steady state (Figure 1d) and the stable motifs associated to each of the steady states of the TGFβ-driven SMAD-perturbed EMT network (Figure 4 and Supplementary Figure 1). A detailed description of this methodology and adaptation to finding the stable motifs of a specific steady state are described in the Supplementary Text.

### Attractor analysis

In order to identify the attractors of the Boolean models encountered in this work, we use the attractor-finding method described in ref. 49, which uses the stable motifs of a Boolean model to identify its attractors. For most contexts studied in this work (including most of the single-node perturbed TGFβ-driven EMT networks) the attractor-finding method can be applied directly, thus guaranteeing that all the attractors are found. Three exceptions (two single-node perturbed EMT networks and the unperturbed EMT network) took a prohibitively long amount of time and required using an approximation on the attractor-finding method, which we describe in the Supplementary Text. For all cases, all attractors were found to be steady states, i.e., no complex attractors were found. The mesenchymal and epithelial steady states are reproduced in Supplementary Table 2, and the list of all steady states identified in the single-node perturbed TGFβ-driven EMT networks and in the unperturbed EMT network model are given in Supplementary File 1.

### Network-control methodology

We employed a recently developed methodology that identifies specific nodes whose control (e.g., externally imposed sustained inactivity or high activity) leads to a specific cellular state.^30^ This control methodology, known as stable motif control, uses the correspondence between sequences of stable motifs and attractors in order to identify control sets (each a set of nodes and their associated node states) that drive any initial condition to a target attractor. Stable motif control sets are obtained by identifying the core of the sequences of stable motifs that lead to an attractor, and thus, not all stable motifs in a motif sequence and not all nodes in a stable motif are part of a control set. These control sets are guaranteed to drive an arbitrary Boolean network state to the attractor of interest with 100% effectiveness and only need to be applied transiently to the network in order to drive it toward the target attractor.^30^ Furthermore, subsets of the full control sets have been found to be sufficient to either completely drive any network state to the target attractor or, at least, to greatly increase the percentage of network states that evolve toward the target attractor (i.e., to increase the basin of attraction of the target attractor). Here we use the stable motif-control methodology to identify control sets that drive the EMT network model toward the epithelial steady state.

For the epithelial steady state only one stable motif was found (Figure 1d), which we call the epithelial stable motif. The stable motif control method uses the epithelial stable motif to derive the stable motif control sets for the epithelial steady state, which we denote epithelial control sets (Figure 1c and Supplementary Table 3). The identification of stable motif control sets was performed in Java using the StableMotifs Java library (available at https://github.com/jgtz/StableMotifs).

### Epithelial/mesenchymal plane projection

The EMT network model we previously developed^21^ has a steady state that matches the epithelial phenotype and that we identify as the epithelial state (see Supplementary Table 2). Under TGFβ induction (i.e., setting TGFβ=ON in the model) the epithelial state is driven to a steady state that matches the mesenchymal phenotype and that is also a steady state in the EMT network model without TGFβ induction, which we identify as the mesenchymal state (see Supplementary Table 2).

In order to quantitatively classify the steady states of the TGFβ-driven, single-node perturbed EMT networks, we project each steady state obtained on the sub-state-space spanned by the unperturbed epithelial and mesenchymal steady states using the method in ref. 50. The projection of a state on the mesenchymal (or epithelial) state is denoted by *a* ^M^ (or *a* ^E^). Under this type of projection, the projection of the epithelial state on the mesenchymal state is zero, and vice versa. Hence, in a mesenchymal/epithelial plane, where *a* ^M^ is the horizontal coordinate and *a* ^E^is the vertical coordinate, the mesenchymal state has coordinates (*a* ^M^, *a* ^E^)=(1,0), and the epithelial state has coordinates (*a* ^M^, *a* ^E^)=(0,1). To classify the steady states as epithelial-like, hybrid-like or mesenchymal-like we use the location of the steady states positioned along the diagonal of the mesenchymal/epithelial plane: epithelial-like if *a* ^E^−*a* ^M^>0.65, hybrid-like if −0.65<*a* ^E^−*a* ^M^<0.65, and mesenchymal-like if *a* ^M^−*a* ^E^>0.65. See the Supplementary Text for more details.

### Principal component analysis

Principal component analysis (PCA) is used to reduce a complex (high dimensional) data set to a lower number of dimensions, which often reveals patterns underlying the data.^51^

In our work, we employ PCA of 162 steady states (the steady states of the unperturbed EMT network and of each single-node perturbed TGFβ-driven EMT network), each of which consists of 69 EMT network nodes in the steady state (Supplementary File 1). PCA is performed using MATLAB’s *princomp* function;^52^ a more detailed explanation of PCA is described in the Supplementary Text.

## Figures and Tables

**Table 1:** Results of the EMT network model knockout perturbation analysis

*No. of nodes in each knockout*	*No. of nodes considered*	*Total possible combinations*	*No. of combinations that completely block EMT*
1	65	65	7
2	58	1,653	6
3	52	22,100	0
4	52	270,725	0

Abbreviations: EMT, Epithelial-to-mesenchymal transition, TGFβ, transforming growth factor beta.

TGFβ, TGFβR, E-cadherin and EMT nodes are not considered because these target either the input signal (TGFβ) or the output node of interest (E-cadherin), which makes the number of nodes we consider in the perturbation analysis 65 instead of the total of 69 nodes. In addition, nodes whose individual knockout blocks EMT are not considered for double knockouts, and pairs of nodes whose combined knockout blocks EMT are not used as part of triple or quadruple combinations.
