# Supplementary material for: Combinatorial interventions inhibit TGFβ-driven epithelial-to-mesenchymal transition and support hybrid cellular phenotypes
Source: NPJ Syst Biol Appl. 2015 Nov 26;1:15014–. doi: 10.1038/npjsba.2015.14 (PMC5516807; doi:10.1038/npjsba.2015.14)
Supplement: Supplementary Information [file npjsba201514-s1.doc]

**Supplementary Information**

**Supplemental Text. Supplemental text of materials and methods.**

*Immunoblot analysis and quantification*

Primary antibodies for E-cadherin, Vimentin, RAS, NOTCH1, NOTCH2, NOTCH3, NOTCH4, CSL, SOS1, GRB2, and β-actin (Cell Signaling Technology, Danvers, MA) were used for this study at a final dilution of 1:1000. Quantified E-cadherin bands for each siRNA knockdown combination (Figure 3B) were normalized to the β-actin band (protein loading control). Next, for each siRNA knockdown sample, E-cadherin in TGFβ treatment samples relative to TGFβ untreated samples was calculated. Finally E-cadherin in TGFβ treatment with respect to TGFβ untreated samples was calculated relative to the scrambled control, giving the effect of node knockdown combination on TGFβ driven EMT relative to the effect of TGFβ on EMT markers with a scrambled siRNA control. All protein bands were within the linear range as determined by Image Lab.

*siRNA transfections to test in silico* predictions

In order to test the knockout combinations, siRNA to each target element was transfected into Huh7 cells in the combinations predicted by the network model or with a scrambled siRNA control. At 48 hours post transfection, cells were treated with TGFβ or with a vehicle control. At 96 hours post transfection, cells were harvested to analyze the effect of siRNA node knockdowns on the epithelial phenotype marker E-cadherin (Figure 3A). siRNA transfection efficiency for the different genes targeted is shown in Supplemental Figure 2. The average knockdown efficiency across all siRNAs is 70%; the three lowest efficiencies are for NOTCH3 (45%), NOTCH1 (47%), and SOS2 (59%).

Two negative controls were used to represent ineffective inhibition of EMT (low E-cadherin expression and higher migratory capacity). The first was a scrambled siRNA transfection to control for effects of siRNA transfection. In the second, only SMAD4 siRNA was used, as network analysis suggested that SMAD4 inhibition alone minimally inhibits the TGFβ-driven EMT. Two positive controls were used to represent effective inhibition of EMT (high E-cadherin expression and lower migratory capacity). The first was siRNA targeting SNAI1 plus SMAD4 because literature evidence and network analysis suggest that SNAI1 inhibition robustly suppresses TGFβ-driven EMT. The second was siRNA targeting TGFβR1 plus SMAD4, as this would directly disrupt TGFβ signaling at its source.

*Detection of cell migration using automated cell imaging acquisition and analysis*

The Oris Cell Migration Assay was used to quantitate cell migration. The assay comes in 96 well plates with stoppers, which exclude cells from attaching to the centers of the well. Once the stoppers are removed, a cell-free zone (called the detection zone) is revealed into which cells can migrate. Following siRNA transfections (described above), Huh7 hepatocellular carcinoma cells were plated in wells of Oris Cell Migration Assay 96 well plates. Cells were plated at a density of 1.5x10^4^ per well, and placed at 37**°**C in a humidified 5% CO_2_ incubator for 48 hours. At 48 hours, migration stoppers were removed from all the wells, and cells were stained with a whole cell fluorescent stain CellTracker Green Dye (2.5 μM; Life Technologies, Frederick, MD) as per the manufacturer’s recommendations. The whole cell fluorescent stain was used for fluorescent imaging of cells to quantify migration at multiple time points throughout the experiment. After staining, cells were serum starved for three hours. For wells in the TGFβ treatment group, TGFβ was added to wells at a final concentration of 5 ng/ml. Twelve replicate wells were used per transfection: six for TGFβ treatment and six for control cells untreated with TGFβ. At 0, 24, and 48 hours after stoppers were removed (48, 72, and 96 hours post transfection), cells were imaged with the Cytation 3 automated fluorescent cell imager (Biotek, Winooski, VT). Cells were imaged using a 4X objective and GFP filter. Four images were taken with auto exposure in two by two dimensions such that the entire detection zone and surrounding well area were captured. The four images were spliced together using Gen5 version 2.0.7 Software (Biotek, Winooski, VT) in order to construct a continuous image of the detection zone and surrounding area for image based quantification of the detection zone area using ImageJ.

*Quantification of cell migration with ImageJ*

Cell migration was assessed by measuring the area of the detection zone at the pre-migration and 24 and 48 hour migration time points using ImageJ 1.48v analysis software [1] by modifying a protocol as previously described [2]. To measure the area of the detection zone from fluorescence images, the image scale was set using the known micron/pixel values (Analyze-->Set Scale). Next, the threshold was set to enhance the contrast between the detection zone and the cell monolayer (Image-->Adjust-->Threshold). Manual adjustment of the threshold was completed in selected cases where the software chosen threshold did not correctly identify the detection zone boundary. By selecting “Apply” in the threshold window, the thresholded image was converted to a binary image. The Wand (tracing) tool was clicked on the Detection Zone until the region was outlined (indicated by a yellow trace). Using the menu command Analyze-->Set Measurements, the “Area” and “Display Label” were selected. Finally, the menu command Analyze-->Measure was selected to calculate the detection zone area. The “Area” measurements for each well at each time point were used to calculate the percent migration:


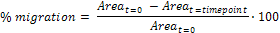


The mean percent area closure was calculated from the six replicate wells. The effect of each siRNA knockdown combination on TGFβ-driven migration was determined by calculating the percent area closure in TGFβ treated siRNA knockdown combinations relative to cells with siRNA knockdown combination that were not treated with TGFβ.


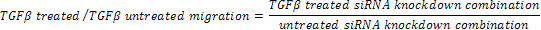


The TGFβ treated relative to TGFβ untreated migration for each node knockdown combination was normalized to scrambled siRNA control TGFβ treated relative to untreated percent area closures. This normalization scheme allows us to know the effect of node knockdown combinations on TGFβ-driven migration relative to a scrambled siRNA control sample.

*Differentiating between cell migration and cell proliferation in the migration assay*

In order to limit the effect of cell proliferation on the readout of migration, anti-proliferative agents are used at appropriate doses to suppress proliferation of cells without leading to cell death. Strategies frequently used in the literature include chemical inhibitors (e.g. mitomycin c) or reducing the amount of serum in the media. Because the TGFβ treatment strategy requires a period of serum starvation and also requires that TGFβ treatment happen in serum free media, we chose to use serum free media as a means to suppress proliferative effects on the migration assay. We tested various serum concentrations to identify an appropriate dose of serum in the Huh7 cell line media, which normally gets 10% serum (specifically, fetal bovine serum-FBS) by volume. The dose response included 10%, 5%, 1%, 0.75%, 0.5%, 0.25%, 0.1%, and 0% FBS. We measured cell proliferation out to 72 hours and we found that including 0% FBS suppressed Huh7 cell proliferation substantially (by 50%) although it did not completely abrogate it.

*Boolean model simulation*

Simulations of the Boolean dynamic model are performed by applying the Boolean functions in succession. Boolean models and discrete dynamic models in general focus on state transitions in discrete time steps instead of following the system in continuous time. The network starts from an initial condition (initial state of the EMT network elements) and transitions through several states until it reaches a set of states in which it settles down, known as an attractor. An attractor can be a fixed point (steady state) or a set of states that repeat indefinitely (a complex attractor). The EMT network model has a group of steady states that match the epithelial or the mesenchymal phenotype. We identify these states as the epithelial or the mesenchymal state, respectively. Formally, we find one steady state that can be identified as the mesenchymal state and 8 steady states that can be identified as epithelial states; these 8 steady states only differ in the state of three nodes that contain an activating self-loop. We choose as the bona fide epithelial state the one that most closely matches the epithelial phenotype. The epithelial and mesenchymal states are reproduced in Supplemental Table 2.

We made use of a ranked general asynchronous (stochastic) updating scheme to simulate network dynamics. This scheme is based on the general asynchronous updating scheme, in which at each time step (*t*+1), the state of a randomly selected node (
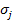
) is updated based on the state of its *n* regulators at a previous time point (*t*) and according to a Boolean function (
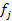
) that captures the biological relationships among the regulators:


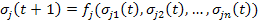


The Boolean functions of the EMT network model are the same as in [3] and are reproduced in Supplemental Table 1. We employ a ranking system by updating nodes regulated by signal transduction events with a greater probability than nodes regulated by transcriptional events in order to account for signal transduction events occurring faster than transcriptional events, as previously described [3]. Nodes within each category (signal transductions events or transcriptional events) are all updated with equal probability. The employed updating scheme does not affect the fixed points identified by our model, because fixed points are independent of the implementation of time; however, we previously found that this updating scheme more accurately represented real dynamics [3]. In each situation studied, 1,000 simulations were performed using the epithelial state (Supplemental Table 2) as the initial condition, and following the system for at least 10,000 time steps, which ensured that a steady state is reached. The key outcome of the simulations is the percentage of simulations that reach the ON state of the node EMT at the end of the simulation, which we refer to as the EMT percentage. The simulations of the model were performed in Java using the BooleanDynamicModeling Java library (available at https://github.com/jgtz/BooleanDynamicModeling).

*Stable motif analysis*

Stable motifs were introduced in [4] and consist of a set of nodes and their node states
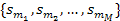
, where the nodes form a strongly connected component (a composition of intersecting directed feedback loops) and the node states are partial steady states of the Boolean network, that is,


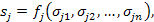


where
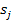
 is the state of node
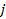
 in the stable motif (
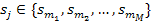
),
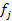
 is its associated Boolean function, and
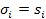
 if node
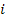
 is specified in
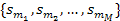
, otherwise
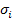
 can be any node state. One can uniquely associate sequences of stable motifs in a Boolean network to one of its attractors [5]. In [3] we used this methodology to identify the stable motifs associated to the mesenchymal steady state of the EMT network model.

In order to identify stable motifs, we use the method introduced in [4], which maps the problem of identifying stable motifs to finding specific strongly connected components. The strongly connected components of interest have certain identifiable properties in an expanded representation of the Boolean network which includes the network’s Boolean functions as part of the network structure [4, 6]. In this expanded network representation, every node of the original network is associated to two nodes in the expanded network, one of which denotes the ON state of the original node while the other denotes the original node’s OFF state. Additionally, a new set of expanded network nodes known as composite nodes is added, which captures the combinatorial nature of the logical functions associated to each node in the original network. A detailed explanation of the criteria for identifying stable motifs and of the procedure for creating the expanded representation of the Boolean network can be found in [4]. For a more formal and mathematical discussion of stable motifs see S3 Text of ref[5] or Appendix A of ref [4]. The stable motif analysis was performed using the StableMotifs Java library (available at <https://github.com/jgtz/StableMotifs>).

In some Boolean networks, the number of cycles involved in the stable motif search and/or the size of some of the associated stable motifs can be quite large, which does not allow for an exact enumeration of stable motifs. Since the most computationally expensive step of stable motif identification algorithm involves the combination of cycles to form potential stable motifs, introducing a cutoff for the length of the directed cycles can reduce the run time of the algorithm without necessarily leading to loss of information. For a discussion of the time complexity and mitigation techniques for the identification of stable motifs see S3 Text of ref [5].

For the current analysis of the epithelial stable motif, the stable motif algorithm was adapted to the case when one has a known steady state of interest. Here the known steady state of interest is the epithelial steady state. This adaptation was required because the number of cycles involved in the epithelial stable motif search and the size of the stable motif did not allow a direct identification of the stable motif with the StableMotifs Java library, even though a cutoff size for the directed cycles was used to construct the stable motifs.

In order to identify the epithelial stable motif, we take advantage of the fact that the epithelial steady state is known. Specifically, we first reduce the unperturbed EMT network using the reduction method described in [4]. This reduction method traces down the effect of sustained input signals and simplifies nodes with one input or one output [4, 7], and has been proven to conserve all attractors. Since stable motifs are defined only after the effect of inputs signals has been considered and since the topology of stable motifs (strongly connected components) is unchanged by the node simplification procedure of the reduction method [4, 7], use of the network reduction method does not lead to any loss of generality. The next step in identifying the epithelial stable motif is to create its expanded network representation [4, 7], and then restrict the expanded network to the expanded network nodes that are consistent with the states of the epithelial steady state (e.g. E-cadherin=ON in the epithelial state so we take only the expanded network node corresponding to E-cadherin=ON). The restricted expanded network is such that it conserves any stable motif in which all of its associated node states are the same as in the steady state used to construct the restricted expanded network; this can be shown from the definition of a stable motif in terms of the expanded network (i.e., stable motifs are strongly connected components in the full expanded network with certain identifiable properties).

Using the restricted expanded network, we search for stable motifs using the StableMotifs Java library and a cutoff of 10 for the length of the directed cycles used to construct the stable motifs. This allows us to identify the epithelial stable motif in the reduced EMT network, which the reduction method allows us to map back to the epithelial stable motif of the original EMT network.

*Modification of the attractor analysis approach*

The stable-motif-based attractor finding methodology for Boolean models was used to find all the network attractors [4]. There were two exceptions where this methodology needed to be modified. The exceptions to this were a pair of single-node perturbed TGFβ-driven EMT networks (PAK1=OFF and CDC42=OFF) and the unperturbed EMT network, for which the number of cycles involved in the stable motif search and/or the size of some of the associated stable motifs were too large. For example, the stable motif associated with the epithelial state of the unperturbed EMT network included 66% of the network. For these cases we used a cutoff of 10 for the length of the directed cycles used to construct the stable motifs associated to the attractors.

*Robustness of the EMT network model results to timescale variations*

The EMT network model uses an updating scheme that updates nodes regulated by signal transduction events more frequently than nodes regulated by transcriptional events, and thus, considers two different timescales. In our previous work we found that this updating scheme more accurately reproduced the real dynamics. This updating scheme is still a simplification of the real dynamics, in which the timescale associated to every element can be very diverse and may not be accurately captured by the use of two timescales. This gives rise to the question of whether the diverse timescales present in the real system will have a strong effect on the results and conclusions of this work.

Most of the results presented in this work are independent of the of the timescales involved because they are related to the steady states and the stable motifs of the model, both of which are independent of the updating probabilities of each node (5). Specifically, the stable motif analysis, the epithelial control set, and the analysis of the steady states in the perturbed and unperturbed EMT networks are all independent of the timescales. The only results that could depend on the updating probabilities of each node are the EMT percentages in the perturbation analysis (Figure 1B, Supplemental Tables 4-9). We test whether the EMT percentages in the perturbation analysis for single perturbations, double perturbations, and triple knockouts are noticeably changed by assigning an equal update probability to each node. We use the same number of simulations (1,000) and time steps (10,000) in the equal updating probability simulations as in the ranked update probability simulations. We find that the results are qualitatively similar and that our conclusions still hold.

Specifically, the scenarios that lead to EMT percentages of 0% or 100% are identical for both updating schemes. Supplemental Table 10 shows the scenarios that display a noticeable change in the EMT percentages (where the magnitude of the relative change (EMT%_EQPROB_ - EMT% _RANKED_)/EMT%_RANKED_ was greater than 0.2). The only scenarios that display a noticeable change in the EMT percentages are six double perturbations (out of 6,612) and twenty-three triple knockouts (out of 22,100).

*Principal component analysis*

Principal component analysis (PCA) is a tool used in statistical analysis of high-dimensional systems that provides a non-parametric way to reduce a complex dataset to a lower dimension, which often reveals patterns underlying the data [8].

Given a dataset composed of
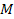
 observations of
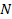
 variables, PCA uses the variance and covariance of each of the
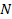
 variables among the
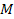
 observations to find the directions in the *N-*dimensional space in which the variables vary the most, which are called the principal components. The principal components are numbered in descending order of variance, such that principal component
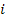
 is the
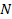
-dimensional direction with the
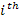
 largest variance. By projecting each of the original observations into the first
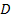
 principal components one can obtain lower dimensional (
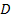
-dimensional) representation of the original observations that highlight the directions of largest variance in the data.

In our work, the dataset is composed of
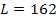
 steady states (the steady states of the unperturbed EMT network and of each single-node perturbed TGFβ-driven EMT network), each of which consists of the node state of the
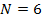
9 EMT network nodes in the steady state (Supplemental File 1). PCA is performed using MATLAB’s *princomp* function [9] giving as an input a matrix with components
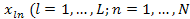
), where
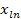
 takes the value 1 or 0 depending on if the state of node
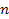
 in the steady state
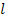
 is ON or OFF, respectively. The eigenvalues associated to the principal components are shown in Supplemental Figure 6.

*Epithelial/mesenchymal plane projection*

The EMT network model we previously developed [3] has a steady state that matches the epithelial phenotype and that we identify as the epithelial state. Under TGFβ induction (i.e., setting TGFβ=ON in the model) the epithelial state is driven to a steady state that matches the mesenchymal phenotype and that is also a steady state in the EMT network model without TGFβ induction, which we identify as the mesenchymal state. The epithelial and mesenchymal states are reproduced in Supplemental Table 2.

In order to quantitatively classify the steady states of the TGFβ–driven, single-node perturbed EMT networks, we project each steady state obtained on the sub-state-space spanned by the unperturbed epithelial and mesenchymal steady states using the method in [10]. To do this, we express each steady state
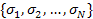
 as a vector
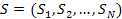
 whose
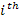
 entry takes the value +1 if the
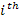
 node state is ON and -1 if the
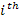
 node state is OFF. Using the representation of steady states as vectors of +1’s and -1’s, we can use the method in [10] to project an arbitrary state into the sub-state-space spanned by the mesenchymal vector state
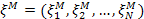
 and the epithelial vector state
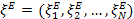
. The projection of a state
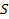
 on the mesenchymal (or epithelial) state is denoted by
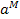
 (or
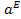
) and is defined by


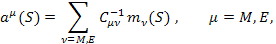


where


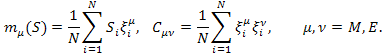


Under this type of projection, the projection of the epithelial state on the mesenchymal state is zero, and vice versa. Hence, in a mesenchymal/epithelial plane, where
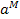
 is the horizontal coordinate and
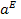
is the vertical coordinate, the mesenchymal state has coordinates (
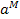
,
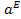
) = (1,0), and the epithelial state has coordinates (
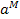
,
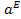
) = (0,1).

To classify the steady states as epithelial-like, hybrid-like, or mesenchymal-like we use the location of the steady states positioned in the diagonal of the mesenchymal/epithelial plane. Specifically we classify a steady state as epithelial-like if
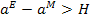
, hybrid-like if
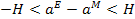
, and mesenchymal-like if
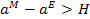
, with
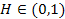
 and use
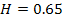
. This corresponds to steady states near the diagonal being classified as epithelial-like if
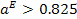
, mesenchymal-like if
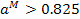
, and everything in-between as hybrid-like. The motivation behind the choice of
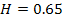
 to classify states is the steady states associated to TFs=OFF, miR200=ON that form a cluster in the principal component plane. This cluster is close to the group of steady states associated to the mesenchymal steady state and yet has the main epithelial marker E-cadherin=ON. This suggests that the TFs=OFF, miR200=ON cluster of steady states should be part of the hybrid-like states or close to the boundary separating them from the mesenchymal-like states. A lower and more stringent value of
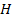
 would classify some of the hybrid states associated to TFs=OFF, miR200=ON, and GKS3β=ON as mesenchymal-like, since they are at the border of the mesenchymal-like/hybrid-like states with
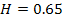
. The steady states associated to SMAD=OFF and ZEB1=OFF would require an even more stringent value of
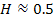
 to no longer be classified as hybrid, and some of these steady states would require an even lower value of
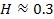
 to no longer be classified as hybrid.

*Hierarchical clustering*

Hierarchical clustering is a data analysis method to group a set of objects into clusters using a specified measure of similarity between the objects and based on a criterion for cluster formation. The result of hierarchical clustering is commonly expressed as a dendrogram, which tracks the aggregation of objects into clusters as the sensitivity of the clustering criterion is changed.

In our work, hierarchical clustering is used on the Epithelial/Mesenchymal plane projection, on principal component 1, and on principal components 1 and 2 of the dataset of
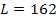
 steady states (the steady states of the unperturbed EMT network and of each single-node perturbed TGFβ-driven EMT network). To do this, we use MATLAB’s *pdist*, *linkage*, and *dendrogram* functions using Euclidian distance as the similarity measure, Ward’s method as the cluster formation criterion, and a maximum number of clusters of 12 [9].

**Supplemental References**

**
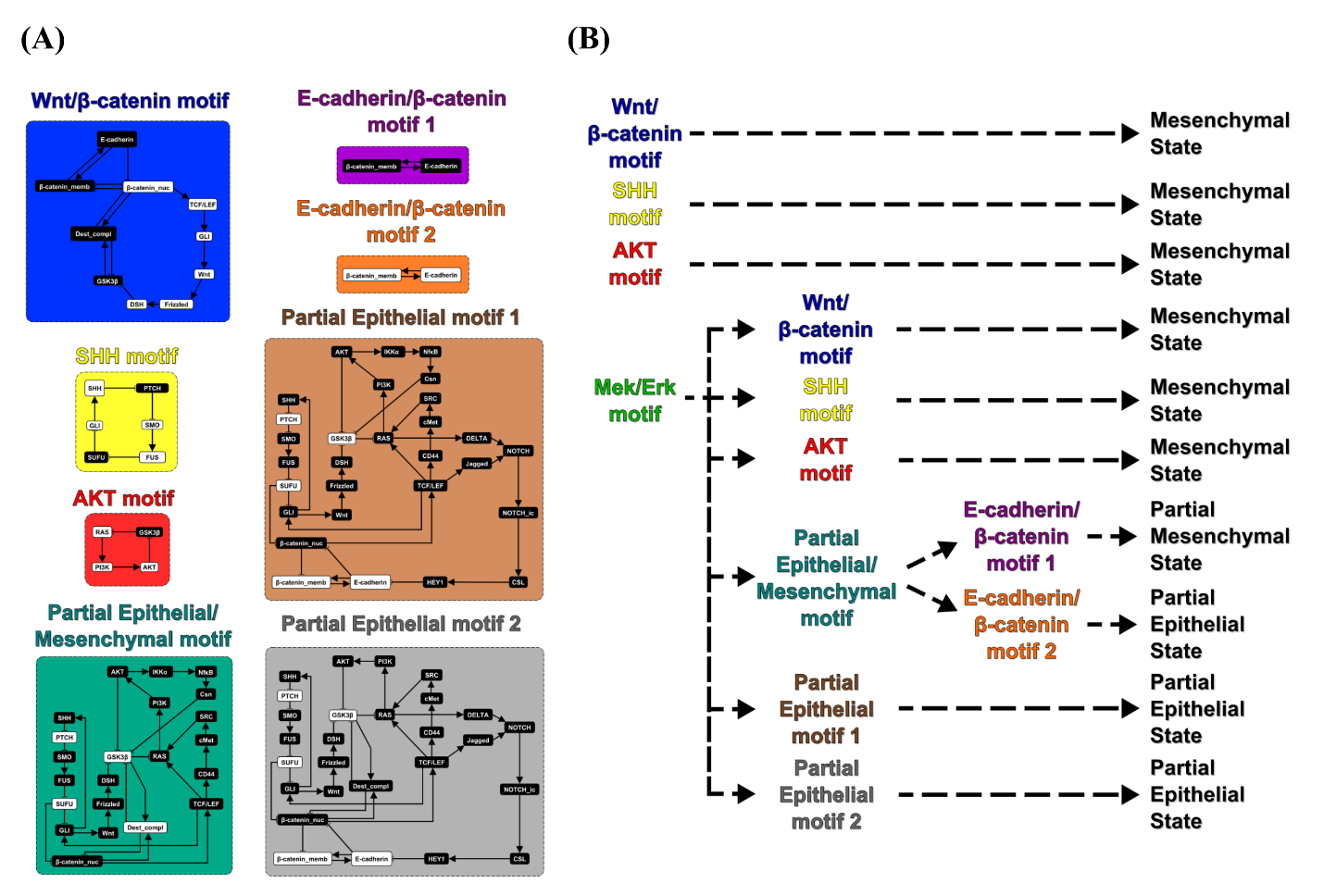
**

**Supplemental Figure 1: Stable motifs and stable motif succession diagram for the TGFβ-driven SMAD-perturbed EMT network model.** (A) All the stable motifs of the TGFβ-driven SMAD-perturbed EMT network model. The background color of a node corresponds to the OFF (black) or ON (white) state of the node in the stable motif. The background color of the box containing the motif matches the color of the name of the motif, which is used to represent the stable motif in the motif succession diagram in panel B. (B) The stable motif succession diagram for the TGFβ-driven SMAD-perturbed EMT network model (see Methods). The Wnt/β-catenin, AKT and SHH stable motifs are uniquely associated to the mesenchymal steady state; these three stable motifs are also mesenchymal stable motifs of the unperturbed EMT network model. The diagram also shows that the activation of the MEK/ERK motif is necessary, but not sufficient, to obtain any of the hybrid (partial) steady states.

**
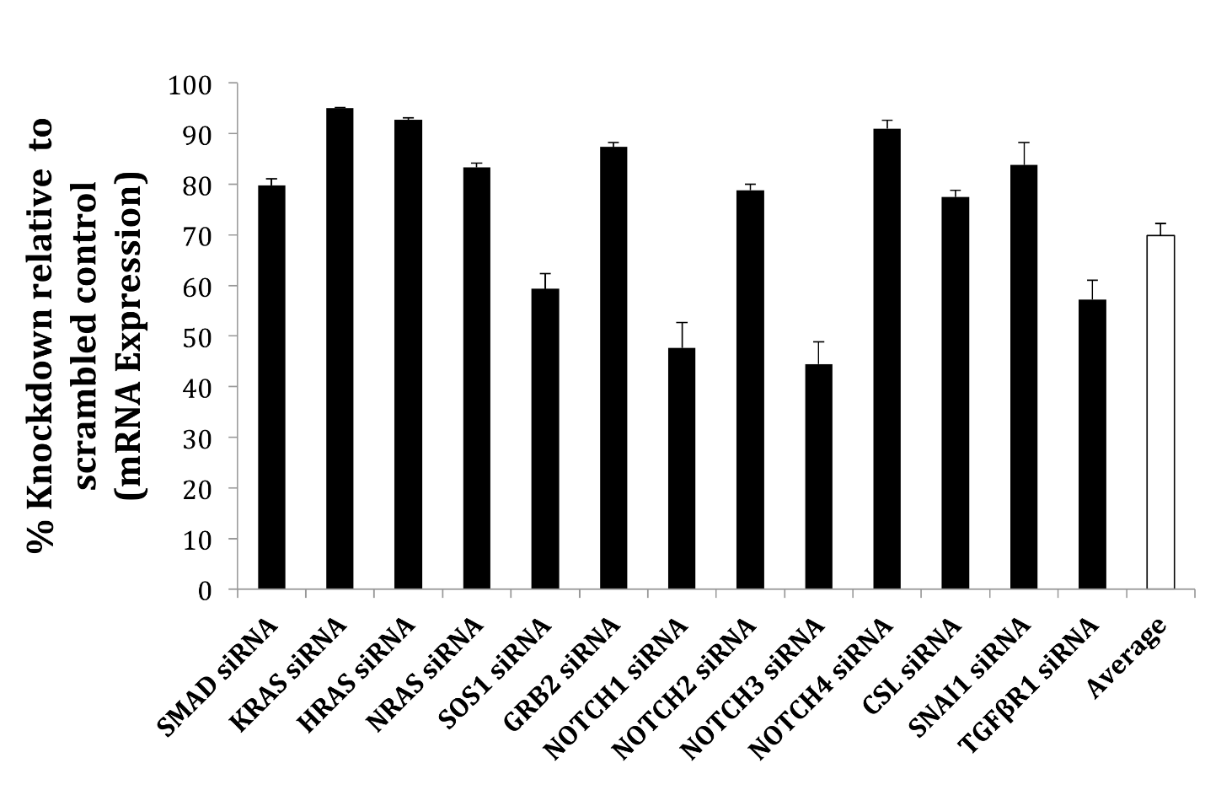
**

**Supplemental Figure 2: Node knockdown efficiency for the siRNAs used.** Knockdown efficiency was assessed relative to an siRNA control for each siRNA used in the screen. The percent knockdown of node mRNA expression by qRT-PCR is shown at 96 hours post transfection. The average knockdown efficiency across all siRNAs is 70%.

**
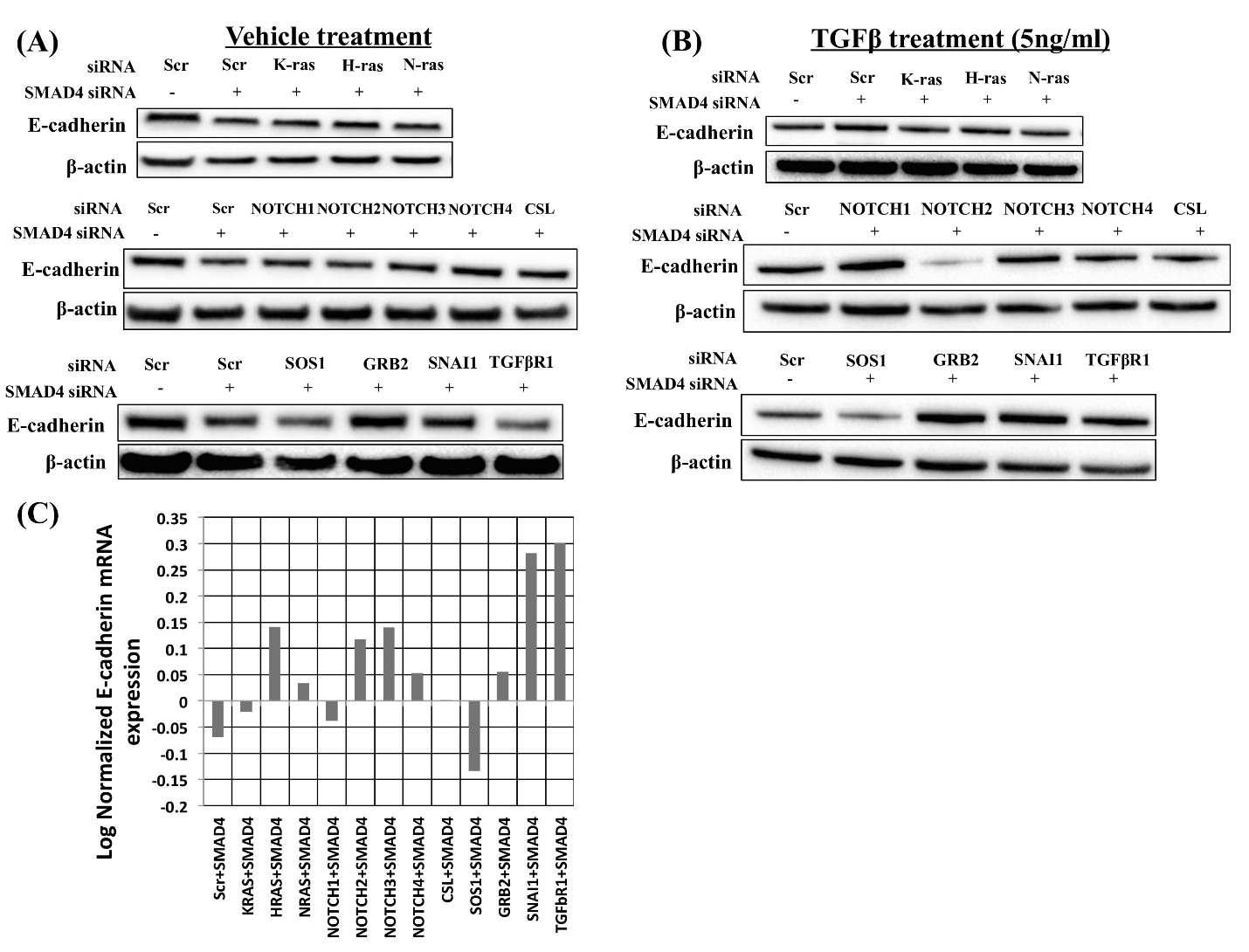
**

**Supplemental Figure 3: Western blots and mRNA expression used to quantify the effect of node knockdown combinations on E-cadherin expression.** A) The effect of node combinations on vehicle treated and B) TGFβ treated Huh7 cells. C) The effect of node combinations on E-cadherin mRNA expression by qRT-PCR in TGFβ treated versus vehicle treated Huh7 cells. 2 nM of each siRNA was used per transfection for a total of 4 nM siRNA when 2 siRNAs were used. 48 hours post transfection, cells were treated with either TGFβ (5ng/ml) or a vehicle control for 48 hours before harvesting.

**
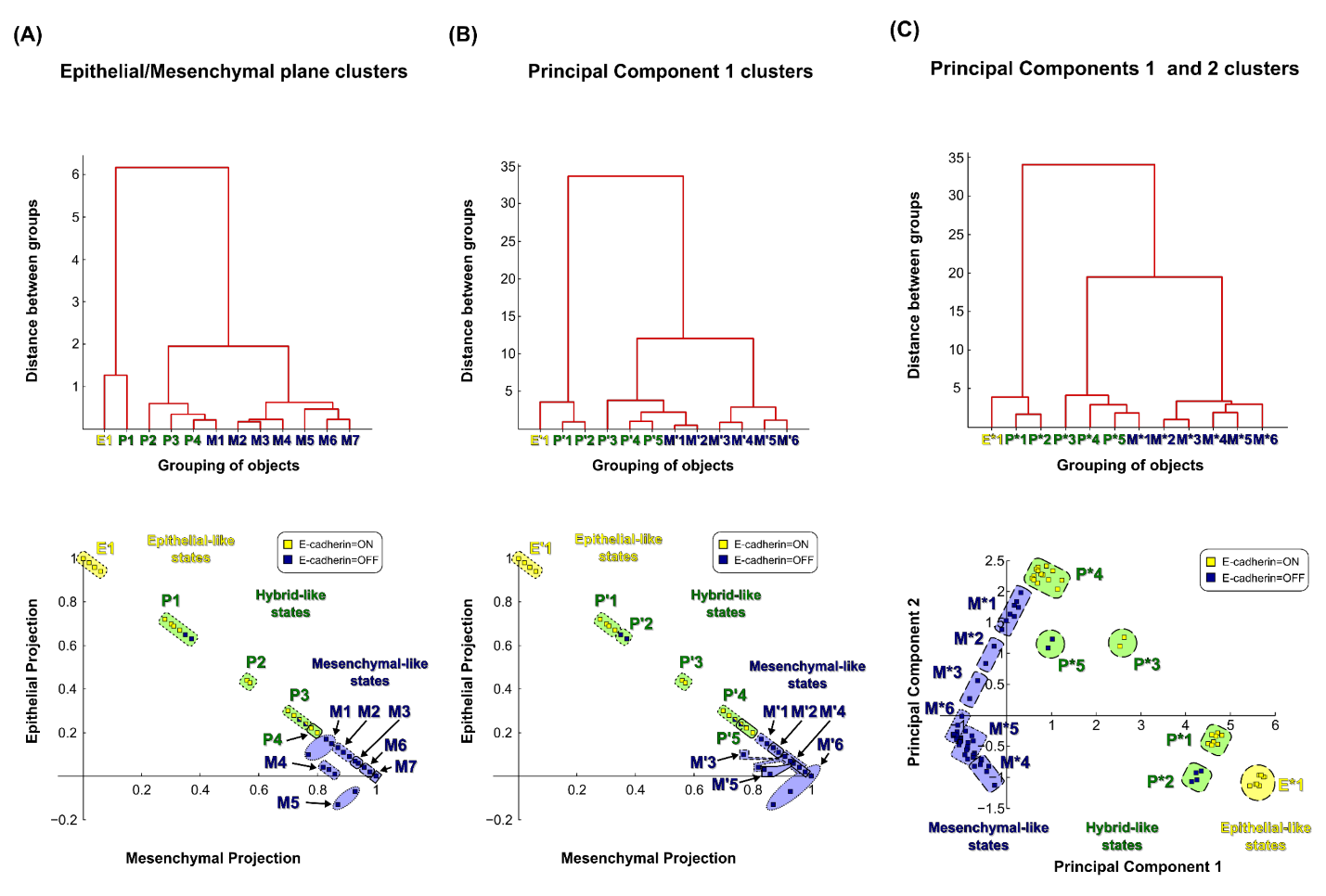
**

**Supplemental Figure 4: Hierarchical clustering of steady states in the single-node perturbed EMT networks.** Hierarchical clustering is obtained using the projection of the steady states on the epithelial/mesenchymal plane (panel A), the projection of the steady states on principal component 1 (panel B), and the projection of the steady states on principal components 1 and 2 (panel C). The dendrogram of the clusters (top) and the steady states that form the groups of the dendrogram (bottom) are shown. Each steady state in the bottom plots is represented by a square whose color denotes the presence (yellow) or absence (blue) of E-cadherin. The background color of the groups denotes the type of steady state (epithelial-like, yellow; hybrid-like, green; or mesenchymal-like, blue) and matches the background color in Figure 4C. In panel B, the groups of the principal component 1 dendrogram (top) are shown in the epithelial/mesenchymal plane (bottom). The position of principal component 1’s groups and clusters in the epithelial/mesenchymal plane shows the similarity between these two representations, which is further confirmed by the similarity between principal component 1’s groups and clusters and the ones obtained from the epithelial/mesenchymal plane, shown in panel A. A clear separation between the epithelial-like, mesenchymal-like and hybrid like states can also be seen in principal component plane (panel C, bottom). The principal component plane and the associated dendrogram (panel C, top) also show some additional structure among these subgroups.


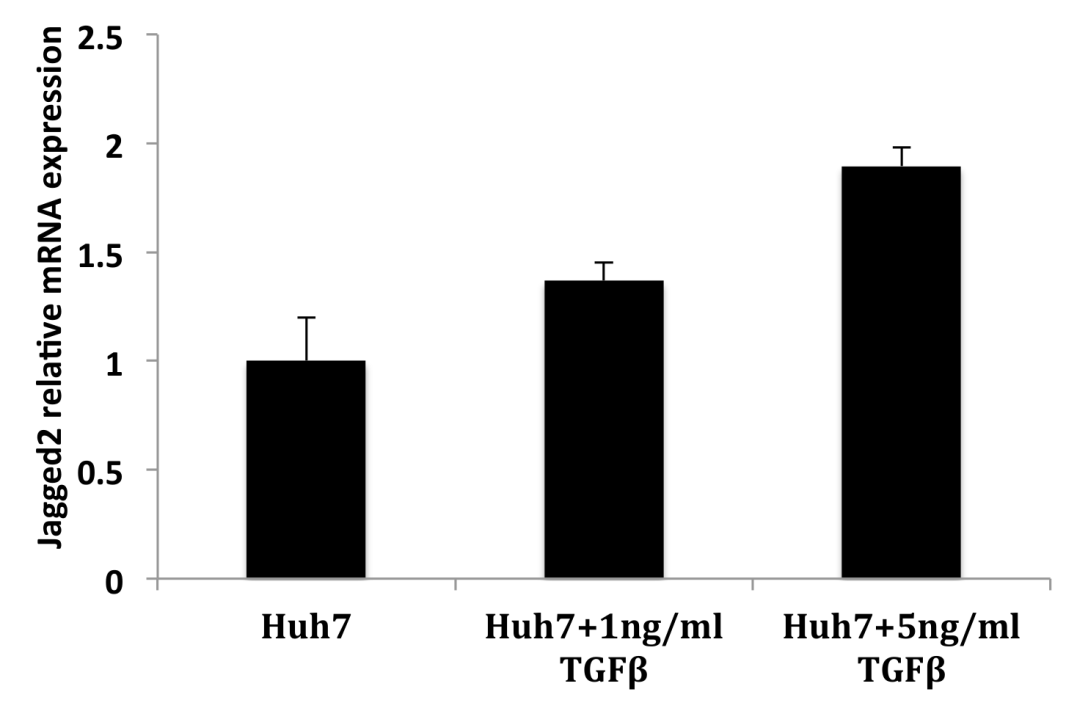


**Supplemental Figure 5: TGFβ treatment leads to increased Jagged2 Expression.** Treatment of Huh7 cells with TGFβ for 48 hours led to upregulation of Jagged2 mRNA expression as measured by qRT-PCR when compared to a vehicle control.

**
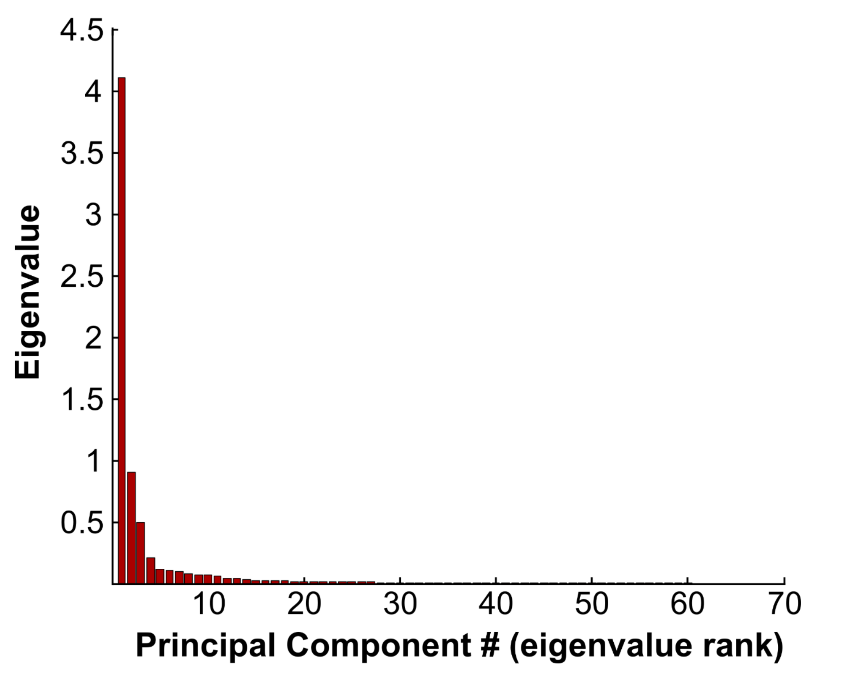
**

**Supplemental Figure 6: Eigenvalues associated to the principal components obtained by principal component analysis.** The eigenvalues associated with each principal component corresponds to the variance of the data in the direction of said principal component. The eigenvalues of principal components 1 and 2 are significantly larger than the rest of the eigenvalues, indicating that the first and second principal components capture the majority of the variance between the steady states and can be used to visualize their main differences.

**Supplemental Table 1: Boolean functions of the EMT network model.** The Boolean functions governing the dynamics of the EMT network model. For simplicity, the node states are represented by the node names. The Boolean rule of each node was constructed based on its upstream regulators in the EMT network (Fig. 1D), evidence regarding the conditionality among these regulators, and known biologic outcomes. The interested reader is referred to [3] for an explanation of the rules.

| **Node name** | **Boolean function** |
| --- | --- |
| AKT | ILK or PI3K |
| Dest_compl | (GSK3β and AXIN2 and β-catenin_nuc) or (GSK3β and Dest_compl) |
| AXIN2 | AXIN2 or TCF/LEF |
| β-catenin_memb | E-cadherin and not β-catenin_nuc |
| β-catenin_nuc | not Dest_compl and not β-catenin_memb and (not SUFU or not E-cadherin) |
| βTrCP | not Csn |
| CD44 | TCF/LEF |
| CDC42 | TGFβR or CHD1L |
| c-fos | ERK |
| cMet | HGF or CD44 |
| Csl | NOTCH_ic |
| Csn | NFκB |
| DELTA | RAS |
| DSH | Frizzled |
| E-cadherin | β-catenin_memb and (not SNAI1 or not HEY1 or not ZEB1 or not ZEB2 or not FOXC2 or not TWIST1 or not SNAI2) |
| EGFR | EGF |
| EGR1 | c-fos |
| EMT | not E-cadherin |
| ERK | MEK |
| FGFR | FGF |
| FOXC2 | Goosecoid or SNAI1 or TWIST1 |
| Frizzled | Wnt |
| FUS | SMO |
| GLI | TCF/LEF or not SUFU |
| GSK3β | not DSH and not AKT and (not Csn or not ERK or not Dest_compl) |
| HEY1 | Csl or SMAD |
| HIF1α | Hypoxia |
| IGF1R | IGF1 |
| IKKα | AKT |
| ILK | SMAD |
| Jagged | TCF/LEF or SMAD |
| LIV1 | STAT |
| LOXL23 | HIF1α |
| MEK | RAF or not RKIP |
| miR200 | not SNAI1 and not ZEB1 and not ZEB2 |
| NFκB | IKKα |
| NOTCH | DELTA or Jagged |
| NOTCH_ic | NOTCH |
| PAK1 | CDC42 |
| Patched | not SHH |
| PDGFR | PDGF |
| PI3K | RAS |
| RAF | RAS |
| RAS | SOS/GRB2 or SRC or not GSK3β or TCF/LEF |
| RKIP | not ERK or not SNAI1 |
| SHH | SMAD or GLI |
| SNAI2 | ERK or β-catenin_nuc or SNAI2 or TWIST1 |
| SMAD | (ERK or TGFβR) and (ZEB1 or not ZEB2) |
| SMO | not Patched |
| SNAI1 | GLI or LOXL23 or SMAD or LIV1 or PAK1 or Csl or EGR1 or Goosecoid or not βTrCP or not GSK3β |
| SOS/GRB2 | (PDGFR or cMet or TGFβR or FGFR or IGF1R or EGFR) and not ERK |
| SRC | PDGFR or EGFR or FGFR or cMet or IGF1R |
| STAT | SRC |
| SUFU | not FUS |
| TCF/LEF | β-catenin_nuc |
| TGFβ | Goosecoid or SNAI1 or TWIST1 or GLI |
| TGFβR | TGFβ |
| TWIST1 | NFκB or HIF1α or TCF/LEF or Goosecoid or SNAI1 |
| Wnt | GLI |
| ZEB1 | (HIF1α or SNAI1 or Goosecoid) and not miR200 |
| ZEB2 | (HIF1α or SNAI1 or Goosecoid) and not miR200 |

**Supplemental Table 2: Epithelial and mesenchymal steady states of the EMT network model.** The EMT network model has a group of steady states that match the epithelial or the mesenchymal phenotype and that we identify as the epithelial or the mesenchymal state, and whose node states are given in the table. The EMT network model has one steady state that can be identified as the mesenchymal state and 8 steady states that can be identified as epithelial states; these 8 steady states only differ in the state of three nodes that contain an activating self-loop (SNAI2, SUFU, and Dest_compl). We choose as the bona fide epithelial state the one that most closely matches the epithelial phenotype.

| **Node name** | **Epithelial node state** | **Mesenchymal node state** |
| --- | --- | --- |
| AKT | OFF | ON |
| Dest_compl | ON | OFF |
| AXIN2 | OFF | ON |
| β-catenin_memb | ON | OFF |
| β-catenin_nuc | OFF | ON |
| βTrCP | ON | OFF |
| CD44 | OFF | ON |
| CDC42 | OFF | ON |
| c-fos | OFF | ON |
| cMet | OFF | ON |
| Csl | OFF | ON |
| Csn | OFF | ON |
| DELTA | OFF | ON |
| DSH | OFF | ON |
| E-cadherin | ON | OFF |
| EGFR | OFF | OFF |
| EGR1 | OFF | ON |
| EMT | OFF | ON |
| ERK | OFF | ON |
| FGFR | OFF | OFF |
| FOXC2 | OFF | ON |
| Frizzled | OFF | ON |
| FUS | OFF | ON |
| GLI | OFF | ON |
| GSK3β | ON | OFF |
| HEY1 | OFF | ON |
| HIF1α | OFF | OFF |
| IGF1R | OFF | OFF |
| IKKα | OFF | ON |
| ILK | OFF | ON |
| Jagged | OFF | ON |
| LIV1 | OFF | ON |
| LOXL23 | OFF | OFF |
| MEK | OFF | ON |
| miR200 | ON | OFF |
| NFκB | OFF | ON |
| NOTCH | OFF | ON |
| NOTCH_ic | OFF | ON |
| PAK1 | OFF | ON |
| Patched | ON | OFF |
| PDGFR | OFF | OFF |
| PI3K | OFF | ON |
| RAF | OFF | ON |
| RAS | OFF | ON |
| RKIP | ON | OFF |
| SHH | OFF | ON |
| SNAI2 | OFF | ON |
| SMAD | OFF | ON |
| SMO | OFF | ON |
| SNAI1 | OFF | ON |
| SOS/GRB2 | OFF | OFF |
| SRC | OFF | ON |
| STAT | OFF | ON |
| SUFU | ON | OFF |
| TCF/LEF | OFF | ON |
| TGFβ | OFF | ON |
| TGFβR | OFF | ON |
| TWIST1 | OFF | ON |
| Wnt | OFF | ON |
| ZEB1 | OFF | ON |
| ZEB2 | OFF | ON |
| EGF | OFF | OFF |
| FGF | OFF | OFF |
| HGF | OFF | OFF |
| Goosecoid | OFF | OFF |
| Hypoxia | OFF | OFF |
| IGF1 | OFF | OFF |
| PDGF | OFF | OFF |
| CHD1L | OFF | OFF |

**Supplemental Table 3: Summary of the epithelial control sets.**  Each column contains nodes that are part of only one of the five groups of nodes with a yellow background in Figure 1D. Group A, B, and C is the top left, top middle, and top right group of nodes on Figure 1D, respectively, group D is the central group of nodes, and group E is the bottom left group of nodes. A complete control set requires one element from each of the five groups. Specifically, a control set is obtained by taking any one element from each column in a given row. A “~” signifies that a node be in the OFF state. Otherwise, a node is in the ON state. For example, a possible control set listed in the first row is {SHH=OFF, RAS=OFF, TGFβR=OFF, MEK=OFF, β-catenin_memb=ON}, while a possible control set from the last row is {GLI=OFF, GSK3β=ON, TWIST=OFF, SNAI1=OFF, β-catenin_memb=ON}. There is a total of 6x2x2 + 6x2 + 6x2x2 + 5x3x2 + 5x2x2 + 2 + 3 =115 epithelial control sets.

| **Group A** | **Group B** | **Group C** | **Group D** | **Group E** |
| --- | --- | --- | --- | --- |
| ~SHH, ~FUS, ~SMO, SUFU, PTCH, ~GLI | ~RAS | ~TGFβR | ~MEK, ~ERK | E-cadherin, β-catenin_memb |
| ~SHH, ~FUS, ~SMO, SUFU, PTCH, ~GLI | ~RAS | ~SMAD | ~SNAI1 | E-cadherin, β-catenin_memb |
| ~SHH, ~FUS, ~SMO, SUFU, PTCH, ~GLI | GSK3β | ~TGFβR | ~MEK, ~ERK | Ecadherin, β-catenin_memb |
| ~SHH, ~FUS, ~SMO, SUFU, PTCH | GSK3β | ~TGFβR, ~TGFβ, ~TWIST | ~SNAI1 | E-cadherin, β-catenin_memb |
| ~SHH, ~FUS, ~SMO, SUFU, PTCH | GSK3β | ~TGFβR, ~TGFβ | RKIP | E-cadherin β-catenin_memb |
| ~GLI | GSK3β | ~TGFβR, ~TGFβ | RKIP | β-catenin_memb |
| ~GLI | GSK3β | ~TGFβR, ~TGFβ, ~TWIST | ~SNAI1 | β-catenin_memb |

**Supplemental Table 4: Single node knockouts that suppress EMT.** We systematically explore how knocking out each separate node (except for TGFβ, TGFβR, E-cadherin, and EMT) affects EMT. Starting from the epithelial steady state, we fix TGFβ=ON and the node of interest to OFF, update the system for over 10,000 steps, and check the state of the EMT node after the last update. Every node knockout is repeated for
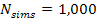
 simulations. The EMT % is given by the average number of simulations at the end of which the EMT node was found in the ON state. The EMT % is accurate to the first or second digit of the percentage, as measured by the standard deviation of the mean of the estimated EMT percentage,
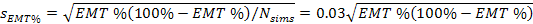
 (e.g., if EMT %
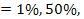
or
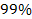
 then the standard deviation of the mean are
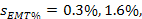
 or
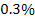
, respectively). The nodes not shown in the table do not have any effect on the EMT %, i.e., they result in an EMT % = 100 %.

| **Node** | **% EMT**  **(n=1000 simulations)** |
| --- | --- |
| FOXC2 | 0.0 |
| SNAI1 | 0.0 |
| HEY1 | 0.0 |
| ZEB2 | 0.0 |
| SNAI2 | 0.0 |
| ZEB1 | 0.0 |
| TWIST1 | 0.0 |
| SMAD | 96.2 |

**Supplemental Table 5: Double node knockouts that suppress EMT.** We systematically explore how knocking out combinations of two nodes affects EMT. Starting from the epithelial steady state, we fix TGFβ=ON and the nodes of interest to OFF, update the system for over 10,000 steps, and check the state of the EMT node after the last update. Every knockout combination is repeated for
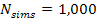
 simulations. The EMT % is accurate to the first or second digit of the percentage, as measured by the standard deviation of the mean of the estimated EMT percentage,
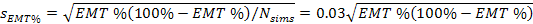
 (e.g., if EMT %
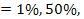
or
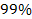
 then the standard deviation of the mean are
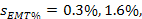
 or
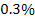
, respectively). The single nodes whose knockout fully suppressed EMT in Supplemental Table 4, as well as TGFβ, TGFβR, E-cadherin, and EMT, are not included in this analysis. All double node knockouts that suppress the EMT % include SMAD as one of the nodes in the combination. Only knockouts that have an EMT % < 90% are shown in the table.

| **Node 1** | **Node 2** | **% EMT**  **(n=1000 simulations)** |
| --- | --- | --- |
| Csl | SMAD | 0.0 |
| SOS_GRB2 | SMAD | 0.0 |
| DELTA | SMAD | 0.0 |
| NOTCH | SMAD | 0.0 |
| RAS | SMAD | 0.0 |
| NOTCH_ic | SMAD | 0.0 |
| RKIP | SMAD | 37.7 |
| AKT | SMAD | 34.5 |
| PI3K | SMAD | 34.9 |

**Supplemental Table 6: Triple node knockouts that suppress EMT**. We systematically explore how knocking out combinations of three nodes affects EMT. Starting from the epithelial steady state, we fix TGFβ=ON and the nodes of interest to OFF, update the system for over 10,000 steps, and check the state of the EMT node after the last update. Every knockout combination is repeated for
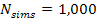
 simulations. The EMT % is accurate to the first or second digit of the percentage, as measured by the standard deviation of the mean of the estimated EMT percentage,
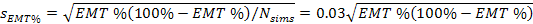
 (e.g., if EMT %
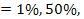
or
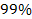
 then the standard deviation of the mean are
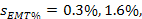
 or
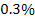
, respectively). The single nodes and double node combinations whose knockout fully suppressed EMT in Supplemental Tables 4 and 5, respectively, are not included in this analysis. All triple node knockouts that suppress the EMT % include SMAD as one of the nodes in the combination. Only knockouts that have an EMT % < 90% are shown in the table.

| **Node 1** | **Node 2** | **Node 3** | **% EMT**  **(n=1000 simulations)** |
| --- | --- | --- | --- |
| SMAD | PI3K | RKIP | 5.1 |
| SMAD | AKT | RKIP | 5.4 |
| SMAD | AKT | ILK | 30.5 |
| SMAD | AKT | SMO | 30.8 |
| SMAD | AKT | GLI | 31.2 |
| SMAD | PI3K | LOXL23 | 31.3 |
| SMAD | PI3K | IKKα | 31.3 |
| SMAD | PI3K | Frizzled | 31.5 |
| SMAD | PI3K | Dest_compl | 31.7 |
| SMAD | AKT | Frizzled | 31.7 |
| SMAD | AKT | Csn | 31.7 |
| SMAD | AKT | Dest_compl | 32 |
| SMAD | PI3K | CHD1L | 32.3 |
| SMAD | AKT | NfκB | 32.3 |
| SMAD | PI3K | CD44 | 32.4 |
| SMAD | PI3K | SRC | 32.4 |
| SMAD | AKT | IKKα | 32.4 |
| SMAD | PI3K | Jagged | 32.5 |
| SMAD | AKT | β-catenin_nuc | 32.6 |
| SMAD | AKT | cfos | 32.6 |
| SMAD | PI3K | SMO | 32.6 |
| SMAD | PI3K | LIV1 | 32.7 |
| SMAD | AKT | Wnt | 32.7 |
| SMAD | PI3K | ILK | 32.7 |
| SMAD | AKT | cMet | 32.8 |
| SMAD | AKT | FUS | 32.8 |
| SMAD | AKT | TCF/LEF | 32.9 |
| SMAD | PI3K | Wnt | 32.9 |
| SMAD | PI3K | SHH | 33 |
| SMAD | PI3K | cMet | 33.1 |
| SMAD | AKT | SRC | 33.1 |
| SMAD | PI3K | GLI | 33.3 |
| SMAD | PI3K | cfos | 33.4 |
| SMAD | PI3K | TCF/LEF | 33.6 |
| SMAD | PI3K | HIF1α | 33.7 |
| SMAD | AKT | STAT | 33.7 |
| SMAD | AKT | HIF1α | 33.8 |
| SMAD | PI3K | DSH | 33.8 |
| SMAD | PI3K | β-catenin_nuc | 33.9 |
| SMAD | PI3K | AKT | 33.9 |
| SMAD | AKT | AXIN2 | 34.2 |
| SMAD | PI3K | NfκB | 34.4 |
| SMAD | AKT | Jagged | 34.4 |
| SMAD | RKIP | FUS | 34.4 |
| SMAD | AKT | CHD1L | 34.6 |
| SMAD | AKT | LIV1 | 34.6 |
| SMAD | RKIP | CD44 | 34.7 |
| SMAD | AKT | EGR1 | 34.9 |
| SMAD | PI3K | FUS | 34.9 |
| SMAD | AKT | LOXL23 | 35.1 |
| SMAD | AKT | SHH | 35.1 |
| SMAD | PI3K | STAT | 35.2 |
| SMAD | RKIP | SMO | 35.3 |
| SMAD | RKIP | SHH | 35.3 |
| SMAD | PI3K | AXIN2 | 35.5 |
| SMAD | PI3K | EGR1 | 35.5 |
| SMAD | PI3K | Csn | 35.5 |
| SMAD | RKIP | IKKα | 35.8 |
| SMAD | AKT | DSH | 35.8 |
| SMAD | RKIP | CHD1L | 36.1 |
| SMAD | RKIP | LIV1 | 36.1 |
| SMAD | AKT | CD44 | 36.2 |
| SMAD | RKIP | Dest_compl | 36.5 |
| SMAD | RKIP | HIF1α | 36.6 |
| SMAD | RKIP | ILK | 36.6 |
| SMAD | RKIP | Wnt | 36.7 |
| SMAD | AKT | PAK1 | 36.8 |
| SMAD | RKIP | SRC | 37 |
| SMAD | PI3K | βTrCP | 37.1 |
| SMAD | RKIP | CDC42 | 37.4 |
| SMAD | RKIP | Frizzled | 37.5 |
| SMAD | RKIP | EGR1 | 37.5 |
| SMAD | RKIP | STAT | 37.6 |
| SMAD | AKT | βTrCP | 37.6 |
| SMAD | RKIP | β-catenin_nuc | 37.8 |
| SMAD | RKIP | TCF/LEF | 37.8 |
| SMAD | RKIP | NfκB | 38 |
| SMAD | PI3K | CDC42 | 38.2 |
| SMAD | RKIP | cMet | 38.6 |
| SMAD | RKIP | cfos | 38.6 |
| SMAD | RKIP | PAK1 | 38.7 |
| SMAD | RKIP | GLI | 38.7 |
| SMAD | AKT | miR200 | 38.7 |
| SMAD | PI3K | miR200 | 38.8 |
| SMAD | RKIP | miR200 | 38.9 |
| SMAD | RKIP | LOXL23 | 39 |
| SMAD | RKIP | RAF | 39 |
| SMAD | PI3K | PAK1 | 39.2 |
| SMAD | RKIP | Csn | 39.2 |
| SMAD | AKT | CDC42 | 39.3 |
| SMAD | RKIP | DSH | 39.8 |
| SMAD | RKIP | Jagged | 40 |
| SMAD | RKIP | AXIN2 | 40 |
| SMAD | RKIP | βTrCP | 41 |

**Supplemental Table 7: Quadruple node knockouts that suppress EMT.** We systematically explore how knocking out combinations of four nodes affects EMT. Starting from the epithelial steady state, we fix TGFβ=ON and the nodes of interest to OFF, update the system for over 10,000 steps, and check the state of the EMT node after the last update. Every knockout combination is repeated for
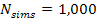
 simulations. The EMT % is accurate to the first or second digit of the percentage, as measured by the standard deviation of the mean of the estimated EMT percentage,
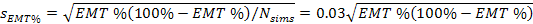
 (e.g., if EMT %
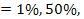
or
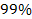
 then the standard deviation of the mean are
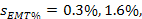
 or
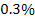
, respectively). The single nodes and double node combinations whose knockout fully suppressed EMT in Supplemental Tables 4 and 5, respectively, are not included in this analysis. All quadruple node knockouts that suppress the EMT % include SMAD as one of the nodes in the combination. Only knockouts that have an EMT % < 90% are shown in the table.

| **Node 1** | **Node 2** | **Node 3** | **Node 4** | **% EMT**  **(n=1000 simulations)** |
| --- | --- | --- | --- | --- |
| SMAD | PI3K | RKIP | PAK1 | 2.7 |
| SMAD | AKT | PAK1 | RKIP | 2.7 |
| SMAD | AKT | RKIP | CDC42 | 2.8 |
| SMAD | PI3K | RKIP | RKIP | 3.2 |
| SMAD | PI3K | RKIP | RKIP | 3.6 |
| SMAD | PI3K | RKIP | EGR1 | 3.9 |
| SMAD | PI3K | RKIP | RKIP | 4 |
| SMAD | PI3K | RKIP | Csn | 4 |
| SMAD | AKT | EGR1 | RKIP | 4.2 |
| SMAD | AKT | RKIP | SMO | 4.2 |
| SMAD | PI3K | RKIP | SHH | 4.2 |
| SMAD | AKT | RKIP | Csn | 4.3 |
| SMAD | PI3K | RKIP | RKIP | 4.5 |
| SMAD | PI3K | RKIP | c-fos | 4.5 |
| SMAD | AKT | STAT | RKIP | 4.5 |
| SMAD | AKT | RKIP | CHD1L | 4.7 |
| SMAD | PI3K | RKIP | RKIP | 4.8 |
| SMAD | PI3K | RKIP | cMet | 4.8 |
| SMAD | PI3K | RKIP | GLI | 4.8 |
| SMAD | AKT | LIV1 | RKIP | 4.8 |
| SMAD | PI3K | RKIP | RKIP | 5 |
| SMAD | PI3K | RKIP | RKIP | 5 |
| SMAD | AKT | RKIP | AXIN2 | 5 |
| SMAD | AKT | RKIP | IKKα | 5 |
| SMAD | PI3K | RKIP | ILK | 5 |
| SMAD | PI3K | RKIP | LIV1 | 5.1 |
| SMAD | PI3K | RKIP | SRC | 5.1 |
| SMAD | PI3K | RKIP | AKT | 5.2 |
| SMAD | AKT | SRC | RKIP | 5.2 |
| SMAD | AKT | CD44 | RKIP | 5.4 |
| SMAD | AKT | GLI | RKIP | 5.4 |
| SMAD | PI3K | RKIP | RKIP | 5.5 |
| SMAD | AKT | c-fos | RKIP | 5.5 |
| SMAD | PI3K | RKIP | FUS | 5.5 |
| SMAD | AKT | RKIP | FUS | 5.5 |
| SMAD | PI3K | RKIP | RKIP | 5.6 |
| SMAD | AKT | RKIP | β-catenin_nuc | 5.6 |
| SMAD | AKT | RKIP | Wnt | 5.6 |
| SMAD | AKT | RKIP | ILK | 5.6 |
| SMAD | AKT | RKIP | LOXL23 | 5.7 |
| SMAD | PI3K | RKIP | DSH | 5.7 |
| SMAD | PI3K | RKIP | CD44 | 5.8 |
| SMAD | AKT | RKIP | Jagged | 5.8 |
| SMAD | AKT | RKIP | HIF1α | 5.8 |
| SMAD | AKT | RKIP | DSH | 5.8 |
| SMAD | PI3K | RKIP | RKIP | 5.9 |
| SMAD | PI3K | RKIP | STAT | 5.9 |
| SMAD | AKT | RKIP | Frizzled | 5.9 |
| SMAD | PI3K | RKIP | RKIP | 6 |
| SMAD | AKT | RKIP | NfκB | 6 |
| SMAD | AKT | RKIP | TCF/LEF | 6.1 |
| SMAD | AKT | cMet | RKIP | 6.3 |
| SMAD | AKT | RKIP | SHH | 6.3 |
| SMAD | AKT | RKIP | Dest_compl | 6.4 |
| SMAD | PI3K | RKIP | SMO | 6.6 |
| SMAD | PI3K | RKIP | RKIP | 7 |
| SMAD | AKT | RKIP | miR200 | 7.2 |
| SMAD | PI3K | RKIP | Wnt | 7.3 |
| SMAD | PI3K | RKIP | RKIP | 7.4 |
| SMAD | AKT | RKIP | βTrCP | 7.6 |
| SMAD | PI3K | RKIP | RAF | 7.9 |
| SMAD | AKT | RKIP | RAF | 8.1 |
| SMAD | PI3K | RKIP | RKIP | 9.5 |
| SMAD | AKT | RKIP | DSH | 28.7 |
| SMAD | AKT | RKIP | FUS | 29.4 |
| SMAD | PI3K | RKIP | FUS | 29.7 |
| SMAD | AKT | RKIP | SHH | 29.9 |
| SMAD | AKT | RKIP | DSH | 29.9 |
| SMAD | PI3K | RKIP | DSH | 30 |
| SMAD | AKT | cMet | Jagged | 30.1 |
| SMAD | PI3K | RKIP | ILK | 30.1 |
| SMAD | PI3K | RKIP | LIV1 | 30.4 |
| SMAD | PI3K | RKIP | IKKα | 30.5 |
| SMAD | PI3K | RKIP | Csn | 30.5 |
| SMAD | AKT | RKIP | Csn | 30.5 |
| SMAD | PI3K | RKIP | DSH | 30.5 |
| SMAD | PI3K | RKIP | IKKα | 30.6 |
| SMAD | AKT | Jagged | Frizzled | 30.6 |
| SMAD | AKT | NfκB | Dest_compl | 30.6 |
| SMAD | AKT | EGR1 | IKKα | 30.6 |
| SMAD | AKT | RKIP | SMO | 30.6 |
| SMAD | AKT | CD44 | β-catenin_nuc | 30.7 |
| SMAD | AKT | c-fos | LOXL23 | 30.7 |
| SMAD | AKT | RKIP | SMO | 30.7 |
| SMAD | PI3K | RKIP | ILK | 30.7 |
| SMAD | PI3K | RKIP | SHH | 30.7 |
| SMAD | PI3K | RKIP | SHH | 30.7 |
| SMAD | AKT | RKIP | SHH | 30.7 |
| SMAD | PI3K | AXIN2 | Dest_compl | 30.8 |
| SMAD | PI3K | RKIP | LIV1 | 30.8 |
| SMAD | AKT | LIV1 | NfκB | 30.8 |
| SMAD | AKT | SRC | CD44 | 30.8 |
| SMAD | AKT | RKIP | Csn | 30.8 |
| SMAD | PI3K | RKIP | SHH | 30.8 |
| SMAD | PI3K | RKIP | DSH | 30.8 |
| SMAD | PI3K | RKIP | EGR1 | 30.9 |
| SMAD | AKT | CHD1L | Jagged | 30.9 |
| SMAD | AKT | LIV1 | EGR1 | 30.9 |
| SMAD | AKT | STAT | EGR1 | 30.9 |
| SMAD | PI3K | RKIP | DSH | 30.9 |
| SMAD | PI3K | CHD1L | TCF/LEF | 31 |
| SMAD | AKT | CHD1L | TCF/LEF | 31 |
| SMAD | PI3K | RKIP | Wnt | 31 |
| SMAD | PI3K | RKIP | CD44 | 31.1 |
| SMAD | AKT | LIV1 | Frizzled | 31.1 |
| SMAD | AKT | CD44 | Jagged | 31.1 |
| SMAD | PI3K | RKIP | ILK | 31.1 |
| SMAD | PI3K | RKIP | SHH | 31.1 |
| SMAD | AKT | NfκB | AXIN2 | 31.2 |
| SMAD | AKT | LIV1 | AXIN2 | 31.2 |
| SMAD | AKT | c-fos | β-catenin_nuc | 31.2 |
| SMAD | AKT | RKIP | SMO | 31.2 |
| SMAD | AKT | RKIP | SMO | 31.2 |
| SMAD | PI3K | RKIP | ILK | 31.2 |
| SMAD | AKT | RKIP | SHH | 31.2 |
| SMAD | AKT | HIF1α | Frizzled | 31.3 |
| SMAD | PI3K | RKIP | FUS | 31.3 |
| SMAD | AKT | RKIP | FUS | 31.3 |
| SMAD | AKT | RKIP | FUS | 31.3 |
| SMAD | PI3K | RKIP | SMO | 31.3 |
| SMAD | PI3K | HIF1α | Jagged | 31.4 |
| SMAD | PI3K | RKIP | CD44 | 31.4 |
| SMAD | PI3K | RKIP | CD44 | 31.4 |
| SMAD | PI3K | RKIP | cMet | 31.4 |
| SMAD | PI3K | RKIP | c-fos | 31.4 |
| SMAD | PI3K | RKIP | GLI | 31.4 |
| SMAD | PI3K | RKIP | SRC | 31.4 |
| SMAD | AKT | LOXL23 | TCF/LEF | 31.4 |
| SMAD | AKT | GLI | AXIN2 | 31.4 |
| SMAD | PI3K | RKIP | GLI | 31.5 |
| SMAD | AKT | cMet | HIF1α | 31.5 |
| SMAD | AKT | GLI | LIV1 | 31.5 |
| SMAD | AKT | RKIP | Csn | 31.5 |
| SMAD | RKIP | Wnt | CD44 | 31.5 |
| SMAD | PI3K | RKIP | SHH | 31.5 |
| SMAD | PI3K | TCF/LEF | Frizzled | 31.6 |
| SMAD | PI3K | LOXL23 | Dest_compl | 31.6 |
| SMAD | PI3K | RKIP | LIV1 | 31.6 |
| SMAD | AKT | LIV1 | β-catenin_nuc | 31.6 |
| SMAD | AKT | CD44 | NfκB | 31.6 |
| SMAD | AKT | c-fos | EGR1 | 31.6 |
| SMAD | PI3K | RKIP | SMO | 31.6 |
| SMAD | PI3K | LOXL23 | β-catenin_nuc | 31.7 |
| SMAD | PI3K | HIF1α | AXIN2 | 31.7 |
| SMAD | PI3K | RKIP | GLI | 31.7 |
| SMAD | AKT | IKKα | TCF/LEF | 31.7 |
| SMAD | AKT | CD44 | EGR1 | 31.7 |
| SMAD | PI3K | RKIP | FUS | 31.7 |
| SMAD | PI3K | RKIP | FUS | 31.7 |
| SMAD | AKT | RKIP | ILK | 31.7 |
| SMAD | AKT | RKIP | ILK | 31.7 |
| SMAD | PI3K | RKIP | NfκB | 31.8 |
| SMAD | PI3K | RKIP | LIV1 | 31.8 |
| SMAD | PI3K | RKIP | cMet | 31.8 |
| SMAD | AKT | Jagged | Dest_compl | 31.8 |
| SMAD | AKT | EGR1 | NfκB | 31.8 |
| SMAD | AKT | CD44 | LOXL23 | 31.8 |
| SMAD | AKT | cMet | β-catenin_nuc | 31.8 |
| SMAD | AKT | SRC | Jagged | 31.8 |
| SMAD | PI3K | RKIP | Csn | 31.8 |
| SMAD | AKT | RKIP | Csn | 31.8 |
| SMAD | PI3K | RKIP | Wnt | 31.8 |
| SMAD | AKT | RKIP | ILK | 31.8 |
| SMAD | AKT | RKIP | SHH | 31.8 |
| SMAD | PI3K | RKIP | EGR1 | 31.9 |
| SMAD | PI3K | RKIP | c-fos | 31.9 |
| SMAD | PI3K | RKIP | c-fos | 31.9 |
| SMAD | PI3K | RKIP | c-fos | 31.9 |
| SMAD | PI3K | RKIP | GLI | 31.9 |
| SMAD | AKT | Jagged | TCF/LEF | 31.9 |
| SMAD | AKT | CHD1L | β-catenin_nuc | 31.9 |
| SMAD | AKT | IKKα | β-catenin_nuc | 31.9 |
| SMAD | AKT | CD44 | TCF/LEF | 31.9 |
| SMAD | AKT | c-fos | CHD1L | 31.9 |
| SMAD | AKT | GLI | Dest_compl | 31.9 |
| SMAD | PI3K | RKIP | Csn | 31.9 |
| SMAD | PI3K | RKIP | Csn | 31.9 |
| SMAD | AKT | RKIP | SMO | 31.9 |
| SMAD | AKT | RKIP | SMO | 31.9 |
| SMAD | AKT | RKIP | Wnt | 31.9 |
| SMAD | PI3K | RKIP | SHH | 31.9 |
| SMAD | PI3K | RKIP | DSH | 31.9 |
| SMAD | AKT | RKIP | DSH | 31.9 |
| SMAD | PI3K | RKIP | LIV1 | 32 |
| SMAD | PI3K | RKIP | cMet | 32 |
| SMAD | PI3K | RKIP | c-fos | 32 |
| SMAD | PI3K | RKIP | GLI | 32 |
| SMAD | PI3K | RKIP | AKT | 32 |
| SMAD | AKT | STAT | LIV1 | 32 |
| SMAD | PI3K | RKIP | SMO | 32 |
| SMAD | AKT | RKIP | Wnt | 32 |
| SMAD | PI3K | RKIP | ILK | 32 |
| SMAD | PI3K | RKIP | ILK | 32 |
| SMAD | PI3K | RKIP | ILK | 32 |
| SMAD | AKT | RKIP | DSH | 32 |
| SMAD | PI3K | RKIP | LIV1 | 32.1 |
| SMAD | PI3K | RKIP | STAT | 32.1 |
| SMAD | PI3K | RKIP | AKT | 32.1 |
| SMAD | AKT | NfκB | CHD1L | 32.1 |
| SMAD | AKT | EGR1 | Dest_compl | 32.1 |
| SMAD | AKT | LIV1 | CHD1L | 32.1 |
| SMAD | AKT | SRC | Dest_compl | 32.1 |
| SMAD | AKT | SRC | TCF/LEF | 32.1 |
| SMAD | AKT | RKIP | Wnt | 32.1 |
| SMAD | AKT | RKIP | Wnt | 32.1 |
| SMAD | PI3K | RKIP | ILK | 32.1 |
| SMAD | PI3K | RKIP | DSH | 32.1 |
| SMAD | AKT | RKIP | DSH | 32.1 |
| SMAD | PI3K | Jagged | β-catenin_nuc | 32.2 |
| SMAD | PI3K | HIF1α | LOXL23 | 32.2 |
| SMAD | PI3K | RKIP | EGR1 | 32.2 |
| SMAD | PI3K | RKIP | SRC | 32.2 |
| SMAD | AKT | GLI | TCF/LEF | 32.2 |
| SMAD | AKT | SRC | c-fos | 32.2 |
| SMAD | PI3K | RKIP | FUS | 32.2 |
| SMAD | PI3K | RKIP | SMO | 32.2 |
| SMAD | AKT | RKIP | SMO | 32.2 |
| SMAD | PI3K | RKIP | Wnt | 32.2 |
| SMAD | AKT | RKIP | DSH | 32.2 |
| SMAD | PI3K | Jagged | LOXL23 | 32.3 |
| SMAD | PI3K | RKIP | IKKα | 32.3 |
| SMAD | PI3K | RKIP | LIV1 | 32.3 |
| SMAD | PI3K | RKIP | GLI | 32.3 |
| SMAD | AKT | IKKα | Dest_compl | 32.3 |
| SMAD | AKT | GLI | SUFU | 32.3 |
| SMAD | AKT | GLI | NfκB | 32.3 |
| SMAD | AKT | RKIP | SMO | 32.3 |
| SMAD | AKT | RKIP | SMO | 32.3 |
| SMAD | PI3K | RKIP | SMO | 32.3 |
| SMAD | PI3K | RKIP | Wnt | 32.3 |
| SMAD | PI3K | RKIP | DSH | 32.3 |
| SMAD | PI3K | RKIP | DSH | 32.3 |
| SMAD | AKT | RKIP | DSH | 32.3 |
| SMAD | AKT | RKIP | DSH | 32.3 |
| SMAD | PI3K | Frizzled | Dest_compl | 32.4 |
| SMAD | PI3K | RKIP | GLI | 32.4 |
| SMAD | AKT | HIF1α | β-catenin_nuc | 32.4 |
| SMAD | AKT | LIV1 | LOXL23 | 32.4 |
| SMAD | AKT | SRC | Frizzled | 32.4 |
| SMAD | PI3K | RKIP | Csn | 32.4 |
| SMAD | AKT | RKIP | Csn | 32.4 |
| SMAD | AKT | RKIP | Csn | 32.4 |
| SMAD | AKT | RKIP | SMO | 32.4 |
| SMAD | PI3K | RKIP | Wnt | 32.4 |
| SMAD | PI3K | RKIP | ILK | 32.4 |
| SMAD | AKT | RKIP | SHH | 32.4 |
| SMAD | PI3K | RKIP | DSH | 32.4 |
| SMAD | AKT | RKIP | DSH | 32.4 |
| SMAD | PI3K | RKIP | NfκB | 32.5 |
| SMAD | PI3K | RKIP | STAT | 32.5 |
| SMAD | PI3K | RKIP | AKT | 32.5 |
| SMAD | PI3K | RKIP | Csn | 32.5 |
| SMAD | PI3K | RKIP | Csn | 32.5 |
| SMAD | AKT | RKIP | Csn | 32.5 |
| SMAD | AKT | RKIP | FUS | 32.5 |
| SMAD | AKT | RKIP | FUS | 32.5 |
| SMAD | AKT | RKIP | FUS | 32.5 |
| SMAD | PI3K | RKIP | Wnt | 32.5 |
| SMAD | AKT | RKIP | SHH | 32.5 |
| SMAD | PI3K | RKIP | SHH | 32.5 |
| SMAD | PI3K | RKIP | DSH | 32.5 |
| SMAD | PI3K | RKIP | DSH | 32.5 |
| SMAD | AKT | RKIP | DSH | 32.5 |
| SMAD | PI3K | RKIP | cMet | 32.6 |
| SMAD | PI3K | RKIP | cMet | 32.6 |
| SMAD | AKT | CHD1L | AXIN2 | 32.6 |
| SMAD | PI3K | RKIP | AKT | 32.6 |
| SMAD | AKT | CD44 | AXIN2 | 32.6 |
| SMAD | AKT | SRC | STAT | 32.6 |
| SMAD | PI3K | RKIP | FUS | 32.6 |
| SMAD | RKIP | FUS | LOXL23 | 32.6 |
| SMAD | PI3K | RKIP | FUS | 32.6 |
| SMAD | AKT | RKIP | FUS | 32.6 |
| SMAD | AKT | RKIP | SMO | 32.6 |
| SMAD | AKT | RKIP | Wnt | 32.6 |
| SMAD | PI3K | CHD1L | β-catenin_nuc | 32.7 |
| SMAD | PI3K | CHD1L | Frizzled | 32.7 |
| SMAD | PI3K | RKIP | CD44 | 32.7 |
| SMAD | AKT | TCF/LEF | Frizzled | 32.7 |
| SMAD | AKT | HIF1α | Dest_compl | 32.7 |
| SMAD | AKT | STAT | CD44 | 32.7 |
| SMAD | AKT | GLI | IKKα | 32.7 |
| SMAD | AKT | GLI | EGR1 | 32.7 |
| SMAD | AKT | RKIP | Csn | 32.7 |
| SMAD | AKT | RKIP | FUS | 32.7 |
| SMAD | AKT | RKIP | Wnt | 32.7 |
| SMAD | PI3K | AXIN2 | Frizzled | 32.8 |
| SMAD | PI3K | AXIN2 | LOXL23 | 32.8 |
| SMAD | PI3K | RKIP | EGR1 | 32.8 |
| SMAD | PI3K | RKIP | STAT | 32.8 |
| SMAD | PI3K | RKIP | STAT | 32.8 |
| SMAD | AKT | Frizzled | β-catenin_nuc | 32.8 |
| SMAD | AKT | LOXL23 | Dest_compl | 32.8 |
| SMAD | AKT | HIF1α | LOXL23 | 32.8 |
| SMAD | PI3K | RKIP | AKT | 32.8 |
| SMAD | PI3K | RKIP | AKT | 32.8 |
| SMAD | AKT | STAT | TCF/LEF | 32.8 |
| SMAD | AKT | STAT | CHD1L | 32.8 |
| SMAD | AKT | SRC | NfκB | 32.8 |
| SMAD | PI3K | RKIP | Csn | 32.8 |
| SMAD | AKT | RKIP | Csn | 32.8 |
| SMAD | AKT | RKIP | Wnt | 32.8 |
| SMAD | AKT | RKIP | Wnt | 32.8 |
| SMAD | AKT | RKIP | ILK | 32.8 |
| SMAD | PI3K | RKIP | SHH | 32.8 |
| SMAD | AKT | RKIP | SHH | 32.8 |
| SMAD | AKT | RKIP | DSH | 32.8 |
| SMAD | PI3K | RKIP | IKKα | 32.9 |
| SMAD | PI3K | RKIP | EGR1 | 32.9 |
| SMAD | PI3K | RKIP | CD44 | 32.9 |
| SMAD | PI3K | RKIP | SRC | 32.9 |
| SMAD | AKT | AXIN2 | Dest_compl | 32.9 |
| SMAD | PI3K | RKIP | AKT | 32.9 |
| SMAD | PI3K | RKIP | AKT | 32.9 |
| SMAD | AKT | c-fos | AXIN2 | 32.9 |
| SMAD | AKT | STAT | LOXL23 | 32.9 |
| SMAD | AKT | GLI | CD44 | 32.9 |
| SMAD | PI3K | RKIP | Csn | 32.9 |
| SMAD | AKT | RKIP | SMO | 32.9 |
| SMAD | PI3K | RKIP | Wnt | 32.9 |
| SMAD | PI3K | RKIP | Wnt | 32.9 |
| SMAD | PI3K | RKIP | ILK | 32.9 |
| SMAD | PI3K | RKIP | ILK | 32.9 |
| SMAD | AKT | RKIP | SHH | 32.9 |
| SMAD | AKT | RKIP | SHH | 32.9 |
| SMAD | AKT | RKIP | SHH | 32.9 |
| SMAD | AKT | RKIP | DSH | 32.9 |
| SMAD | PI3K | Dest_compl | β-catenin_nuc | 33 |
| SMAD | PI3K | TCF/LEF | Dest_compl | 33 |
| SMAD | AKT | cMet | NfκB | 33 |
| SMAD | AKT | c-fos | LIV1 | 33 |
| SMAD | AKT | GLI | Jagged | 33 |
| SMAD | PI3K | RKIP | Csn | 33 |
| SMAD | PI3K | RKIP | SMO | 33 |
| SMAD | PI3K | RKIP | SMO | 33 |
| SMAD | AKT | RKIP | SMO | 33 |
| SMAD | PI3K | RKIP | Wnt | 33 |
| SMAD | PI3K | RKIP | SHH | 33 |
| SMAD | PI3K | RKIP | DSH | 33 |
| SMAD | PI3K | CHD1L | Dest_compl | 33.1 |
| SMAD | PI3K | CHD1L | LOXL23 | 33.1 |
| SMAD | PI3K | RKIP | NfκB | 33.1 |
| SMAD | PI3K | RKIP | EGR1 | 33.1 |
| SMAD | PI3K | RKIP | LIV1 | 33.1 |
| SMAD | PI3K | RKIP | CD44 | 33.1 |
| SMAD | PI3K | RKIP | SRC | 33.1 |
| SMAD | AKT | Jagged | LOXL23 | 33.1 |
| SMAD | PI3K | RKIP | Csn | 33.1 |
| SMAD | PI3K | RKIP | SMO | 33.1 |
| SMAD | AKT | RKIP | SMO | 33.1 |
| SMAD | AKT | RKIP | ILK | 33.1 |
| SMAD | AKT | RKIP | ILK | 33.1 |
| SMAD | PI3K | RKIP | SHH | 33.1 |
| SMAD | AKT | RKIP | SHH | 33.1 |
| SMAD | AKT | RKIP | SHH | 33.1 |
| SMAD | PI3K | RKIP | DSH | 33.1 |
| SMAD | PI3K | RKIP | DSH | 33.1 |
| SMAD | PI3K | RKIP | cMet | 33.2 |
| SMAD | AKT | AXIN2 | LOXL23 | 33.2 |
| SMAD | AKT | IKKα | LOXL23 | 33.2 |
| SMAD | AKT | NfκB | HIF1α | 33.2 |
| SMAD | AKT | LIV1 | TCF/LEF | 33.2 |
| SMAD | AKT | SRC | LOXL23 | 33.2 |
| SMAD | PI3K | RKIP | AKT | 33.2 |
| SMAD | PI3K | RKIP | FUS | 33.2 |
| SMAD | PI3K | RKIP | FUS | 33.2 |
| SMAD | AKT | RKIP | FUS | 33.2 |
| SMAD | AKT | RKIP | ILK | 33.2 |
| SMAD | AKT | RKIP | ILK | 33.2 |
| SMAD | AKT | RKIP | SHH | 33.2 |
| SMAD | PI3K | Frizzled | β-catenin_nuc | 33.3 |
| SMAD | PI3K | AXIN2 | Jagged | 33.3 |
| SMAD | PI3K | RKIP | IKKα | 33.3 |
| SMAD | PI3K | RKIP | NfκB | 33.3 |
| SMAD | PI3K | RKIP | cMet | 33.3 |
| SMAD | PI3K | RKIP | STAT | 33.3 |
| SMAD | AKT | IKKα | Frizzled | 33.3 |
| SMAD | AKT | EGR1 | Jagged | 33.3 |
| SMAD | PI3K | RKIP | AKT | 33.3 |
| SMAD | AKT | LIV1 | IKKα | 33.3 |
| SMAD | AKT | CD44 | IKKα | 33.3 |
| SMAD | PI3K | RKIP | AKT | 33.3 |
| SMAD | AKT | c-fos | NfκB | 33.3 |
| SMAD | PI3K | RKIP | Csn | 33.3 |
| SMAD | PI3K | RKIP | FUS | 33.3 |
| SMAD | PI3K | RKIP | Wnt | 33.3 |
| SMAD | AKT | RKIP | Wnt | 33.3 |
| SMAD | PI3K | RKIP | ILK | 33.3 |
| SMAD | AKT | RKIP | ILK | 33.3 |
| SMAD | PI3K | RKIP | SHH | 33.3 |
| SMAD | AKT | RKIP | DSH | 33.3 |
| SMAD | PI3K | RKIP | CD44 | 33.4 |
| SMAD | PI3K | RKIP | CD44 | 33.4 |
| SMAD | PI3K | RKIP | cMet | 33.4 |
| SMAD | PI3K | RKIP | c-fos | 33.4 |
| SMAD | PI3K | RKIP | STAT | 33.4 |
| SMAD | PI3K | RKIP | STAT | 33.4 |
| SMAD | PI3K | RKIP | SRC | 33.4 |
| SMAD | PI3K | RKIP | SRC | 33.4 |
| SMAD | AKT | Dest_compl | β-catenin_nuc | 33.4 |
| SMAD | AKT | HIF1α | TCF/LEF | 33.4 |
| SMAD | AKT | HIF1α | AXIN2 | 33.4 |
| SMAD | AKT | NfκB | IKKα | 33.4 |
| SMAD | AKT | GLI | c-fos | 33.4 |
| SMAD | AKT | RKIP | Csn | 33.4 |
| SMAD | AKT | RKIP | FUS | 33.4 |
| SMAD | PI3K | RKIP | Wnt | 33.4 |
| SMAD | PI3K | RKIP | Wnt | 33.4 |
| SMAD | AKT | RKIP | DSH | 33.4 |
| SMAD | PI3K | RKIP | STAT | 33.5 |
| SMAD | AKT | IKKα | Jagged | 33.5 |
| SMAD | AKT | NfκB | Jagged | 33.5 |
| SMAD | AKT | LIV1 | Dest_compl | 33.5 |
| SMAD | AKT | STAT | c-fos | 33.5 |
| SMAD | AKT | GLI | Frizzled | 33.5 |
| SMAD | AKT | GLI | LOXL23 | 33.5 |
| SMAD | PI3K | RKIP | AKT | 33.5 |
| SMAD | AKT | SRC | β-catenin_nuc | 33.5 |
| SMAD | PI3K | RKIP | FUS | 33.5 |
| SMAD | PI3K | RKIP | FUS | 33.5 |
| SMAD | PI3K | RKIP | SMO | 33.5 |
| SMAD | PI3K | RKIP | Wnt | 33.5 |
| SMAD | PI3K | RKIP | ILK | 33.5 |
| SMAD | AKT | RKIP | ILK | 33.5 |
| SMAD | AKT | RKIP | ILK | 33.5 |
| SMAD | AKT | RKIP | DSH | 33.5 |
| SMAD | PI3K | Jagged | Frizzled | 33.6 |
| SMAD | PI3K | HIF1α | Frizzled | 33.6 |
| SMAD | PI3K | HIF1α | TCF/LEF | 33.6 |
| SMAD | PI3K | RKIP | cMet | 33.6 |
| SMAD | PI3K | RKIP | STAT | 33.6 |
| SMAD | PI3K | RKIP | AKT | 33.6 |
| SMAD | PI3K | RKIP | AKT | 33.6 |
| SMAD | AKT | c-fos | IKKα | 33.6 |
| SMAD | AKT | GLI | β-catenin_nuc | 33.6 |
| SMAD | PI3K | RKIP | Csn | 33.6 |
| SMAD | PI3K | RKIP | FUS | 33.6 |
| SMAD | AKT | RKIP | SMO | 33.6 |
| SMAD | PI3K | RKIP | ILK | 33.6 |
| SMAD | PI3K | RKIP | SHH | 33.6 |
| SMAD | PI3K | RKIP | SHH | 33.6 |
| SMAD | PI3K | RKIP | IKKα | 33.7 |
| SMAD | PI3K | RKIP | NfκB | 33.7 |
| SMAD | PI3K | RKIP | CD44 | 33.7 |
| SMAD | PI3K | RKIP | cMet | 33.7 |
| SMAD | PI3K | RKIP | SRC | 33.7 |
| SMAD | PI3K | RKIP | SRC | 33.7 |
| SMAD | AKT | IKKα | HIF1α | 33.7 |
| SMAD | AKT | EGR1 | Frizzled | 33.7 |
| SMAD | AKT | STAT | Dest_compl | 33.7 |
| SMAD | AKT | SRC | GLI | 33.7 |
| SMAD | AKT | RKIP | Csn | 33.7 |
| SMAD | AKT | RKIP | Csn | 33.7 |
| SMAD | RKIP | FUS | PAK1 | 33.7 |
| SMAD | AKT | RKIP | FUS | 33.7 |
| SMAD | AKT | RKIP | FUS | 33.7 |
| SMAD | AKT | RKIP | Wnt | 33.7 |
| SMAD | PI3K | RKIP | ILK | 33.7 |
| SMAD | AKT | RKIP | DSH | 33.7 |
| SMAD | PI3K | RKIP | CD44 | 33.8 |
| SMAD | PI3K | RKIP | GLI | 33.8 |
| SMAD | AKT | AXIN2 | Frizzled | 33.8 |
| SMAD | AKT | cMet | CHD1L | 33.8 |
| SMAD | AKT | cMet | LIV1 | 33.8 |
| SMAD | AKT | c-fos | CD44 | 33.8 |
| SMAD | AKT | SRC | EGR1 | 33.8 |
| SMAD | PI3K | RKIP | Csn | 33.8 |
| SMAD | RKIP | Csn | βTrCP | 33.8 |
| SMAD | AKT | RKIP | Csn | 33.8 |
| SMAD | PI3K | RKIP | Wnt | 33.8 |
| SMAD | AKT | RKIP | Wnt | 33.8 |
| SMAD | AKT | RKIP | Wnt | 33.8 |
| SMAD | PI3K | RKIP | NfκB | 33.9 |
| SMAD | RKIP | c-fos | CD44 | 33.9 |
| SMAD | PI3K | RKIP | STAT | 33.9 |
| SMAD | PI3K | RKIP | SRC | 33.9 |
| SMAD | PI3K | RKIP | SRC | 33.9 |
| SMAD | AKT | IKKα | AXIN2 | 33.9 |
| SMAD | AKT | EGR1 | CHD1L | 33.9 |
| SMAD | AKT | STAT | Jagged | 33.9 |
| SMAD | AKT | STAT | NfκB | 33.9 |
| SMAD | PI3K | RKIP | Csn | 33.9 |
| SMAD | PI3K | RKIP | SMO | 33.9 |
| SMAD | PI3K | RKIP | ILK | 33.9 |
| SMAD | PI3K | RKIP | ILK | 33.9 |
| SMAD | AKT | RKIP | SHH | 33.9 |
| SMAD | AKT | RKIP | SHH | 33.9 |
| SMAD | RKIP | miR200 | LOXL23 | 34 |
| SMAD | RKIP | EGR1 | NfκB | 34 |
| SMAD | PI3K | RKIP | cMet | 34 |
| SMAD | PI3K | RKIP | GLI | 34 |
| SMAD | PI3K | RKIP | SRC | 34 |
| SMAD | AKT | AXIN2 | β-catenin_nuc | 34 |
| SMAD | AKT | CD44 | HIF1α | 34 |
| SMAD | AKT | CD44 | LIV1 | 34 |
| SMAD | AKT | SRC | CHD1L | 34 |
| SMAD | AKT | RKIP | FUS | 34 |
| SMAD | AKT | RKIP | SMO | 34 |
| SMAD | AKT | RKIP | SMO | 34 |
| SMAD | AKT | RKIP | ILK | 34 |
| SMAD | AKT | RKIP | ILK | 34 |
| SMAD | AKT | RKIP | SHH | 34 |
| SMAD | AKT | RKIP | SHH | 34 |
| SMAD | PI3K | RKIP | DSH | 34 |
| SMAD | PI3K | Jagged | Dest_compl | 34.1 |
| SMAD | RKIP | CDC42 | HIF1α | 34.1 |
| SMAD | RKIP | IKKα | AXIN2 | 34.1 |
| SMAD | PI3K | RKIP | EGR1 | 34.1 |
| SMAD | RKIP | c-fos | CDC42 | 34.1 |
| SMAD | AKT | CHD1L | Frizzled | 34.1 |
| SMAD | AKT | NfκB | β-catenin_nuc | 34.1 |
| SMAD | AKT | STAT | HIF1α | 34.1 |
| SMAD | AKT | SRC | IKKα | 34.1 |
| SMAD | RKIP | Csn | IKKα | 34.1 |
| SMAD | RKIP | FUS | CHD1L | 34.1 |
| SMAD | AKT | RKIP | FUS | 34.1 |
| SMAD | AKT | RKIP | FUS | 34.1 |
| SMAD | PI3K | RKIP | SMO | 34.1 |
| SMAD | PI3K | RKIP | SMO | 34.1 |
| SMAD | PI3K | RKIP | Wnt | 34.1 |
| SMAD | PI3K | RKIP | Wnt | 34.1 |
| SMAD | AKT | RKIP | Wnt | 34.1 |
| SMAD | PI3K | Jagged | TCF/LEF | 34.2 |
| SMAD | PI3K | RKIP | c-fos | 34.2 |
| SMAD | AKT | Frizzled | Dest_compl | 34.2 |
| SMAD | AKT | c-fos | TCF/LEF | 34.2 |
| SMAD | AKT | SRC | cMet | 34.2 |
| SMAD | PI3K | RKIP | Csn | 34.2 |
| SMAD | AKT | RKIP | Csn | 34.2 |
| SMAD | PI3K | RKIP | SMO | 34.2 |
| SMAD | AKT | RKIP | SMO | 34.2 |
| SMAD | PI3K | RKIP | Wnt | 34.2 |
| SMAD | AKT | RKIP | Wnt | 34.2 |
| SMAD | PI3K | RKIP | Wnt | 34.2 |
| SMAD | PI3K | RKIP | ILK | 34.2 |
| SMAD | PI3K | RKIP | SHH | 34.2 |
| SMAD | RKIP | DSH | Csn | 34.2 |
| SMAD | AKT | RKIP | DSH | 34.2 |
| SMAD | PI3K | RKIP | DSH | 34.2 |
| SMAD | PI3K | TCF/LEF | β-catenin_nuc | 34.3 |
| SMAD | PI3K | LOXL23 | Frizzled | 34.3 |
| SMAD | PI3K | CHD1L | AXIN2 | 34.3 |
| SMAD | PI3K | RKIP | NfκB | 34.3 |
| SMAD | RKIP | EGR1 | LOXL23 | 34.3 |
| SMAD | RKIP | LIV1 | miR200 | 34.3 |
| SMAD | PI3K | RKIP | STAT | 34.3 |
| SMAD | PI3K | RKIP | STAT | 34.3 |
| SMAD | PI3K | RKIP | GLI | 34.3 |
| SMAD | PI3K | RKIP | SRC | 34.3 |
| SMAD | AKT | Patched | Frizzled | 34.3 |
| SMAD | AKT | cMet | EGR1 | 34.3 |
| SMAD | AKT | c-fos | Frizzled | 34.3 |
| SMAD | AKT | RKIP | SMO | 34.3 |
| SMAD | PI3K | RKIP | Wnt | 34.3 |
| SMAD | AKT | RKIP | Wnt | 34.3 |
| SMAD | AKT | RKIP | Wnt | 34.3 |
| SMAD | PI3K | RKIP | ILK | 34.3 |
| SMAD | RKIP | ILK | SMO | 34.3 |
| SMAD | PI3K | RKIP | SHH | 34.3 |
| SMAD | PI3K | RKIP | SHH | 34.3 |
| SMAD | PI3K | RKIP | SHH | 34.3 |
| SMAD | PI3K | RKIP | DSH | 34.3 |
| SMAD | PI3K | RKIP | EGR1 | 34.4 |
| SMAD | PI3K | RKIP | LIV1 | 34.4 |
| SMAD | PI3K | RKIP | CD44 | 34.4 |
| SMAD | PI3K | RKIP | c-fos | 34.4 |
| SMAD | PI3K | RKIP | SRC | 34.4 |
| SMAD | AKT | HIF1α | Jagged | 34.4 |
| SMAD | AKT | STAT | Frizzled | 34.4 |
| SMAD | AKT | SRC | HIF1α | 34.4 |
| SMAD | PI3K | RKIP | FUS | 34.4 |
| SMAD | RKIP | Wnt | Jagged | 34.4 |
| SMAD | AKT | RKIP | Wnt | 34.4 |
| SMAD | AKT | RKIP | ILK | 34.4 |
| SMAD | AKT | RKIP | ILK | 34.4 |
| SMAD | PI3K | RKIP | SHH | 34.4 |
| SMAD | RKIP | SHH | Csn | 34.4 |
| SMAD | AKT | RKIP | SHH | 34.4 |
| SMAD | PI3K | RKIP | DSH | 34.4 |
| SMAD | AKT | RKIP | DSH | 34.4 |
| SMAD | PI3K | RKIP | IKKα | 34.5 |
| SMAD | PI3K | RKIP | STAT | 34.5 |
| SMAD | AKT | LOXL23 | Frizzled | 34.5 |
| SMAD | AKT | NfκB | Frizzled | 34.5 |
| SMAD | AKT | EGR1 | LOXL23 | 34.5 |
| SMAD | AKT | cMet | Dest_compl | 34.5 |
| SMAD | AKT | RKIP | DSH | 34.5 |
| SMAD | PI3K | RKIP | IKKα | 34.6 |
| SMAD | RKIP | PAK1 | Dest_compl | 34.6 |
| SMAD | PI3K | RKIP | cMet | 34.6 |
| SMAD | PI3K | RKIP | GLI | 34.6 |
| SMAD | AKT | TCF/LEF | β-catenin_nuc | 34.6 |
| SMAD | PI3K | RKIP | AKT | 34.6 |
| SMAD | AKT | GLI | cMet | 34.6 |
| SMAD | AKT | GLI | STAT | 34.6 |
| SMAD | AKT | SRC | LIV1 | 34.6 |
| SMAD | AKT | RKIP | Csn | 34.6 |
| SMAD | AKT | RKIP | Csn | 34.6 |
| SMAD | PI3K | RKIP | FUS | 34.6 |
| SMAD | AKT | RKIP | FUS | 34.6 |
| SMAD | PI3K | RKIP | Wnt | 34.6 |
| SMAD | AKT | RKIP | ILK | 34.6 |
| SMAD | AKT | RKIP | SHH | 34.6 |
| SMAD | PI3K | RKIP | DSH | 34.6 |
| SMAD | AKT | RKIP | DSH | 34.6 |
| SMAD | PI3K | LOXL23 | TCF/LEF | 34.7 |
| SMAD | PI3K | RKIP | c-fos | 34.7 |
| SMAD | AKT | AXIN2 | TCF/LEF | 34.7 |
| SMAD | AKT | IKKα | CHD1L | 34.7 |
| SMAD | AKT | GLI | CHD1L | 34.7 |
| SMAD | PI3K | RKIP | FUS | 34.7 |
| SMAD | PI3K | RKIP | FUS | 34.7 |
| SMAD | PI3K | RKIP | SMO | 34.7 |
| SMAD | AKT | RKIP | ILK | 34.7 |
| SMAD | AKT | RKIP | SHH | 34.7 |
| SMAD | AKT | RKIP | DSH | 34.7 |
| SMAD | AKT | RKIP | DSH | 34.7 |
| SMAD | AKT | RKIP | DSH | 34.7 |
| SMAD | AKT | RKIP | DSH | 34.7 |
| SMAD | RKIP | CDC42 | Dest_compl | 34.8 |
| SMAD | PI3K | RKIP | LIV1 | 34.8 |
| SMAD | PI3K | RKIP | STAT | 34.8 |
| SMAD | PI3K | RKIP | GLI | 34.8 |
| SMAD | PI3K | RKIP | GLI | 34.8 |
| SMAD | PI3K | RKIP | SRC | 34.8 |
| SMAD | PI3K | RKIP | Csn | 34.8 |
| SMAD | PI3K | RKIP | FUS | 34.8 |
| SMAD | AKT | RKIP | FUS | 34.8 |
| SMAD | AKT | RKIP | SMO | 34.8 |
| SMAD | PI3K | RKIP | DSH | 34.8 |
| SMAD | PI3K | HIF1α | CHD1L | 34.9 |
| SMAD | RKIP | CDC42 | CHD1L | 34.9 |
| SMAD | RKIP | LIV1 | CHD1L | 34.9 |
| SMAD | PI3K | RKIP | CD44 | 34.9 |
| SMAD | RKIP | cMet | AXIN2 | 34.9 |
| SMAD | RKIP | cMet | IKKα | 34.9 |
| SMAD | PI3K | RKIP | STAT | 34.9 |
| SMAD | PI3K | RKIP | STAT | 34.9 |
| SMAD | PI3K | RKIP | GLI | 34.9 |
| SMAD | PI3K | RKIP | AKT | 34.9 |
| SMAD | AKT | CD44 | Frizzled | 34.9 |
| SMAD | AKT | cMet | TCF/LEF | 34.9 |
| SMAD | AKT | cMet | AXIN2 | 34.9 |
| SMAD | AKT | c-fos | cMet | 34.9 |
| SMAD | AKT | RKIP | FUS | 34.9 |
| SMAD | PI3K | RKIP | SMO | 34.9 |
| SMAD | PI3K | RKIP | SMO | 34.9 |
| SMAD | PI3K | RKIP | SMO | 34.9 |
| SMAD | AKT | RKIP | Wnt | 34.9 |
| SMAD | AKT | RKIP | Wnt | 34.9 |
| SMAD | PI3K | RKIP | ILK | 34.9 |
| SMAD | AKT | RKIP | SHH | 34.9 |
| SMAD | AKT | RKIP | SHH | 34.9 |
| SMAD | AKT | RKIP | SHH | 34.9 |
| SMAD | PI3K | AXIN2 | β-catenin_nuc | 35 |
| SMAD | RKIP | LIV1 | Frizzled | 35 |
| SMAD | PI3K | RKIP | LIV1 | 35 |
| SMAD | RKIP | c-fos | HIF1α | 35 |
| SMAD | PI3K | RKIP | c-fos | 35 |
| SMAD | RKIP | GLI | SUFU | 35 |
| SMAD | AKT | TCF/LEF | Dest_compl | 35 |
| SMAD | PI3K | RKIP | AKT | 35 |
| SMAD | AKT | EGR1 | TCF/LEF | 35 |
| SMAD | PI3K | RKIP | Csn | 35 |
| SMAD | RKIP | Csn | GLI | 35 |
| SMAD | AKT | RKIP | Csn | 35 |
| SMAD | PI3K | RKIP | SMO | 35 |
| SMAD | AKT | RKIP | Wnt | 35 |
| SMAD | AKT | RKIP | Wnt | 35 |
| SMAD | AKT | RKIP | ILK | 35 |
| SMAD | RKIP | DSH | Dest_compl | 35 |
| SMAD | RKIP | DSH | CHD1L | 35 |
| SMAD | RKIP | DSH | SRC | 35 |
| SMAD | PI3K | RKIP | DSH | 35 |
| SMAD | PI3K | SUFU | Frizzled | 35.1 |
| SMAD | PI3K | RKIP | LIV1 | 35.1 |
| SMAD | AKT | CHD1L | Dest_compl | 35.1 |
| SMAD | AKT | EGR1 | β-catenin_nuc | 35.1 |
| SMAD | AKT | EGR1 | HIF1α | 35.1 |
| SMAD | AKT | LIV1 | HIF1α | 35.1 |
| SMAD | AKT | STAT | IKKα | 35.1 |
| SMAD | AKT | SRC | AXIN2 | 35.1 |
| SMAD | PI3K | RKIP | FUS | 35.1 |
| SMAD | AKT | RKIP | FUS | 35.1 |
| SMAD | AKT | RKIP | SMO | 35.1 |
| SMAD | PI3K | RKIP | Wnt | 35.1 |
| SMAD | PI3K | RKIP | Wnt | 35.1 |
| SMAD | RKIP | Wnt | SUFU | 35.1 |
| SMAD | AKT | RKIP | ILK | 35.1 |
| SMAD | PI3K | CDC42 | Dest_compl | 35.2 |
| SMAD | PI3K | RKIP | IKKα | 35.2 |
| SMAD | PI3K | RKIP | EGR1 | 35.2 |
| SMAD | RKIP | GLI | CHD1L | 35.2 |
| SMAD | PI3K | RKIP | GLI | 35.2 |
| SMAD | PI3K | RKIP | GLI | 35.2 |
| SMAD | PI3K | RKIP | SRC | 35.2 |
| SMAD | RKIP | Csn | NfκB | 35.2 |
| SMAD | AKT | RKIP | Csn | 35.2 |
| SMAD | AKT | RKIP | Csn | 35.2 |
| SMAD | RKIP | SHH | c-fos | 35.2 |
| SMAD | AKT | RKIP | DSH | 35.2 |
| SMAD | PI3K | RKIP | DSH | 35.2 |
| SMAD | RKIP | IKKα | CDC42 | 35.3 |
| SMAD | RKIP | NfκB | LOXL23 | 35.3 |
| SMAD | PI3K | RKIP | cMet | 35.3 |
| SMAD | AKT | CHD1L | LOXL23 | 35.3 |
| SMAD | PI3K | RKIP | AKT | 35.3 |
| SMAD | AKT | CD44 | CHD1L | 35.3 |
| SMAD | AKT | STAT | cMet | 35.3 |
| SMAD | PI3K | RKIP | FUS | 35.3 |
| SMAD | RKIP | FUS | CDC42 | 35.3 |
| SMAD | AKT | RKIP | Wnt | 35.3 |
| SMAD | AKT | RKIP | ILK | 35.3 |
| SMAD | AKT | RKIP | ILK | 35.3 |
| SMAD | PI3K | RKIP | SHH | 35.3 |
| SMAD | PI3K | RKIP | SHH | 35.3 |
| SMAD | RKIP | SHH | SRC | 35.3 |
| SMAD | AKT | RKIP | SHH | 35.3 |
| SMAD | PI3K | CDC42 | LOXL23 | 35.4 |
| SMAD | RKIP | CD44 | Frizzled | 35.4 |
| SMAD | RKIP | CD44 | PAK1 | 35.4 |
| SMAD | AKT | LOXL23 | β-catenin_nuc | 35.4 |
| SMAD | AKT | c-fos | Dest_compl | 35.4 |
| SMAD | PI3K | RKIP | Csn | 35.4 |
| SMAD | AKT | RKIP | FUS | 35.4 |
| SMAD | AKT | RKIP | Wnt | 35.4 |
| SMAD | PI3K | RKIP | ILK | 35.4 |
| SMAD | PI3K | RKIP | SHH | 35.4 |
| SMAD | AKT | RKIP | DSH | 35.4 |
| SMAD | PI3K | HIF1α | β-catenin_nuc | 35.5 |
| SMAD | PI3K | RKIP | LIV1 | 35.5 |
| SMAD | RKIP | GLI | AXIN2 | 35.5 |
| SMAD | AKT | cMet | LOXL23 | 35.5 |
| SMAD | RKIP | Csn | β-catenin_nuc | 35.5 |
| SMAD | AKT | RKIP | SMO | 35.5 |
| SMAD | RKIP | SMO | Csn | 35.5 |
| SMAD | RKIP | DSH | SUFU | 35.5 |
| SMAD | RKIP | DSH | FUS | 35.5 |
| SMAD | PI3K | CHD1L | Jagged | 35.6 |
| SMAD | RKIP | βTrCP | Dest_compl | 35.6 |
| SMAD | RKIP | EGR1 | Frizzled | 35.6 |
| SMAD | RKIP | CD44 | βTrCP | 35.6 |
| SMAD | RKIP | c-fos | TCF/LEF | 35.6 |
| SMAD | RKIP | c-fos | LOXL23 | 35.6 |
| SMAD | RKIP | STAT | Dest_compl | 35.6 |
| SMAD | PI3K | RKIP | SRC | 35.6 |
| SMAD | AKT | cMet | Frizzled | 35.6 |
| SMAD | AKT | GLI | Patched | 35.6 |
| SMAD | RKIP | FUS | AXIN2 | 35.6 |
| SMAD | RKIP | SMO | Frizzled | 35.6 |
| SMAD | PI3K | RKIP | SHH | 35.6 |
| SMAD | PI3K | RKIP | DSH | 35.6 |
| SMAD | PI3K | RKIP | DSH | 35.6 |
| SMAD | PI3K | AXIN2 | TCF/LEF | 35.7 |
| SMAD | RKIP | cMet | βTrCP | 35.7 |
| SMAD | PI3K | RKIP | c-fos | 35.7 |
| SMAD | RKIP | STAT | cMet | 35.7 |
| SMAD | AKT | cMet | CDC42 | 35.7 |
| SMAD | AKT | c-fos | Jagged | 35.7 |
| SMAD | AKT | c-fos | HIF1α | 35.7 |
| SMAD | AKT | STAT | AXIN2 | 35.7 |
| SMAD | PI3K | RKIP | AKT | 35.7 |
| SMAD | AKT | GLI | HIF1α | 35.7 |
| SMAD | PI3K | RKIP | Csn | 35.7 |
| SMAD | PI3K | RKIP | Wnt | 35.7 |
| SMAD | RKIP | ILK | β-catenin_nuc | 35.7 |
| SMAD | RKIP | DSH | CD44 | 35.7 |
| SMAD | PI3K | RKIP | GLI | 35.8 |
| SMAD | RKIP | SRC | LIV1 | 35.8 |
| SMAD | RKIP | SMO | Patched | 35.8 |
| SMAD | RKIP | SMO | TCF/LEF | 35.8 |
| SMAD | RKIP | ILK | CDC42 | 35.8 |
| SMAD | RKIP | SHH | CHD1L | 35.8 |
| SMAD | RKIP | DSH | cMet | 35.8 |
| SMAD | PI3K | Patched | Frizzled | 35.9 |
| SMAD | RKIP | Patched | Frizzled | 35.9 |
| SMAD | RKIP | CDC42 | Frizzled | 35.9 |
| SMAD | PI3K | RKIP | PAK1 | 35.9 |
| SMAD | AKT | NfκB | LOXL23 | 35.9 |
| SMAD | RKIP | Csn | CD44 | 35.9 |
| SMAD | RKIP | SMO | HIF1α | 35.9 |
| SMAD | PI3K | RKIP | SMO | 35.9 |
| SMAD | AKT | RKIP | Wnt | 35.9 |
| SMAD | RKIP | ILK | STAT | 35.9 |
| SMAD | RKIP | DSH | PAK1 | 35.9 |
| SMAD | PI3K | RKIP | EGR1 | 36 |
| SMAD | PI3K | RKIP | cMet | 36 |
| SMAD | RKIP | c-fos | LIV1 | 36 |
| SMAD | PI3K | RKIP | c-fos | 36 |
| SMAD | PI3K | RKIP | STAT | 36 |
| SMAD | RKIP | GLI | cMet | 36 |
| SMAD | AKT | βTrCP | LOXL23 | 36 |
| SMAD | AKT | PAK1 | HIF1α | 36 |
| SMAD | AKT | cMet | PAK1 | 36 |
| SMAD | AKT | SRC | CDC42 | 36 |
| SMAD | RKIP | Csn | PAK1 | 36 |
| SMAD | PI3K | RKIP | SMO | 36 |
| SMAD | RKIP | SMO | EGR1 | 36 |
| SMAD | AKT | RKIP | SMO | 36 |
| SMAD | PI3K | RKIP | DSH | 36 |
| SMAD | PI3K | HIF1α | Dest_compl | 36.1 |
| SMAD | RKIP | Jagged | LOXL23 | 36.1 |
| SMAD | RKIP | NfκB | CDC42 | 36.1 |
| SMAD | PI3K | RKIP | EGR1 | 36.1 |
| SMAD | RKIP | PAK1 | Frizzled | 36.1 |
| SMAD | PI3K | RKIP | c-fos | 36.1 |
| SMAD | PI3K | RKIP | GLI | 36.1 |
| SMAD | RKIP | SRC | Jagged | 36.1 |
| SMAD | AKT | AXIN2 | Jagged | 36.1 |
| SMAD | AKT | LIV1 | Jagged | 36.1 |
| SMAD | AKT | STAT | β-catenin_nuc | 36.1 |
| SMAD | AKT | RKIP | Csn | 36.1 |
| SMAD | PI3K | RKIP | FUS | 36.1 |
| SMAD | RKIP | Wnt | CHD1L | 36.1 |
| SMAD | RKIP | Wnt | LIV1 | 36.1 |
| SMAD | AKT | RKIP | SHH | 36.1 |
| SMAD | RKIP | IKKα | β-catenin_nuc | 36.2 |
| SMAD | RKIP | PAK1 | LOXL23 | 36.2 |
| SMAD | RKIP | cMet | miR200 | 36.2 |
| SMAD | RKIP | c-fos | Frizzled | 36.2 |
| SMAD | RKIP | c-fos | EGR1 | 36.2 |
| SMAD | RKIP | SRC | HIF1α | 36.2 |
| SMAD | PI3K | RKIP | AKT | 36.2 |
| SMAD | RKIP | SMO | AXIN2 | 36.2 |
| SMAD | RKIP | SMO | c-fos | 36.2 |
| SMAD | RKIP | Wnt | HIF1α | 36.2 |
| SMAD | RKIP | Wnt | STAT | 36.2 |
| SMAD | PI3K | RKIP | ILK | 36.2 |
| SMAD | AKT | RKIP | ILK | 36.2 |
| SMAD | RKIP | SHH | RAF | 36.2 |
| SMAD | RKIP | DSH | NfκB | 36.2 |
| SMAD | AKT | RKIP | DSH | 36.2 |
| SMAD | PI3K | CHD1L | miR200 | 36.3 |
| SMAD | RKIP | AXIN2 | Dest_compl | 36.3 |
| SMAD | RKIP | PAK1 | miR200 | 36.3 |
| SMAD | PI3K | RKIP | SRC | 36.3 |
| SMAD | AKT | CD44 | miR200 | 36.3 |
| SMAD | RKIP | RAF | Jagged | 36.3 |
| SMAD | RKIP | Wnt | Dest_compl | 36.3 |
| SMAD | PI3K | RKIP | Wnt | 36.3 |
| SMAD | AKT | RKIP | ILK | 36.3 |
| SMAD | AKT | RKIP | DSH | 36.3 |
| SMAD | PI3K | RKIP | NfκB | 36.4 |
| SMAD | RKIP | βTrCP | AXIN2 | 36.4 |
| SMAD | RKIP | EGR1 | β-catenin_nuc | 36.4 |
| SMAD | RKIP | EGR1 | TCF/LEF | 36.4 |
| SMAD | PI3K | RKIP | CD44 | 36.4 |
| SMAD | RKIP | cMet | CD44 | 36.4 |
| SMAD | RKIP | STAT | NfκB | 36.4 |
| SMAD | RKIP | STAT | EGR1 | 36.4 |
| SMAD | RKIP | STAT | LIV1 | 36.4 |
| SMAD | PI3K | RKIP | GLI | 36.4 |
| SMAD | AKT | Jagged | β-catenin_nuc | 36.4 |
| SMAD | AKT | RKIP | FUS | 36.4 |
| SMAD | AKT | RKIP | FUS | 36.4 |
| SMAD | PI3K | RKIP | SMO | 36.4 |
| SMAD | PI3K | RKIP | SHH | 36.4 |
| SMAD | RKIP | CHD1L | miR200 | 36.5 |
| SMAD | RKIP | EGR1 | Jagged | 36.5 |
| SMAD | RKIP | cMet | NfκB | 36.5 |
| SMAD | RKIP | STAT | β-catenin_nuc | 36.5 |
| SMAD | RKIP | STAT | CHD1L | 36.5 |
| SMAD | RKIP | GLI | HIF1α | 36.5 |
| SMAD | RKIP | SRC | c-fos | 36.5 |
| SMAD | AKT | CD44 | Dest_compl | 36.5 |
| SMAD | AKT | STAT | PAK1 | 36.5 |
| SMAD | PI3K | RKIP | Csn | 36.5 |
| SMAD | RKIP | FUS | Dest_compl | 36.5 |
| SMAD | RKIP | SMO | βTrCP | 36.5 |
| SMAD | RKIP | SMO | cMet | 36.5 |
| SMAD | RKIP | RAF | βTrCP | 36.5 |
| SMAD | PI3K | RKIP | SHH | 36.5 |
| SMAD | RKIP | SHH | Frizzled | 36.5 |
| SMAD | PI3K | RKIP | SHH | 36.5 |
| SMAD | RKIP | DSH | STAT | 36.5 |
| SMAD | PI3K | RKIP | IKKα | 36.6 |
| SMAD | RKIP | SUFU | Frizzled | 36.6 |
| SMAD | PI3K | RKIP | c-fos | 36.6 |
| SMAD | RKIP | GLI | c-fos | 36.6 |
| SMAD | AKT | PAK1 | IKKα | 36.6 |
| SMAD | AKT | cMet | IKKα | 36.6 |
| SMAD | AKT | cMet | CD44 | 36.6 |
| SMAD | RKIP | Csn | cMet | 36.6 |
| SMAD | RKIP | FUS | CD44 | 36.6 |
| SMAD | RKIP | SMO | IKKα | 36.6 |
| SMAD | AKT | RKIP | SMO | 36.6 |
| SMAD | RKIP | Wnt | IKKα | 36.6 |
| SMAD | PI3K | RKIP | ILK | 36.6 |
| SMAD | RKIP | ILK | EGR1 | 36.6 |
| SMAD | RKIP | ILK | cMet | 36.6 |
| SMAD | RKIP | SHH | CD44 | 36.6 |
| SMAD | PI3K | RKIP | SHH | 36.6 |
| SMAD | RKIP | DSH | β-catenin_nuc | 36.6 |
| SMAD | PI3K | CDC42 | CHD1L | 36.7 |
| SMAD | PI3K | RKIP | NfκB | 36.7 |
| SMAD | RKIP | AXIN2 | β-catenin_nuc | 36.7 |
| SMAD | RKIP | HIF1α | Jagged | 36.7 |
| SMAD | RKIP | HIF1α | AXIN2 | 36.7 |
| SMAD | RKIP | βTrCP | TCF/LEF | 36.7 |
| SMAD | RKIP | NfκB | β-catenin_nuc | 36.7 |
| SMAD | RKIP | PAK1 | CDC42 | 36.7 |
| SMAD | RKIP | PAK1 | IKKα | 36.7 |
| SMAD | RKIP | LIV1 | EGR1 | 36.7 |
| SMAD | RKIP | SRC | TCF/LEF | 36.7 |
| SMAD | AKT | PAK1 | CDC42 | 36.7 |
| SMAD | AKT | LIV1 | βTrCP | 36.7 |
| SMAD | RKIP | Csn | SRC | 36.7 |
| SMAD | RKIP | FUS | Jagged | 36.7 |
| SMAD | RKIP | FUS | miR200 | 36.7 |
| SMAD | PI3K | RKIP | SMO | 36.7 |
| SMAD | RKIP | Wnt | βTrCP | 36.7 |
| SMAD | AKT | RKIP | ILK | 36.7 |
| SMAD | RKIP | SHH | NfκB | 36.7 |
| SMAD | PI3K | RKIP | DSH | 36.7 |
| SMAD | AKT | RKIP | DSH | 36.7 |
| SMAD | PI3K | βTrCP | Frizzled | 36.8 |
| SMAD | PI3K | RKIP | NfκB | 36.8 |
| SMAD | RKIP | CHD1L | LOXL23 | 36.8 |
| SMAD | RKIP | IKKα | HIF1α | 36.8 |
| SMAD | RKIP | PAK1 | HIF1α | 36.8 |
| SMAD | RKIP | c-fos | β-catenin_nuc | 36.8 |
| SMAD | RKIP | STAT | AXIN2 | 36.8 |
| SMAD | RKIP | SRC | EGR1 | 36.8 |
| SMAD | AKT | HIF1α | CHD1L | 36.8 |
| SMAD | AKT | CDC42 | Frizzled | 36.8 |
| SMAD | AKT | CDC42 | Jagged | 36.8 |
| SMAD | PI3K | RKIP | AKT | 36.8 |
| SMAD | RKIP | RAF | miR200 | 36.8 |
| SMAD | RKIP | RAF | SRC | 36.8 |
| SMAD | AKT | RKIP | Wnt | 36.8 |
| SMAD | RKIP | DSH | Patched | 36.8 |
| SMAD | RKIP | DSH | LIV1 | 36.8 |
| SMAD | RKIP | LOXL23 | Dest_compl | 36.9 |
| SMAD | RKIP | HIF1α | Dest_compl | 36.9 |
| SMAD | RKIP | EGR1 | HIF1α | 36.9 |
| SMAD | RKIP | cMet | Jagged | 36.9 |
| SMAD | RKIP | c-fos | AXIN2 | 36.9 |
| SMAD | RKIP | c-fos | CHD1L | 36.9 |
| SMAD | RKIP | STAT | miR200 | 36.9 |
| SMAD | RKIP | GLI | Frizzled | 36.9 |
| SMAD | PI3K | RKIP | AKT | 36.9 |
| SMAD | AKT | STAT | miR200 | 36.9 |
| SMAD | AKT | GLI | βTrCP | 36.9 |
| SMAD | RKIP | FUS | β-catenin_nuc | 36.9 |
| SMAD | RKIP | SHH | SMO | 36.9 |
| SMAD | AKT | RKIP | DSH | 36.9 |
| SMAD | RKIP | CDC42 | Jagged | 37 |
| SMAD | RKIP | CDC42 | βTrCP | 37 |
| SMAD | RKIP | NfκB | Dest_compl | 37 |
| SMAD | RKIP | NfκB | βTrCP | 37 |
| SMAD | RKIP | PAK1 | CHD1L | 37 |
| SMAD | RKIP | CD44 | NfκB | 37 |
| SMAD | RKIP | STAT | LOXL23 | 37 |
| SMAD | RKIP | STAT | Jagged | 37 |
| SMAD | AKT | miR200 | Jagged | 37 |
| SMAD | AKT | HIF1α | miR200 | 37 |
| SMAD | RKIP | SMO | LOXL23 | 37 |
| SMAD | RKIP | Wnt | TCF/LEF | 37 |
| SMAD | RKIP | Wnt | Csn | 37 |
| SMAD | RKIP | ILK | LOXL23 | 37 |
| SMAD | RKIP | DSH | Jagged | 37 |
| SMAD | PI3K | miR200 | TCF/LEF | 37.1 |
| SMAD | PI3K | βTrCP | AXIN2 | 37.1 |
| SMAD | RKIP | CHD1L | β-catenin_nuc | 37.1 |
| SMAD | RKIP | βTrCP | Jagged | 37.1 |
| SMAD | RKIP | NfκB | miR200 | 37.1 |
| SMAD | RKIP | EGR1 | Dest_compl | 37.1 |
| SMAD | PI3K | RKIP | PAK1 | 37.1 |
| SMAD | RKIP | CD44 | β-catenin_nuc | 37.1 |
| SMAD | RKIP | CD44 | miR200 | 37.1 |
| SMAD | PI3K | RKIP | c-fos | 37.1 |
| SMAD | RKIP | GLI | STAT | 37.1 |
| SMAD | RKIP | SRC | IKKα | 37.1 |
| SMAD | PI3K | RKIP | Csn | 37.1 |
| SMAD | PI3K | RKIP | FUS | 37.1 |
| SMAD | AKT | RKIP | FUS | 37.1 |
| SMAD | RKIP | SMO | CHD1L | 37.1 |
| SMAD | RKIP | ILK | Dest_compl | 37.1 |
| SMAD | PI3K | RKIP | DSH | 37.1 |
| SMAD | PI3K | RKIP | NfκB | 37.2 |
| SMAD | RKIP | NfκB | CHD1L | 37.2 |
| SMAD | RKIP | PAK1 | AXIN2 | 37.2 |
| SMAD | RKIP | PAK1 | NfκB | 37.2 |
| SMAD | RKIP | LIV1 | HIF1α | 37.2 |
| SMAD | RKIP | LIV1 | IKKα | 37.2 |
| SMAD | RKIP | CD44 | LOXL23 | 37.2 |
| SMAD | RKIP | CD44 | EGR1 | 37.2 |
| SMAD | RKIP | c-fos | NfκB | 37.2 |
| SMAD | PI3K | RKIP | SRC | 37.2 |
| SMAD | AKT | CD44 | PAK1 | 37.2 |
| SMAD | RKIP | FUS | LIV1 | 37.2 |
| SMAD | PI3K | RKIP | c-fos | 37.3 |
| SMAD | PI3K | RKIP | STAT | 37.3 |
| SMAD | AKT | IKKα | βTrCP | 37.3 |
| SMAD | AKT | PAK1 | NfκB | 37.3 |
| SMAD | AKT | CD44 | βTrCP | 37.3 |
| SMAD | AKT | c-fos | βTrCP | 37.3 |
| SMAD | RKIP | Csn | TCF/LEF | 37.3 |
| SMAD | RKIP | TCF/LEF | Dest_compl | 37.4 |
| SMAD | PI3K | RKIP | EGR1 | 37.4 |
| SMAD | RKIP | PAK1 | βTrCP | 37.4 |
| SMAD | RKIP | STAT | CDC42 | 37.4 |
| SMAD | PI3K | RKIP | SRC | 37.4 |
| SMAD | RKIP | SRC | Dest_compl | 37.4 |
| SMAD | AKT | EGR1 | miR200 | 37.4 |
| SMAD | AKT | EGR1 | AXIN2 | 37.4 |
| SMAD | AKT | LIV1 | CDC42 | 37.4 |
| SMAD | AKT | cMet | βTrCP | 37.4 |
| SMAD | AKT | GLI | PAK1 | 37.4 |
| SMAD | AKT | SRC | miR200 | 37.4 |
| SMAD | AKT | RKIP | Csn | 37.4 |
| SMAD | RKIP | FUS | IKKα | 37.4 |
| SMAD | RKIP | FUS | GLI | 37.4 |
| SMAD | RKIP | FUS | SRC | 37.4 |
| SMAD | RKIP | SMO | Jagged | 37.4 |
| SMAD | PI3K | RKIP | Wnt | 37.4 |
| SMAD | RKIP | ILK | TCF/LEF | 37.4 |
| SMAD | RKIP | ILK | PAK1 | 37.4 |
| SMAD | RKIP | ILK | c-fos | 37.4 |
| SMAD | PI3K | RKIP | SHH | 37.4 |
| SMAD | RKIP | SHH | TCF/LEF | 37.4 |
| SMAD | PI3K | RKIP | DSH | 37.4 |
| SMAD | RKIP | DSH | AXIN2 | 37.4 |
| SMAD | RKIP | DSH | EGR1 | 37.4 |
| SMAD | RKIP | cMet | LIV1 | 37.5 |
| SMAD | RKIP | STAT | HIF1α | 37.5 |
| SMAD | RKIP | GLI | Dest_compl | 37.5 |
| SMAD | RKIP | GLI | PAK1 | 37.5 |
| SMAD | PI3K | RKIP | SRC | 37.5 |
| SMAD | RKIP | SRC | GLI | 37.5 |
| SMAD | AKT | PAK1 | TCF/LEF | 37.5 |
| SMAD | AKT | CD44 | CDC42 | 37.5 |
| SMAD | PI3K | RKIP | AKT | 37.5 |
| SMAD | RKIP | FUS | STAT | 37.5 |
| SMAD | PI3K | RKIP | SMO | 37.5 |
| SMAD | RKIP | SMO | Dest_compl | 37.5 |
| SMAD | RKIP | RAF | GLI | 37.5 |
| SMAD | PI3K | RKIP | Wnt | 37.5 |
| SMAD | RKIP | ILK | CHD1L | 37.5 |
| SMAD | RKIP | SHH | Dest_compl | 37.5 |
| SMAD | RKIP | DSH | c-fos | 37.5 |
| SMAD | PI3K | CDC42 | Jagged | 37.6 |
| SMAD | RKIP | HIF1α | miR200 | 37.6 |
| SMAD | RKIP | EGR1 | AXIN2 | 37.6 |
| SMAD | RKIP | EGR1 | CDC42 | 37.6 |
| SMAD | PI3K | RKIP | PAK1 | 37.6 |
| SMAD | PI3K | RKIP | CD44 | 37.6 |
| SMAD | RKIP | cMet | LOXL23 | 37.6 |
| SMAD | RKIP | c-fos | cMet | 37.6 |
| SMAD | RKIP | GLI | EGR1 | 37.6 |
| SMAD | RKIP | RAF | SMO | 37.6 |
| SMAD | AKT | RKIP | ILK | 37.6 |
| SMAD | RKIP | SHH | CDC42 | 37.6 |
| SMAD | RKIP | AXIN2 | miR200 | 37.7 |
| SMAD | PI3K | RKIP | cMet | 37.7 |
| SMAD | RKIP | c-fos | Dest_compl | 37.7 |
| SMAD | RKIP | STAT | IKKα | 37.7 |
| SMAD | RKIP | GLI | LOXL23 | 37.7 |
| SMAD | RKIP | SRC | Frizzled | 37.7 |
| SMAD | RKIP | SRC | AXIN2 | 37.7 |
| SMAD | RKIP | Csn | AXIN2 | 37.7 |
| SMAD | RKIP | Csn | CDC42 | 37.7 |
| SMAD | RKIP | FUS | cMet | 37.7 |
| SMAD | RKIP | Wnt | EGR1 | 37.7 |
| SMAD | RKIP | Wnt | GLI | 37.7 |
| SMAD | RKIP | SHH | LOXL23 | 37.7 |
| SMAD | RKIP | DSH | RAF | 37.7 |
| SMAD | RKIP | DSH | Wnt | 37.7 |
| SMAD | RKIP | AXIN2 | Jagged | 37.8 |
| SMAD | RKIP | CDC42 | β-catenin_nuc | 37.8 |
| SMAD | RKIP | CDC42 | TCF/LEF | 37.8 |
| SMAD | RKIP | EGR1 | miR200 | 37.8 |
| SMAD | PI3K | RKIP | PAK1 | 37.8 |
| SMAD | PI3K | RKIP | PAK1 | 37.8 |
| SMAD | RKIP | LIV1 | Jagged | 37.8 |
| SMAD | RKIP | SRC | CD44 | 37.8 |
| SMAD | AKT | CDC42 | LOXL23 | 37.8 |
| SMAD | AKT | CDC42 | HIF1α | 37.8 |
| SMAD | RKIP | Csn | Frizzled | 37.8 |
| SMAD | RKIP | Wnt | SMO | 37.8 |
| SMAD | RKIP | ILK | GLI | 37.8 |
| SMAD | RKIP | ILK | RAF | 37.8 |
| SMAD | RKIP | SHH | miR200 | 37.8 |
| SMAD | PI3K | RKIP | DSH | 37.8 |
| SMAD | PI3K | βTrCP | CHD1L | 37.9 |
| SMAD | RKIP | AXIN2 | TCF/LEF | 37.9 |
| SMAD | RKIP | CHD1L | Jagged | 37.9 |
| SMAD | PI3K | RKIP | LIV1 | 37.9 |
| SMAD | RKIP | LIV1 | PAK1 | 37.9 |
| SMAD | RKIP | cMet | TCF/LEF | 37.9 |
| SMAD | RKIP | STAT | Frizzled | 37.9 |
| SMAD | RKIP | GLI | Jagged | 37.9 |
| SMAD | RKIP | SRC | CDC42 | 37.9 |
| SMAD | RKIP | FUS | NfκB | 37.9 |
| SMAD | PI3K | RKIP | FUS | 37.9 |
| SMAD | RKIP | FUS | c-fos | 37.9 |
| SMAD | RKIP | SMO | β-catenin_nuc | 37.9 |
| SMAD | RKIP | ILK | HIF1α | 37.9 |
| SMAD | RKIP | ILK | IKKα | 37.9 |
| SMAD | RKIP | SHH | FUS | 37.9 |
| SMAD | RKIP | DSH | IKKα | 37.9 |
| SMAD | RKIP | DSH | ILK | 37.9 |
| SMAD | PI3K | CDC42 | Frizzled | 38 |
| SMAD | RKIP | Frizzled | Dest_compl | 38 |
| SMAD | RKIP | miR200 | Jagged | 38 |
| SMAD | RKIP | AXIN2 | LOXL23 | 38 |
| SMAD | RKIP | CHD1L | Frizzled | 38 |
| SMAD | RKIP | IKKα | Dest_compl | 38 |
| SMAD | RKIP | IKKα | Jagged | 38 |
| SMAD | RKIP | IKKα | βTrCP | 38 |
| SMAD | RKIP | EGR1 | βTrCP | 38 |
| SMAD | RKIP | CD44 | Dest_compl | 38 |
| SMAD | RKIP | CD44 | TCF/LEF | 38 |
| SMAD | RKIP | cMet | β-catenin_nuc | 38 |
| SMAD | AKT | CDC42 | βTrCP | 38 |
| SMAD | RKIP | FUS | Frizzled | 38 |
| SMAD | RKIP | FUS | Csn | 38 |
| SMAD | RKIP | RAF | EGR1 | 38 |
| SMAD | RKIP | RAF | CD44 | 38 |
| SMAD | RKIP | Wnt | LOXL23 | 38 |
| SMAD | RKIP | SHH | IKKα | 38 |
| SMAD | RKIP | DSH | SHH | 38 |
| SMAD | PI3K | CDC42 | AXIN2 | 38.1 |
| SMAD | PI3K | RKIP | IKKα | 38.1 |
| SMAD | RKIP | Jagged | TCF/LEF | 38.1 |
| SMAD | RKIP | HIF1α | LOXL23 | 38.1 |
| SMAD | RKIP | EGR1 | CHD1L | 38.1 |
| SMAD | PI3K | RKIP | PAK1 | 38.1 |
| SMAD | RKIP | PAK1 | TCF/LEF | 38.1 |
| SMAD | RKIP | LIV1 | β-catenin_nuc | 38.1 |
| SMAD | RKIP | cMet | Dest_compl | 38.1 |
| SMAD | RKIP | GLI | TCF/LEF | 38.1 |
| SMAD | RKIP | GLI | CDC42 | 38.1 |
| SMAD | RKIP | SRC | miR200 | 38.1 |
| SMAD | AKT | PAK1 | Jagged | 38.1 |
| SMAD | RKIP | FUS | HIF1α | 38.1 |
| SMAD | AKT | RKIP | FUS | 38.1 |
| SMAD | RKIP | ILK | miR200 | 38.1 |
| SMAD | RKIP | ILK | AXIN2 | 38.1 |
| SMAD | RKIP | SHH | STAT | 38.1 |
| SMAD | RKIP | SHH | GLI | 38.1 |
| SMAD | AKT | RKIP | SHH | 38.1 |
| SMAD | AKT | RKIP | DSH | 38.1 |
| SMAD | RKIP | DSH | SMO | 38.1 |
| SMAD | RKIP | Jagged | Dest_compl | 38.2 |
| SMAD | PI3K | RKIP | EGR1 | 38.2 |
| SMAD | PI3K | RKIP | PAK1 | 38.2 |
| SMAD | RKIP | LIV1 | βTrCP | 38.2 |
| SMAD | RKIP | CD44 | AXIN2 | 38.2 |
| SMAD | RKIP | STAT | PAK1 | 38.2 |
| SMAD | RKIP | GLI | NfκB | 38.2 |
| SMAD | PI3K | RKIP | SRC | 38.2 |
| SMAD | RKIP | SRC | CHD1L | 38.2 |
| SMAD | AKT | βTrCP | AXIN2 | 38.2 |
| SMAD | RKIP | SMO | GLI | 38.2 |
| SMAD | RKIP | RAF | HIF1α | 38.2 |
| SMAD | RKIP | RAF | CDC42 | 38.2 |
| SMAD | RKIP | Wnt | c-fos | 38.2 |
| SMAD | AKT | RKIP | SHH | 38.2 |
| SMAD | RKIP | DSH | βTrCP | 38.2 |
| SMAD | RKIP | miR200 | TCF/LEF | 38.3 |
| SMAD | RKIP | NfκB | HIF1α | 38.3 |
| SMAD | RKIP | PAK1 | β-catenin_nuc | 38.3 |
| SMAD | RKIP | GLI | Patched | 38.3 |
| SMAD | RKIP | SRC | STAT | 38.3 |
| SMAD | AKT | miR200 | TCF/LEF | 38.3 |
| SMAD | AKT | βTrCP | Dest_compl | 38.3 |
| SMAD | AKT | CDC42 | Dest_compl | 38.3 |
| SMAD | AKT | CDC42 | AXIN2 | 38.3 |
| SMAD | AKT | NfκB | TCF/LEF | 38.3 |
| SMAD | RKIP | Csn | Jagged | 38.3 |
| SMAD | AKT | RKIP | Csn | 38.3 |
| SMAD | RKIP | SMO | SRC | 38.3 |
| SMAD | RKIP | Wnt | β-catenin_nuc | 38.3 |
| SMAD | RKIP | Wnt | CDC42 | 38.3 |
| SMAD | RKIP | Wnt | PAK1 | 38.3 |
| SMAD | RKIP | SHH | β-catenin_nuc | 38.3 |
| SMAD | RKIP | SHH | Wnt | 38.3 |
| SMAD | RKIP | DSH | LOXL23 | 38.3 |
| SMAD | RKIP | AXIN2 | Frizzled | 38.4 |
| SMAD | RKIP | βTrCP | LOXL23 | 38.4 |
| SMAD | RKIP | βTrCP | CHD1L | 38.4 |
| SMAD | RKIP | IKKα | miR200 | 38.4 |
| SMAD | RKIP | NfκB | TCF/LEF | 38.4 |
| SMAD | RKIP | EGR1 | IKKα | 38.4 |
| SMAD | RKIP | CD44 | IKKα | 38.4 |
| SMAD | RKIP | c-fos | miR200 | 38.4 |
| SMAD | RKIP | GLI | LIV1 | 38.4 |
| SMAD | RKIP | Csn | HIF1α | 38.4 |
| SMAD | RKIP | Csn | c-fos | 38.4 |
| SMAD | RKIP | RAF | PAK1 | 38.4 |
| SMAD | RKIP | Wnt | Patched | 38.4 |
| SMAD | RKIP | ILK | FUS | 38.4 |
| SMAD | PI3K | RKIP | DSH | 38.4 |
| SMAD | PI3K | RKIP | NfκB | 38.5 |
| SMAD | RKIP | HIF1α | β-catenin_nuc | 38.5 |
| SMAD | RKIP | PAK1 | EGR1 | 38.5 |
| SMAD | RKIP | cMet | CHD1L | 38.5 |
| SMAD | RKIP | cMet | HIF1α | 38.5 |
| SMAD | RKIP | c-fos | βTrCP | 38.5 |
| SMAD | PI3K | RKIP | SRC | 38.5 |
| SMAD | AKT | SUFU | Frizzled | 38.5 |
| SMAD | AKT | NfκB | CDC42 | 38.5 |
| SMAD | AKT | PAK1 | CHD1L | 38.5 |
| SMAD | RKIP | SMO | miR200 | 38.5 |
| SMAD | RKIP | RAF | STAT | 38.5 |
| SMAD | RKIP | Wnt | miR200 | 38.5 |
| SMAD | RKIP | Wnt | AXIN2 | 38.5 |
| SMAD | RKIP | Wnt | FUS | 38.5 |
| SMAD | RKIP | SHH | ILK | 38.5 |
| SMAD | PI3K | βTrCP | LOXL23 | 38.6 |
| SMAD | RKIP | miR200 | Dest_compl | 38.6 |
| SMAD | RKIP | miR200 | Frizzled | 38.6 |
| SMAD | RKIP | βTrCP | miR200 | 38.6 |
| SMAD | RKIP | CDC42 | AXIN2 | 38.6 |
| SMAD | RKIP | CD44 | CDC42 | 38.6 |
| SMAD | RKIP | c-fos | IKKα | 38.6 |
| SMAD | RKIP | STAT | βTrCP | 38.6 |
| SMAD | RKIP | STAT | CD44 | 38.6 |
| SMAD | RKIP | GLI | β-catenin_nuc | 38.6 |
| SMAD | RKIP | GLI | βTrCP | 38.6 |
| SMAD | AKT | NfκB | miR200 | 38.6 |
| SMAD | AKT | PAK1 | AXIN2 | 38.6 |
| SMAD | AKT | SRC | PAK1 | 38.6 |
| SMAD | AKT | RKIP | SMO | 38.6 |
| SMAD | RKIP | RAF | Dest_compl | 38.6 |
| SMAD | PI3K | RKIP | Wnt | 38.6 |
| SMAD | RKIP | Wnt | SRC | 38.6 |
| SMAD | AKT | RKIP | Wnt | 38.6 |
| SMAD | PI3K | RKIP | ILK | 38.6 |
| SMAD | RKIP | SHH | EGR1 | 38.6 |
| SMAD | PI3K | miR200 | β-catenin_nuc | 38.7 |
| SMAD | PI3K | βTrCP | Dest_compl | 38.7 |
| SMAD | RKIP | LOXL23 | Frizzled | 38.7 |
| SMAD | RKIP | miR200 | β-catenin_nuc | 38.7 |
| SMAD | RKIP | IKKα | TCF/LEF | 38.7 |
| SMAD | RKIP | IKKα | LOXL23 | 38.7 |
| SMAD | RKIP | IKKα | CHD1L | 38.7 |
| SMAD | PI3K | RKIP | LIV1 | 38.7 |
| SMAD | RKIP | LIV1 | LOXL23 | 38.7 |
| SMAD | RKIP | c-fos | PAK1 | 38.7 |
| SMAD | RKIP | SRC | NfκB | 38.7 |
| SMAD | AKT | βTrCP | Jagged | 38.7 |
| SMAD | RKIP | Csn | EGR1 | 38.7 |
| SMAD | RKIP | ILK | LIV1 | 38.7 |
| SMAD | RKIP | ILK | CD44 | 38.7 |
| SMAD | RKIP | ILK | SRC | 38.7 |
| SMAD | RKIP | SHH | AXIN2 | 38.7 |
| SMAD | RKIP | SHH | LIV1 | 38.7 |
| SMAD | RKIP | Dest_compl | β-catenin_nuc | 38.8 |
| SMAD | RKIP | CHD1L | TCF/LEF | 38.8 |
| SMAD | RKIP | NfκB | AXIN2 | 38.8 |
| SMAD | RKIP | LIV1 | AXIN2 | 38.8 |
| SMAD | RKIP | cMet | EGR1 | 38.8 |
| SMAD | AKT | miR200 | β-catenin_nuc | 38.8 |
| SMAD | AKT | IKKα | CDC42 | 38.8 |
| SMAD | RKIP | FUS | Patched | 38.8 |
| SMAD | RKIP | RAF | CHD1L | 38.8 |
| SMAD | RKIP | RAF | LIV1 | 38.8 |
| SMAD | PI3K | miR200 | Jagged | 38.9 |
| SMAD | PI3K | RKIP | IKKα | 38.9 |
| SMAD | RKIP | CDC42 | LOXL23 | 38.9 |
| SMAD | PI3K | RKIP | CD44 | 38.9 |
| SMAD | RKIP | CD44 | HIF1α | 38.9 |
| SMAD | RKIP | GLI | IKKα | 38.9 |
| SMAD | RKIP | SRC | LOXL23 | 38.9 |
| SMAD | AKT | EGR1 | βTrCP | 38.9 |
| SMAD | PI3K | RKIP | SMO | 38.9 |
| SMAD | RKIP | RAF | c-fos | 38.9 |
| SMAD | RKIP | Wnt | Frizzled | 38.9 |
| SMAD | RKIP | Wnt | cMet | 38.9 |
| SMAD | RKIP | SHH | βTrCP | 38.9 |
| SMAD | RKIP | SHH | PAK1 | 38.9 |
| SMAD | RKIP | SHH | cMet | 38.9 |
| SMAD | PI3K | CDC42 | HIF1α | 39 |
| SMAD | RKIP | CHD1L | Dest_compl | 39 |
| SMAD | RKIP | CHD1L | AXIN2 | 39 |
| SMAD | RKIP | NfκB | Jagged | 39 |
| SMAD | PI3K | RKIP | cMet | 39 |
| SMAD | RKIP | STAT | TCF/LEF | 39 |
| SMAD | AKT | PAK1 | Frizzled | 39 |
| SMAD | AKT | PAK1 | βTrCP | 39 |
| SMAD | PI3K | RKIP | SHH | 39 |
| SMAD | RKIP | DSH | Frizzled | 39 |
| SMAD | PI3K | miR200 | LOXL23 | 39.1 |
| SMAD | PI3K | RKIP | PAK1 | 39.1 |
| SMAD | RKIP | cMet | CDC42 | 39.1 |
| SMAD | RKIP | STAT | c-fos | 39.1 |
| SMAD | PI3K | RKIP | GLI | 39.1 |
| SMAD | RKIP | GLI | CD44 | 39.1 |
| SMAD | RKIP | SMO | CD44 | 39.1 |
| SMAD | RKIP | RAF | Csn | 39.1 |
| SMAD | AKT | RKIP | SHH | 39.1 |
| SMAD | PI3K | CDC42 | βTrCP | 39.2 |
| SMAD | RKIP | HIF1α | CHD1L | 39.2 |
| SMAD | PI3K | RKIP | PAK1 | 39.2 |
| SMAD | PI3K | RKIP | CD44 | 39.2 |
| SMAD | RKIP | SRC | β-catenin_nuc | 39.2 |
| SMAD | AKT | IKKα | miR200 | 39.2 |
| SMAD | PI3K | RKIP | FUS | 39.2 |
| SMAD | RKIP | RAF | AXIN2 | 39.2 |
| SMAD | RKIP | ILK | Frizzled | 39.2 |
| SMAD | RKIP | DSH | CDC42 | 39.2 |
| SMAD | RKIP | LOXL23 | TCF/LEF | 39.3 |
| SMAD | RKIP | βTrCP | HIF1α | 39.3 |
| SMAD | RKIP | LIV1 | NfκB | 39.3 |
| SMAD | PI3K | RKIP | CD44 | 39.3 |
| SMAD | RKIP | SRC | βTrCP | 39.3 |
| SMAD | RKIP | Csn | LOXL23 | 39.3 |
| SMAD | RKIP | Csn | LIV1 | 39.3 |
| SMAD | RKIP | SMO | CDC42 | 39.3 |
| SMAD | PI3K | RKIP | ILK | 39.3 |
| SMAD | RKIP | DSH | TCF/LEF | 39.3 |
| SMAD | PI3K | RKIP | NfκB | 39.4 |
| SMAD | RKIP | NfκB | IKKα | 39.4 |
| SMAD | PI3K | RKIP | PAK1 | 39.4 |
| SMAD | RKIP | cMet | Frizzled | 39.4 |
| SMAD | AKT | PAK1 | β-catenin_nuc | 39.4 |
| SMAD | RKIP | Csn | Dest_compl | 39.4 |
| SMAD | RKIP | Csn | CHD1L | 39.4 |
| SMAD | AKT | RKIP | SMO | 39.4 |
| SMAD | RKIP | DSH | miR200 | 39.4 |
| SMAD | PI3K | βTrCP | β-catenin_nuc | 39.5 |
| SMAD | PI3K | βTrCP | HIF1α | 39.5 |
| SMAD | PI3K | RKIP | cMet | 39.5 |
| SMAD | AKT | miR200 | Frizzled | 39.5 |
| SMAD | AKT | cMet | miR200 | 39.5 |
| SMAD | AKT | STAT | CDC42 | 39.5 |
| SMAD | AKT | RKIP | FUS | 39.5 |
| SMAD | AKT | RKIP | Wnt | 39.5 |
| SMAD | AKT | RKIP | Wnt | 39.5 |
| SMAD | RKIP | Wnt | RAF | 39.5 |
| SMAD | RKIP | DSH | GLI | 39.5 |
| SMAD | RKIP | HIF1α | Frizzled | 39.6 |
| SMAD | RKIP | βTrCP | Frizzled | 39.6 |
| SMAD | RKIP | LIV1 | TCF/LEF | 39.6 |
| SMAD | PI3K | RKIP | cMet | 39.6 |
| SMAD | RKIP | SRC | PAK1 | 39.6 |
| SMAD | AKT | βTrCP | HIF1α | 39.6 |
| SMAD | AKT | CDC42 | CHD1L | 39.6 |
| SMAD | PI3K | RKIP | FUS | 39.6 |
| SMAD | RKIP | FUS | EGR1 | 39.6 |
| SMAD | RKIP | SMO | LIV1 | 39.6 |
| SMAD | RKIP | SMO | STAT | 39.6 |
| SMAD | RKIP | ILK | Wnt | 39.6 |
| SMAD | RKIP | DSH | HIF1α | 39.6 |
| SMAD | RKIP | cMet | PAK1 | 39.7 |
| SMAD | AKT | miR200 | Dest_compl | 39.7 |
| SMAD | AKT | CDC42 | β-catenin_nuc | 39.7 |
| SMAD | AKT | CDC42 | TCF/LEF | 39.7 |
| SMAD | AKT | RKIP | Csn | 39.7 |
| SMAD | RKIP | RAF | IKKα | 39.7 |
| SMAD | RKIP | Wnt | NfκB | 39.7 |
| SMAD | RKIP | NfκB | Frizzled | 39.8 |
| SMAD | PI3K | RKIP | PAK1 | 39.8 |
| SMAD | AKT | PAK1 | LOXL23 | 39.8 |
| SMAD | AKT | LIV1 | PAK1 | 39.8 |
| SMAD | RKIP | Csn | STAT | 39.8 |
| SMAD | RKIP | SMO | NfκB | 39.8 |
| SMAD | RKIP | SMO | FUS | 39.8 |
| SMAD | RKIP | ILK | Jagged | 39.8 |
| SMAD | AKT | RKIP | ILK | 39.8 |
| SMAD | RKIP | CD44 | Jagged | 39.9 |
| SMAD | PI3K | RKIP | c-fos | 39.9 |
| SMAD | RKIP | Csn | miR200 | 39.9 |
| SMAD | RKIP | FUS | TCF/LEF | 39.9 |
| SMAD | RKIP | RAF | LOXL23 | 39.9 |
| SMAD | PI3K | βTrCP | TCF/LEF | 40 |
| SMAD | RKIP | Jagged | Frizzled | 40 |
| SMAD | RKIP | IKKα | Frizzled | 40 |
| SMAD | PI3K | RKIP | STAT | 40 |
| SMAD | RKIP | SRC | cMet | 40 |
| SMAD | AKT | STAT | βTrCP | 40 |
| SMAD | PI3K | RKIP | Wnt | 40 |
| SMAD | RKIP | SHH | Jagged | 40 |
| SMAD | RKIP | TCF/LEF | β-catenin_nuc | 40.1 |
| SMAD | RKIP | CDC42 | miR200 | 40.1 |
| SMAD | RKIP | PAK1 | Jagged | 40.1 |
| SMAD | RKIP | LIV1 | CDC42 | 40.1 |
| SMAD | AKT | βTrCP | TCF/LEF | 40.1 |
| SMAD | RKIP | RAF | Frizzled | 40.1 |
| SMAD | PI3K | CDC42 | TCF/LEF | 40.2 |
| SMAD | RKIP | Jagged | β-catenin_nuc | 40.2 |
| SMAD | PI3K | RKIP | LIV1 | 40.2 |
| SMAD | AKT | miR200 | LOXL23 | 40.2 |
| SMAD | PI3K | RKIP | DSH | 40.2 |
| SMAD | RKIP | TCF/LEF | Frizzled | 40.3 |
| SMAD | RKIP | βTrCP | β-catenin_nuc | 40.3 |
| SMAD | PI3K | RKIP | PAK1 | 40.3 |
| SMAD | AKT | AXIN2 | miR200 | 40.3 |
| SMAD | AKT | RKIP | SHH | 40.3 |
| SMAD | AKT | βTrCP | β-catenin_nuc | 40.4 |
| SMAD | AKT | LIV1 | miR200 | 40.4 |
| SMAD | PI3K | RKIP | SMO | 40.4 |
| SMAD | PI3K | RKIP | SMO | 40.4 |
| SMAD | PI3K | HIF1α | miR200 | 40.5 |
| SMAD | RKIP | LIV1 | Dest_compl | 40.5 |
| SMAD | PI3K | RKIP | STAT | 40.5 |
| SMAD | AKT | βTrCP | CHD1L | 40.5 |
| SMAD | AKT | c-fos | miR200 | 40.5 |
| SMAD | RKIP | RAF | NfκB | 40.5 |
| SMAD | RKIP | ILK | βTrCP | 40.5 |
| SMAD | RKIP | LOXL23 | β-catenin_nuc | 40.6 |
| SMAD | PI3K | RKIP | GLI | 40.6 |
| SMAD | RKIP | GLI | miR200 | 40.6 |
| SMAD | AKT | PAK1 | Dest_compl | 40.6 |
| SMAD | RKIP | FUS | βTrCP | 40.6 |
| SMAD | RKIP | RAF | β-catenin_nuc | 40.6 |
| SMAD | RKIP | Frizzled | β-catenin_nuc | 40.7 |
| SMAD | PI3K | RKIP | PAK1 | 40.7 |
| SMAD | PI3K | RKIP | GLI | 40.7 |
| SMAD | AKT | RKIP | Wnt | 40.7 |
| SMAD | AKT | RKIP | ILK | 40.7 |
| SMAD | AKT | GLI | CDC42 | 40.8 |
| SMAD | AKT | RKIP | Csn | 40.8 |
| SMAD | AKT | RKIP | DSH | 40.8 |
| SMAD | PI3K | CDC42 | β-catenin_nuc | 40.9 |
| SMAD | AKT | NfκB | βTrCP | 41 |
| SMAD | PI3K | RKIP | ILK | 41 |
| SMAD | RKIP | SHH | HIF1α | 41 |
| SMAD | AKT | SRC | βTrCP | 41.1 |
| SMAD | PI3K | RKIP | ILK | 41.1 |
| SMAD | PI3K | miR200 | Dest_compl | 41.2 |
| SMAD | PI3K | miR200 | Frizzled | 41.2 |
| SMAD | RKIP | SMO | PAK1 | 41.2 |
| SMAD | AKT | RKIP | SMO | 41.2 |
| SMAD | RKIP | HIF1α | TCF/LEF | 41.3 |
| SMAD | AKT | βTrCP | Frizzled | 41.3 |
| SMAD | RKIP | ILK | Csn | 41.3 |
| SMAD | RKIP | c-fos | Jagged | 41.4 |
| SMAD | PI3K | AXIN2 | miR200 | 41.5 |
| SMAD | RKIP | CD44 | LIV1 | 41.5 |
| SMAD | PI3K | RKIP | GLI | 41.5 |
| SMAD | AKT | βTrCP | miR200 | 41.5 |
| SMAD | PI3K | RKIP | Csn | 41.5 |
| SMAD | RKIP | RAF | TCF/LEF | 41.5 |
| SMAD | PI3K | βTrCP | Jagged | 41.6 |
| SMAD | AKT | GLI | miR200 | 41.6 |
| SMAD | AKT | RKIP | FUS | 41.6 |
| SMAD | AKT | CHD1L | miR200 | 41.7 |
| SMAD | AKT | RKIP | ILK | 41.7 |
| SMAD | RKIP | ILK | NfκB | 42 |
| SMAD | RKIP | RAF | FUS | 42.1 |
| SMAD | RKIP | RAF | cMet | 42.2 |
| SMAD | PI3K | βTrCP | miR200 | 42.9 |
| SMAD | RKIP | CD44 | CHD1L | 43.6 |
| SMAD | PI3K | CDC42 | miR200 | 45.2 |
| SMAD | AKT | CDC42 | miR200 | 45.3 |
| SMAD | AKT | PAK1 | miR200 | 45.5 |
| SMAD | PI3K | RKIP | PAK1 | 48.4 |
| SMAD | AKT | c-fos | PAK1 | 76.3 |
| SMAD | AKT | c-fos | CDC42 | 76.5 |
| SMAD | PI3K | RKIP | PAK1 | 76.9 |
| SMAD | PI3K | RKIP | c-fos | 77 |
| SMAD | PI3K | RKIP | EGR1 | 77.2 |
| SMAD | PI3K | RKIP | c-fos | 78 |
| SMAD | AKT | EGR1 | CDC42 | 79.1 |
| SMAD | AKT | PAK1 | EGR1 | 79.8 |

**Supplemental Table 8: Single node constitutive activations that suppress EMT.** We systematically explore how constitutively activating each separate node (except for TGFβ, TGFβR, E-cadherin, and EMT) affects EMT. Starting from the epithelial steady state, we fix TGFβ=ON and the node of interest to ON, update the system for over 10,000 steps, and check the state of the EMT node after the last update. Every node’s constitutive activation is repeated for
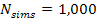
 simulations. The EMT % is accurate to the first or second digit of the percentage, as measured by the standard deviation of the mean of the estimated EMT percentage,
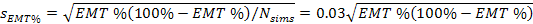
 (e.g., if EMT %
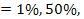
or
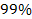
 then the standard deviation of the mean are
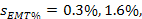
 or
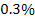
, respectively). The EMT % is given by the average number of simulations at the end of which the EMT node was found in the ON state. The nodes not shown in the table do not have any effect on the EMT %, i.e., they result in an EMT % = 100 %.

| **Node** | **% EMT**  **(n=1000 simulations)** |
| --- | --- |
| miR200 | 0.0 |

**Supplemental Table 9: Double node perturbation combinations of mixed activation-inhibition perturbation that suppress EMT.** We systematically explore how knocking out or constitutively activating combinations of two nodes (except for TGFβ, TGFβR, E-cadherin, and EMT) affects EMT. Starting from the epithelial steady state, we fix TGFβ=ON and the nodes of interest to ON or OFF, update the system for over 10,000 steps, and check the state of the EMT node after the last update. Every knockout combination is repeated for
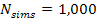
 simulations. The single nodes whose knockout or constitutive activation fully suppressed EMT in Supplemental Table 4 or Supplemental Table 8 are not included in this analysis. Node perturbations where both nodes are inactivated in the combination are included in Supplemental Figure 5. The EMT % is accurate to the first or second digit of the percentage, as measured by the standard deviation of the mean of the estimated EMT percentage,
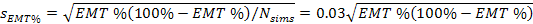
 (e.g., if EMT %
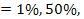
or then the standard deviation of the mean are or , respectively). All double node perturbation combinations that suppress the EMT % include SMAD knockout as one of the nodes in the combination. Only combinations that have an EMT % < 90% are shown in the table.

| **Node 1** | **Node 2** | **% EMT**  **(n=1000 simulations)** |
| --- | --- | --- |
| ERK = ON | SMAD = OFF | 2.8 |
| MEK = ON | SMAD = OFF | 6.9 |
| RAF = ON | SMAD = OFF | 13.4 |
| GSK3β = ON | SMAD = OFF | 35.0 |

**Supplemental Table 10: Differences between the EMT percentages obtained for ranked update probabilities versus equal update probabilities.** We test whether the EMT percentages for the ranked updating scheme for single perturbations, double perturbations, and triple knockouts are noticeably changed when assigning an equal update probability to each node. The table shows the scenarios wherein the magnitude of the relative change (%EMT_EQPROB_ - %EMT _RANKED_)/ %EMT_RANKED_ is greater than 0.2.

| **Node 1** | **Node 2** | **Node 3** | **% EMT_EQPROB_**  **(n=1000 simulations)** | **Relative change** |
| --- | --- | --- | --- | --- |
| SMAD=OFF | AKT=OFF | - | 72.7 | 1.11 |
| SMAD=OFF | ERK=ON | - | 7.2 | 1.57 |
| SMAD=OFF | GSK3B=ON | - | 76.6 | 1.19 |
| SMAD=OFF | MEK=ON | - | 20.6 | 1.99 |
| SMAD=OFF | PI3K=OFF | - | 73.4 | 1.10 |
| SMAD=OFF | RAF=ON | - | 34.8 | 1.60 |
| SMAD=OFF | RKIP=OFF | PI3K=OFF | 22.6 | 3.43 |
| SMAD=OFF | AKT=OFF | RKIP=OFF | 21.7 | 3.02 |
| SMAD=OFF | ILK=OFF | AKT=OFF | 75.2 | 1.47 |
| SMAD=OFF | SMO=OFF | AKT=OFF | 73.7 | 1.39 |
| SMAD=OFF | PI3K=OFF | Frizzled=OFF | 75 | 1.38 |
| SMAD=OFF | PI3K=OFF | LOXL23=OFF | 74 | 1.36 |
| SMAD=OFF | AKT=OFF | Frizzled=OFF | 74.9 | 1.36 |
| SMAD=OFF | PI3K=OFF | Dest_compl=OFF | 74.4 | 1.35 |
| SMAD=OFF | AKT=OFF | GLI=OFF | 72.7 | 1.33 |
| SMAD=OFF | IKKA=OFF | PI3K=OFF | 72.9 | 1.33 |
| SMAD=OFF | AKT=OFF | IKKA=OFF | 74.9 | 1.31 |
| SMAD=OFF | CD44=OFF | PI3K=OFF | 74.9 | 1.31 |
| SMAD=OFF | AKT=OFF | cfos=OFF | 75.3 | 1.31 |
| SMAD=OFF | Csn=OFF | AKT=OFF | 72.7 | 1.29 |
| SMAD=OFF | AKT=OFF | Dest_compl=OFF | 73.3 | 1.29 |
| SMAD=OFF | SRC=OFF | PI3K=OFF | 74.1 | 1.29 |
| SMAD=OFF | AKT=OFF | NFKB=OFF | 73.8 | 1.28 |
| SMAD=OFF | Wnt=OFF | PI3K=OFF | 75.1 | 1.28 |
| SMAD=OFF | AKT=OFF | Bcatenin_nuc=OFF | 74.1 | 1.27 |
| SMAD=OFF | PI3K=OFF | CHD1L=OFF | 73.2 | 1.27 |
| SMAD=OFF | ILK=OFF | PI3K=OFF | 74.1 | 1.27 |
| SMAD=OFF | LIV1=OFF | PI3K=OFF | 74.1 | 1.27 |
| SMAD=OFF | Wnt=OFF | AKT=OFF | 74.1 | 1.27 |
| SMAD=OFF | FUS=OFF | AKT=OFF | 74.2 | 1.26 |
| SMAD=OFF | DSH=OFF | PI3K=OFF | 76.4 | 1.26 |
| SMAD=OFF | AKT=OFF | cMet=OFF | 73.9 | 1.25 |
| SMAD=OFF | SHH=OFF | PI3K=OFF | 74.3 | 1.25 |
| SMAD=OFF | SMO=OFF | PI3K=OFF | 73.3 | 1.25 |
| SMAD=OFF | AKT=OFF | SRC=OFF | 74.2 | 1.24 |
| SMAD=OFF | AKT=OFF | TCF_LEF=OFF | 73.6 | 1.24 |
| SMAD=OFF | PI3K=OFF | Jagged=OFF | 72.5 | 1.23 |
| SMAD=OFF | GLI=OFF | PI3K=OFF | 74.1 | 1.23 |
| SMAD=OFF | PI3K=OFF | TCF_LEF=OFF | 74.5 | 1.22 |
| SMAD=OFF | AKT=OFF | LIV1=OFF | 76.7 | 1.22 |
| SMAD=OFF | AKT=OFF | STAT=OFF | 74.6 | 1.21 |
| SMAD=OFF | PI3K=OFF | HIF1A=OFF | 74.5 | 1.21 |
| SMAD=OFF | cfos=OFF | PI3K=OFF | 73.6 | 1.20 |
| SMAD=OFF | cMet=OFF | PI3K=OFF | 72.5 | 1.19 |
| SMAD=OFF | AKT=OFF | AXIN2=OFF | 74.5 | 1.18 |
| SMAD=OFF | AKT=OFF | PI3K=OFF | 73.5 | 1.17 |
| SMAD=OFF | PI3K=OFF | Bcatenin_nuc=OFF | 73.5 | 1.17 |
| SMAD=OFF | NFKB=OFF | PI3K=OFF | 74.3 | 1.16 |
| SMAD=OFF | AKT=OFF | EGR1=OFF | 74.5 | 1.13 |
| SMAD=OFF | FUS=OFF | PI3K=OFF | 74.2 | 1.13 |
| SMAD=OFF | STAT=OFF | PI3K=OFF | 74.7 | 1.12 |
| SMAD=OFF | AKT=OFF | HIF1A=OFF | 71.7 | 1.12 |
| SMAD=OFF | PI3K=OFF | BTrCP=OFF | 78.3 | 1.11 |
| SMAD=OFF | AKT=OFF | Jagged=OFF | 72.4 | 1.10 |
| SMAD=OFF | AKT=OFF | CHD1L=OFF | 72.4 | 1.09 |
| SMAD=OFF | AKT=OFF | LOXL23=OFF | 73.4 | 1.09 |
| SMAD=OFF | EGR1=OFF | PI3K=OFF | 73.8 | 1.08 |
| SMAD=OFF | AKT=OFF | BTrCP=OFF | 77.9 | 1.07 |
| SMAD=OFF | PI3K=OFF | AXIN2=OFF | 73.5 | 1.07 |
| SMAD=OFF | SHH=OFF | AKT=OFF | 72.5 | 1.07 |
| SMAD=OFF | PI3K=OFF | miR200=OFF | 79.8 | 1.06 |
| SMAD=OFF | AKT=OFF | CD44=OFF | 74.2 | 1.05 |
| SMAD=OFF | DSH=OFF | AKT=OFF | 72.5 | 1.03 |
| SMAD=OFF | Csn=OFF | PI3K=OFF | 71.4 | 1.01 |
| SMAD=OFF | AKT=OFF | miR200=OFF | 74.7 | 0.93 |
| SMAD=OFF | AKT=OFF | PAK1=OFF | 69.9 | 0.90 |
| SMAD=OFF | PI3K=OFF | CDC42=OFF | 68.4 | 0.79 |
| SMAD=OFF | PAK1=OFF | PI3K=OFF | 69.8 | 0.78 |
| SMAD=OFF | AKT=OFF | CDC42=OFF | 69.2 | 0.76 |
| SMAD=OFF | RKIP=OFF | BTrCP=OFF | 32.5 | -0.21 |
