# Supplementary figures and images for: Combinatorial interventions inhibit TGFβ-driven epithelial-to-mesenchymal transition and support hybrid cellular phenotypes
Source: NPJ Syst Biol Appl. 2015 Nov 26;1:15014–. doi: 10.1038/npjsba.2015.14 (PMC5516807; doi:10.1038/npjsba.2015.14)

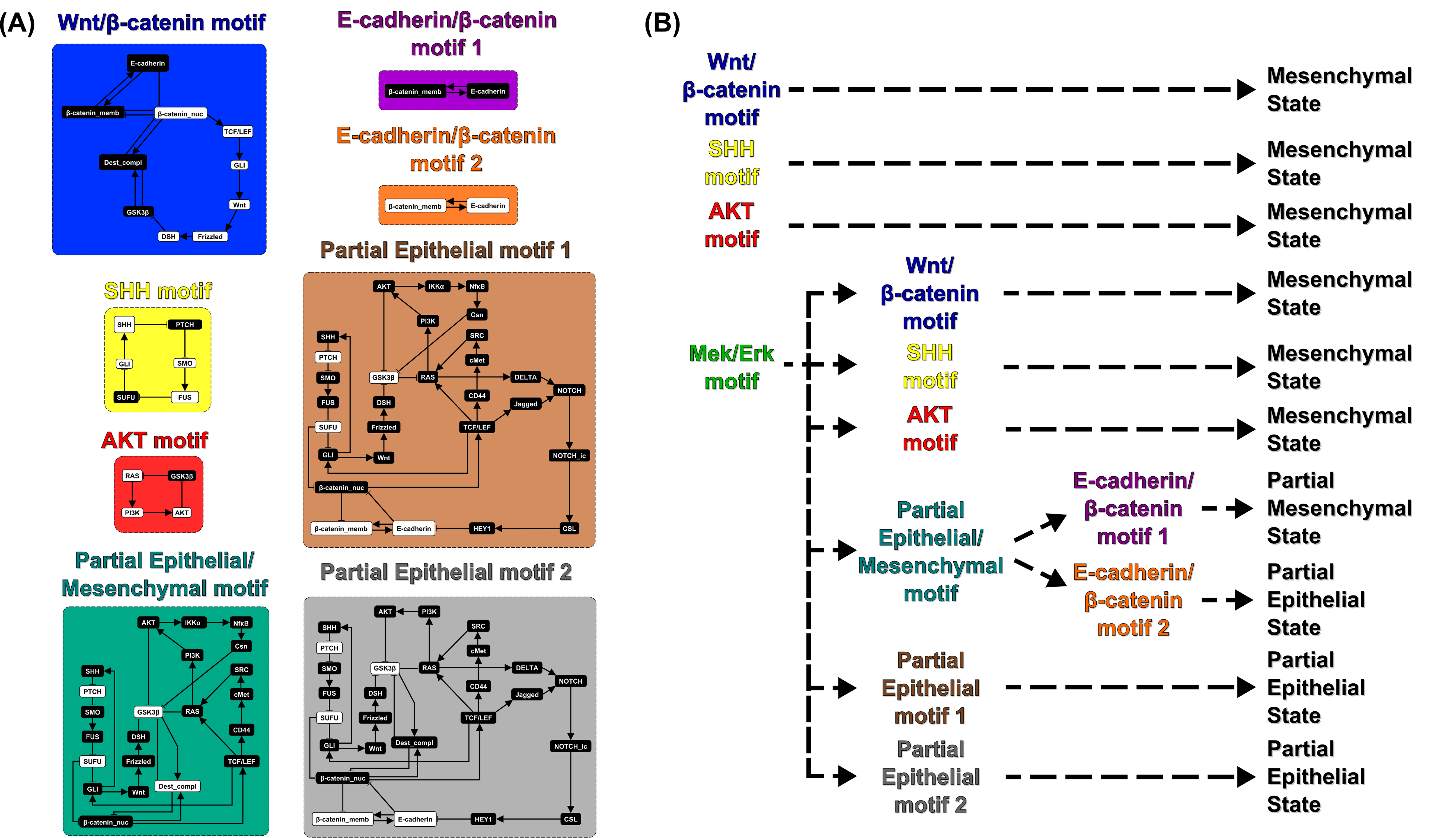

Supplement: Supplementary Figure 1 [file npjsba201514-s3.tiff]

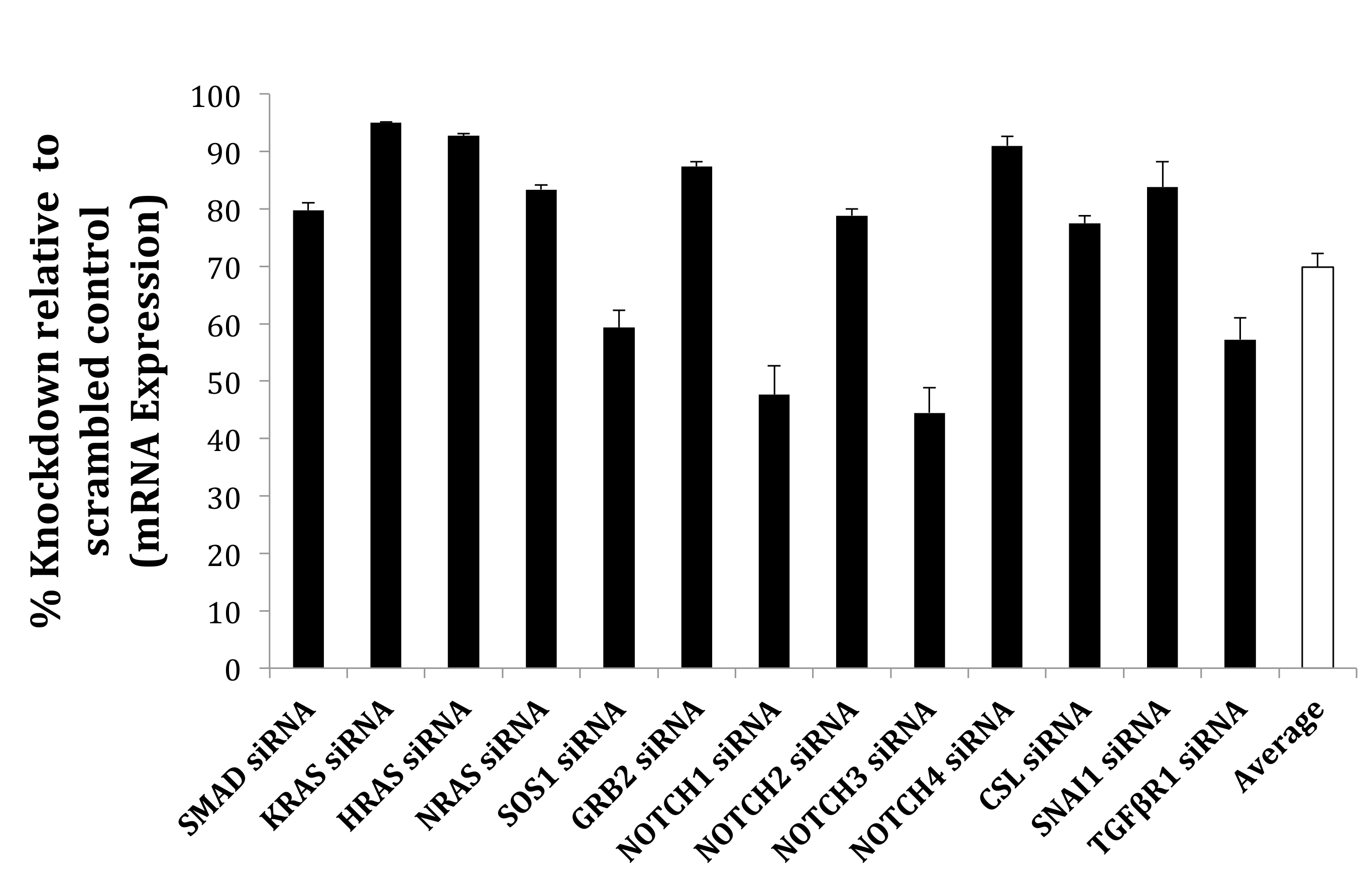

Supplement: Supplementary Figure 2 [file npjsba201514-s4.tiff]

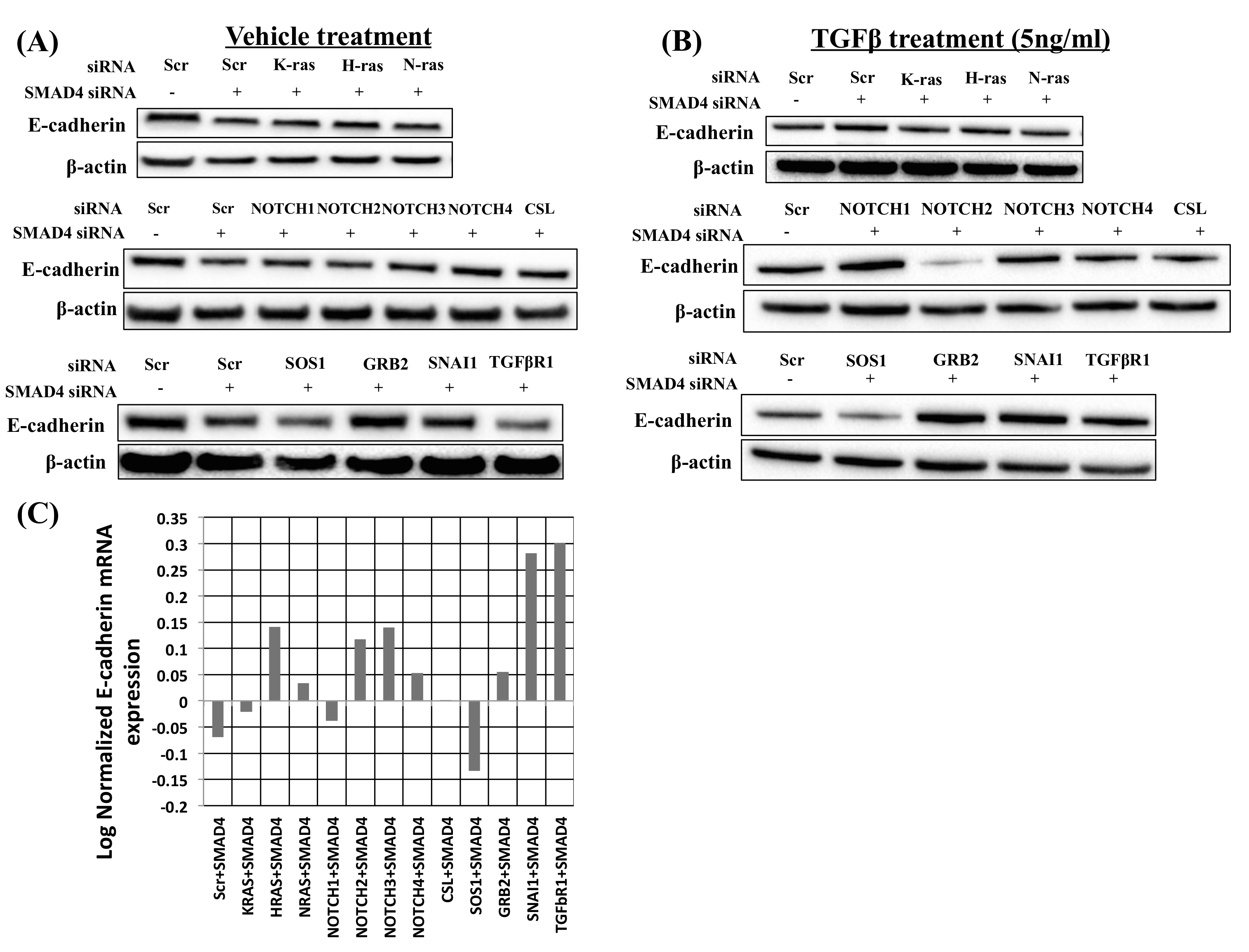

Supplement: Supplementary Figure 3 [file npjsba201514-s5.tiff]

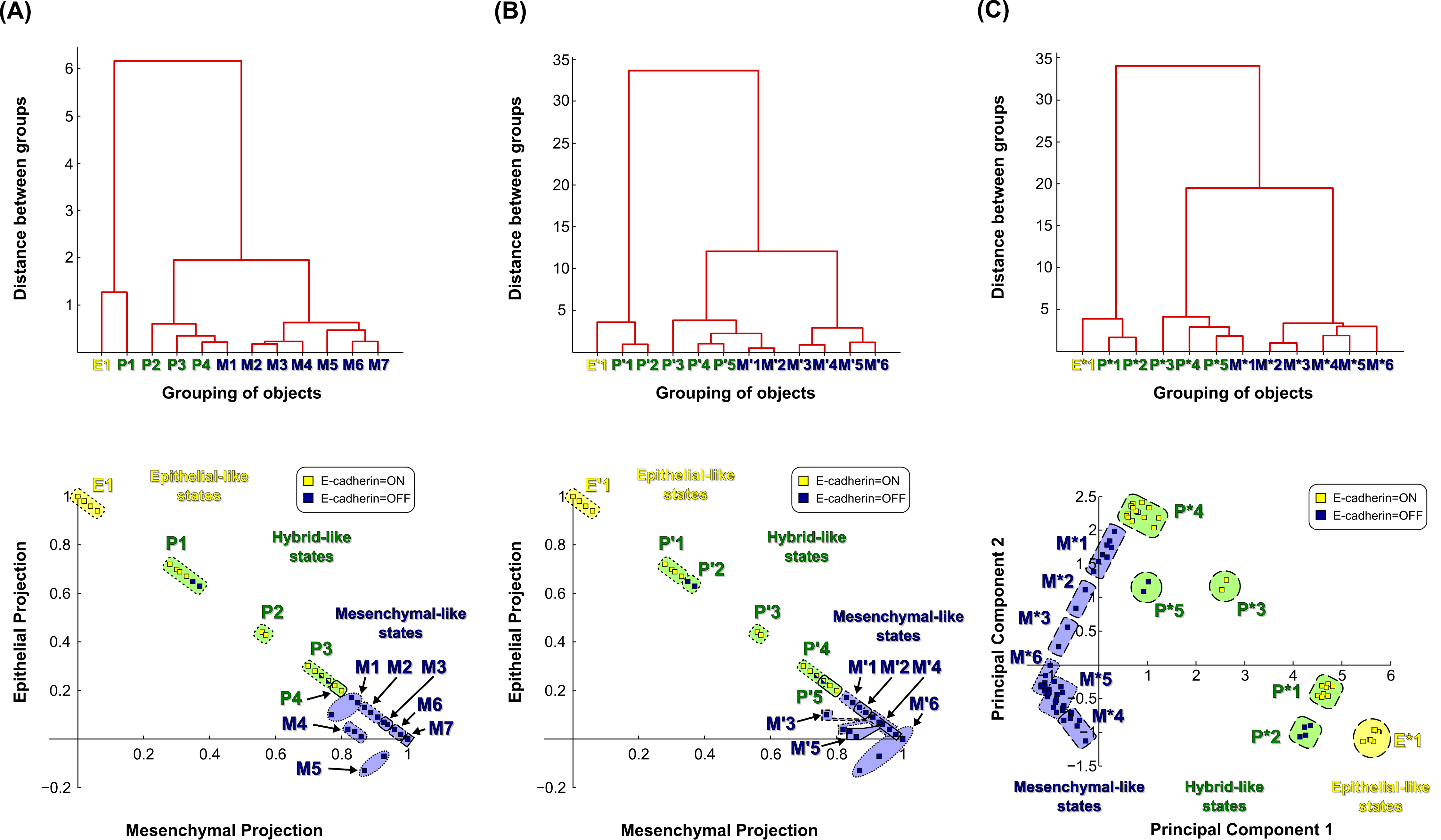

Supplement: Supplementary Figure 4 [file npjsba201514-s6.tiff]

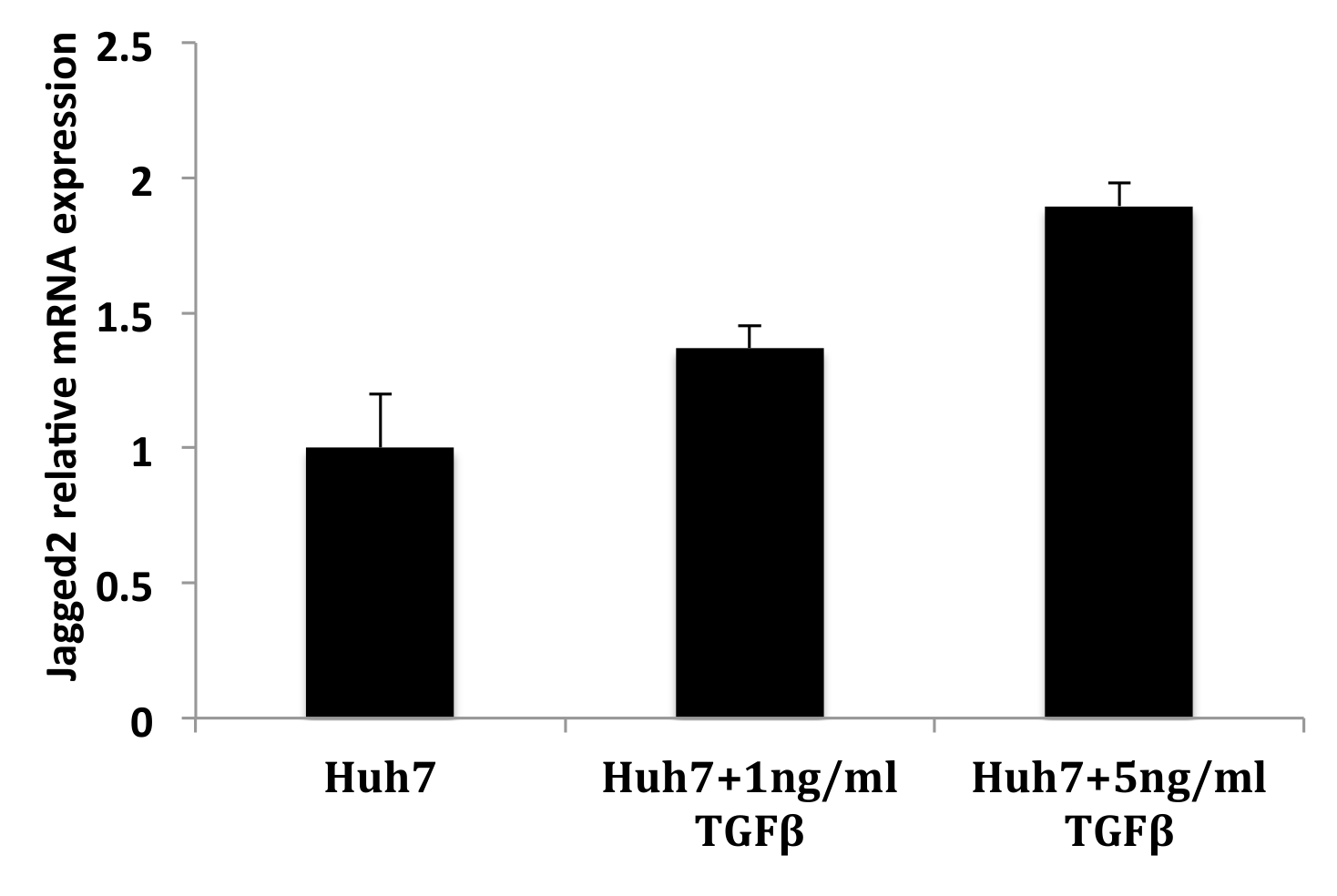

Supplement: Supplementary Figure 5 [file npjsba201514-s7.tiff]

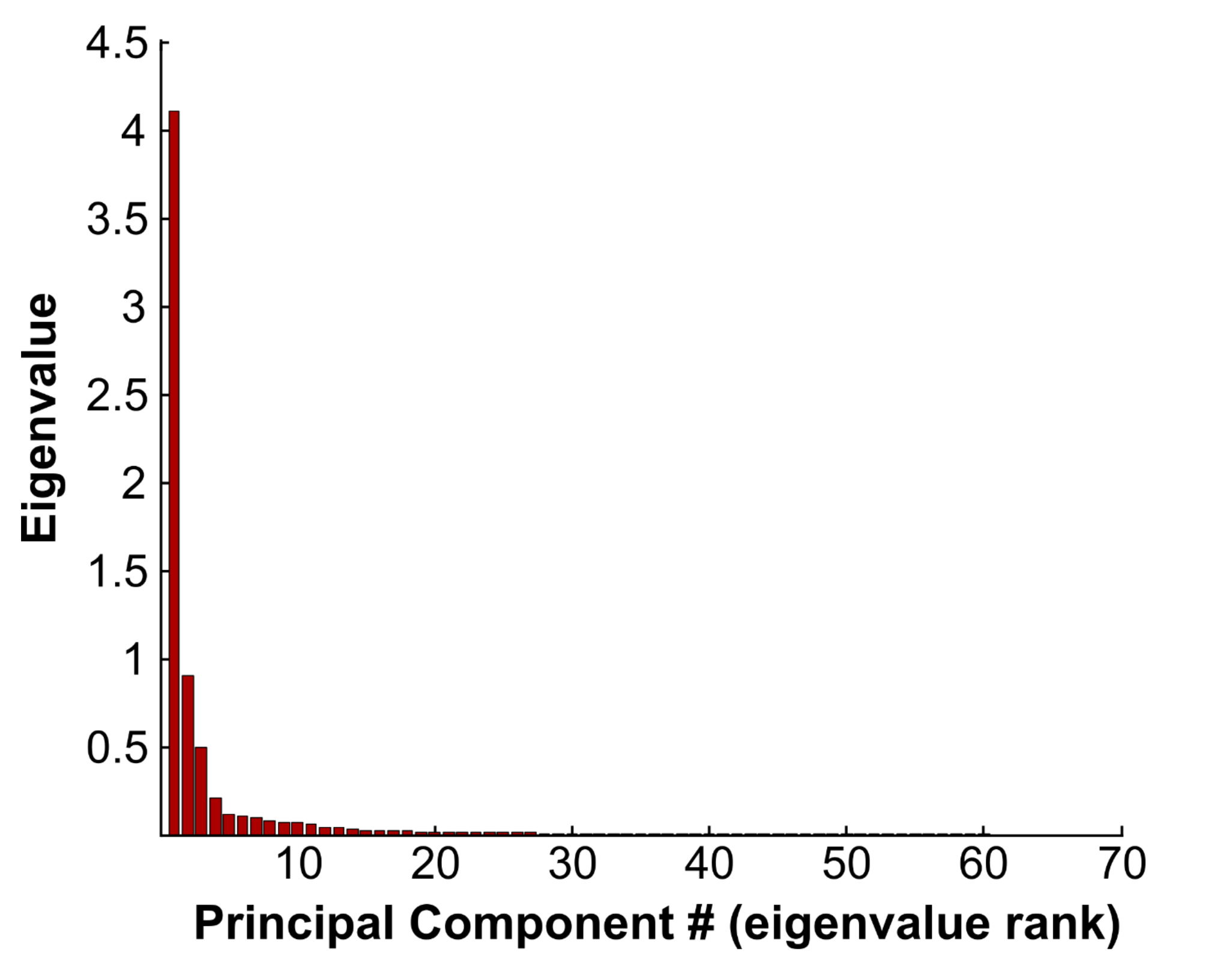

Supplement: Supplementary Figure 6 [file npjsba201514-s8.tiff]
